# PI3Kδ is selectively inhibited by roginolisib by stabilizing the C-terminal helix kα12

**DOI:** 10.1038/s41467-026-77424-0

**Published:** 2026-10-09

**Authors:** Oscar Vadas, Simon Tiede, Maria Chaouki, Laura Tesmer, Shanlin Rao, Rémy Visentin, Ulrich Grädler, Martin Augustin, Reiner Kiefersauer, Carola Gößer, Anne Quillet-Mary, Loïc Ysebaert, Julie Guillermet-Guibert, Lars van der Veen, Giusy Di Conza, Gerhard Hummer

**Affiliations:** 1 Protein Biochemistry Platform, Faculty of Medicine, University of Geneva, Geneva, Switzerland; 2 Department of Theoretical Biophysics, Max Planck Institute of Biophysics, Frankfurt am Main, Germany; 3 Centre de Recherches en Cancérologie de Toulouse, Inserm, CNRS, Université de Toulouse, Labex Toucan, Toulouse, France; 4 Merck Healthcare KGaA, Darmstadt, Germany; 5 Proteros biostructures GmbH, Planegg-Martinsried, Germany; 6 Service d’Hématologie, IUCT-Oncopole, Toulouse, France; 7 iOnctura SA, Geneva, Switzerland; 8 Institute of Biophysics, Goethe University Frankfurt, Frankfurt am Main, Germany

**Keywords:** Mass spectrometry, Computational biophysics, Target validation, X-ray crystallography

## Abstract

Phosphoinositide 3-kinases (PI3Ks) are major regulators of cell growth, proliferation and signalling, constituting key therapeutic targets in cancer, inflammation, and other diseases. Individual class I PI3K isoforms control key cellular functions, imposing the need to generate isoform-specific inhibition for therapeutic intervention. Roginolisib is a selective PI3Kδ inhibitor that shows promise for the treatment of cancer. Using a combination of X-ray crystallography, molecular dynamics simulations, and hydrogen-deuterium exchange mass spectrometry, we describe the mechanism driving roginolisib’s potent and isoform-selective inhibition of PI3Kδ. Roginolisib uniquely stabilises the catalytic C-terminal helix kα12, locking the enzyme in an inactive conformation. This binding mode also results in more sustained inhibition of phosphatidylinositol 3,4,5-trisphosphate formation in tumour samples of CLL patients. Inhibition of PI3Ks by stabilization of helix kα12 into an inactive conformation has not, to the best of our knowledge, been described before and may provide the basis for novel, more selective and effective pharmacological strategies.

## Abstract (web summary)

In this work. Vadas et al. report how Roginolisib, a phosphoinositide 3-kinase delta specific inhibitor, locks the enzyme in an inactive conformation, providing high potency and specificity. This binding mode results in reduced phosphatidylinositol 3,4,5-trisphosphate formation in tumour samples of CLL patients receiving Roginolisib.

## Introduction

Phosphoinositide 3-kinases (PI3Ks) are lipid-modifying enzymes that phosphorylate the 3’-hydroxyl group of inositol phospholipids. They are involved in a number of physiological processes crucial for cell survival, growth, and differentiation^1^. Class I PI3Ks are receptor-activated heterodimeric enzymes that are composed of a catalytic and a regulatory subunit. They convert phosphatidylinositol-4,5-bisphosphate (PIP2), which is mainly found at the intracytoplasmic face of the cell membrane, into phosphatidylinositol-3,4,5-trisphosphate (PIP3). The class IA catalytic subunits p110α, p110β, and p110δ are associated with p85 regulatory subunits and mediate stimulation downstream of receptor-tyrosine kinases (RTKs) and G protein-coupled receptors (GPCRs)^2^. The only Class IB catalytic subunit, p110γ, forms heterodimers with p84 or p101 regulatory subunits, mediating signals downstream of GPCRs. Activation of the kinase Akt through PIP3 leads to downstream processes that include inhibition of apoptosis and activation of protein synthesis. The PI3K pathway is among the most frequently activated in human cancer^3^, making the different isoforms attractive pharmacological targets for development of isoform-specific inhibitors^4^.

The catalytic subunit of the PI3Kδ isoform, p110δ, is encoded by the PIK3CD gene^5^ and in contrast to the other class IA PI3Ks, p110α and p110β, p110δ is preferentially expressed within immune cells^6^. PI3Kδ signaling regulates activation and differentiation of immune cells^7^, and inactivation of PI3Kδ has been shown to impact the function of T cells, B cells, mast cells and neutrophils^1^. Based on the pivotal role of PI3Kδ for the development and expansion of malignant B-cells, several PI3Kδ inhibitors have been developed^8^.

Idelalisib (Zydelig) was the first PI3Kδ inhibitor developed for the treatment of B-cell malignancies. Its efficacy has been demonstrated in several clinical studies, resulting in its approval by the United States Food and Drug Administration for treatment of patients with relapsed chronic lymphocytic leukaemia (CLL) and indolent non-Hodgkin lymphoma (NHL)^9^. Unfortunately, idelalisib and similar first generation PI3Kδ inhibitors have been associated with variable degrees of toxicity, ultimately leading to withdrawal of the accelerated approvals received for their use in NHL^10^.

The factors responsible for the toxicity of these first-generation inhibitors are not fully understood. However, insufficient isoform selectivity is likely a major contributor^11,12^. Achieving high selectivity among PI3K isoforms remains challenging due to the conserved catalytic site. Targeting regions away from the ATP binding pocket, for example using allosteric modulators, holds promise^13^. In addition, the complex regulatory mechanisms controlling PI3K activation provide opportunities to interfere with kinase activity “at a distance” and possibly, with enhanced isoform selectivity^14^.

PI3Kδ is autoinhibited by reversible inhibitory contacts of the nSH2 and cSH2 domains of the p85 regulatory subunit with the C2, helical and kinase domains of p110δ^14–16^. Localization of PI3Kδ to the membrane is promoted by Ras binding to the Ras binding domain (RBD) of p110δ^17^. At the membrane, PI3Kδ is activated by phosphorylated tyrosine (pY) motifs of RTKs that have high affinity binding to the p85 nSH2/cSH2 domains, releasing their autoinhibitory activity. For full activation of PI3Kδ at the membrane, a conformational change is also required in the regulatory arch spanning the p110δ catalytic domain involving helices kα10, kα11 and kα12, which must change from a closed to an open state^14,17^. Each of these steps towards full activation of the kinase and the conformational changes involved may offer opportunities for isoform-specific inhibition.

Roginolisib is a novel, small molecule inhibitor that selectively targets the PI3Kδ isoform and is currently evaluated in Phase II trials. It was investigated in a first-in-human (FIH) dose study, where it showed a favourable metabolic and pharmacokinetic profile, resulting in high target specificity and a promising safety profile, distinguishing it from first generation PI3Kδ inhibitors^12,18^. Unlike idelalisib, roginolisib has been shown to retain its potency, even at high ATP concentrations^19^. We therefore hypothesized that the binding mode of roginolisib to PI3Kδ might be unique, potentially accounting for its distinct safety profile observed in the clinic. To understand how this drug achieves high isoform selectivity, we aimed to investigate the differences in PI3Kδ binding interactions between roginolisib and idelalisib, using a combination of X-ray crystal structure analysis, molecular dynamics (MD) simulations, and hydrogen-deuterium exchange mass spectrometry (HDX-MS). Additionally, we explored potential differences in the inhibition of PIP3 formation in CLL patient samples.

In this work, we identify an inhibitor binding mode to PI3Kδ that induces conformational changes and locks the kinase into an inactive state by tethering basic residues at the C-terminal end of helix kα12, leading to a more efficacious inhibition of PIP3 formation. Strong interactions with isoform-specific regulatory elements at the C-terminus may explain the comparably high selectivity of the inhibitor.

## Results

### X-ray crystal structures show distinct binding modes of roginolisib and idelalisib

**Fig. 1:**
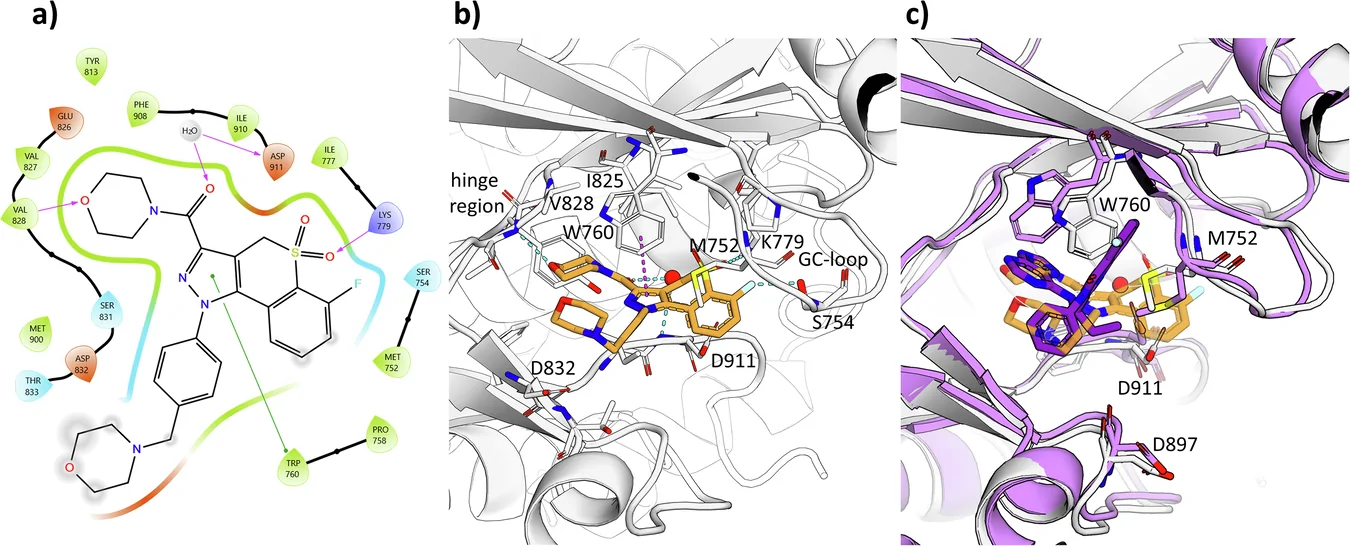
Binding interactions of roginolisib within the protein active site. **a** Roginolisib forms hydrogen bonds with V828 and K779, a solvent-mediated hydrogen bond with D911, and π-π stacking with W760. **b** Crystal structure of human PI3Kδ in complex with roginolisib (orange), solved at 2.75 Å resolution (PDB-ID: 9T07). Key interactions include hydrogen bonds (dashed cyan lines) with V828 (hinge), K779, S754 (via aromatic fluorine), and the backbone NH of D911 (mediated by a water molecule), as well as π-π-interactions (dashed magenta line) with W760. **c** Superimposition of human PI3Kδ (grey)/roginolisib (orange) (PDB-ID: 9T07) and murine PI3Kδ/idelalisib (PDB-ID: 4XE0, purple) highlights the distinct binding mode of idelalisib, which targets the lipophilic pocket between M752 and W760 and the different orientations of D897 and D911 in both structures.

In order to elucidate the differences in PI3Kδ binding interactions between roginolisib and idelalisib we investigated the co-crystal structure of the roginolisib-PI3Kδ complex at 2.75 Å resolution (PDB-ID: 9T07, Fig. 1a and b, Supplementary Fig. 1, Supplementary Table 1). The solved crystal structure revealed complete electron density for roginolisib within the ATP-binding pocket and indicates an H-bond contact between the morpholine O-atom and the backbone NH of Val828, which belongs to the hinge region. In addition, roginolisib forms π-stacking interactions via the pyrazole ring to Trp760 and extensive van-der-Waals (vdW) contacts to Met752, Pro758, Ile777, Tyr813, Ile825, Thr833, Met900 and Phe908^20^.

The binding modes of roginolisib and idelalisib (Fig. 1c) are markedly different. Whereas both inhibitors form a hydrogen bond (3.0 and 2.9 Å distance, respectively) to Val828 in the hinge region of the ATP pocket, roginolisib does not occupy the so called PI3Kδ “specificity” pocket at the interface between the side chains of Met752, Pro758, Trp760 and Ile777^21^. Instead, roginolisib engages the Met752 and Trp760 residues that shape the specificity pocket in vdW and π-π-interactions, reorienting the sidechains, compared to the complex with idelalisib. Furthermore, the sulphone in the core structure of roginolisib forms a hydrogen bond to Lys779 (3.1 Å distance) and the aromatic fluorine-atom a fluor hydrogen bond (2.9 Å distance) to Ser754, both residues not addressed by idelalisib. Another notable difference is that Asp897 forms a strong (2.6 Å distance) hydrogen bond with His895 of the DRH motif in the roginolisib structure, whereas in the idelalisib structure His895 forms a hydrogen bond (3.3 Å distance) with Asp893. Also, both inhibitors interact differently with Asp911. The carboxamide of roginolisib is part of a hydrogen bonding network involving a water molecule in the ATP pocket and the backbone NH of Asp911 (Fig. 1b). This water molecule is also engaged by the core imidazopyrimidine structure of idelalisib, however, the second hydrogen bond is formed with the side chain carboxylate of Asp911 and not with its backbone NH (Fig. 1c).

As part of the conserved DFG motif within the kinase activation loop, Asp911 is expected to coordinate the magnesium ion in kinases^22,23^. The distinction between a “DFG-in” and “DFG-out” conformation has been reported to influence ligand selectivity and binding affinity^24^. In the DFG-in conformation, the aspartate is oriented towards the active site with the phenylalanine in the opposite direction, whereas the orientation of the two residues is reversed in the DFG-out conformation^25,26^. While no apparent difference was observed in the phenylalanine orientation, which is in the DFG-in conformation for both structures, the DFG motif’s aspartate adopted alternative conformations in the presence of roginolisib and idelalisib (Fig. 1c, Supplementary Fig. 2). The binding of roginolisib pushes Asp911 into a DFG-in-like configuration. By contrast, idelalisib leaves the residue in a rotated (by 90°) orientation resembling the DFG-out aspartate conformation.

To confirm if the observed differences in binding interactions between idelalisib and roginolisib are consistent, the co-crystal structure of PI3Kδ in complex with a close analogue of roginolisib was solved at 2.97 Å resolution (PDB-ID: 9T1P). In this crystal structure identical binding interactions to roginolisib were observed. To investigate if the different binding interaction results in different binding kinetics between idelalisib and roginolisib, the binding on- and off-rates were determined by surface plasmon resonance (SPR)^27^. The data showed that roginolisib has an approximately 3-fold lower association-rate k_a_ (1.49E+06 vs 5.05E+06/(M s)) and 9-fold higher dissociation-rate k_d_ (2.98E-02 vs 3.17E-03/s) than idelalisib, resulting in an approximately 30-fold higher K_D_ (20.1 nM vs 0.63 nM) (Supplementary Fig. 3).

### Molecular dynamics simulations show that roginolisib reorients catalytic kα11/kα12 helices towards the active site

As a major limitation of the roginolisib crystal structure, only the catalytic p110δ subunit and the p85α iSH2 domain were crystallized and no electron density was obtained for the C-terminal kα12 helix. This has also been the case with other published co-crystal structures of inhibitor-bound PI3Kδ. To investigate the impact of the different inhibitor binding modes on the conformation and dynamics of the p110δ C-terminal region, we modelled the unresolved protein regions based on structural prediction by AlphaFold^28^. To validate the modelled full-length p110δ structure, we compared it to the murine p110β/p85β-icSH2 crystal structure (PDB-ID: 2Y3A, Supplementary Fig. 4), for which the kα12 helix has been partially resolved in its inactive conformation^29^. The modelled p110δ kα12 helix proved to align well, suggesting that the full-length p110δ model also represents an inactive protein conformation (Supplementary Fig. 4). All-atom MD simulations were conducted on full-length human p110δ bound to either roginolisib, idelalisib or ATP. These simulations confirmed stable binding of all three molecules within the ATP pocket of PI3Kδ (Supplementary Fig. 5a-c, Supplementary Movies 1-3). We further assessed key ligand–receptor interaction fingerprints for roginolisib, namely the two hydrogen bonds formed by roginolisib in the crystal structure (Supplementary Fig. 5d, e). The hydrogen bond between the morpholine oxygen and Val828 remained highly stable throughout the simulations, supporting conservation of the crystallographic binding mode. Hydrogen bonding between a sulphone oxygen and Lys779, whilst weaker and more dynamic, was consistently observed in all replicates. Its fluctuations arose from rotations of the Lys779 side chain, which occasionally swung away before returning to reform the interaction. This behaviour reflects flexibility in the lysine side chain whilst the ligand binding pose remains stable.

**Fig. 2:**
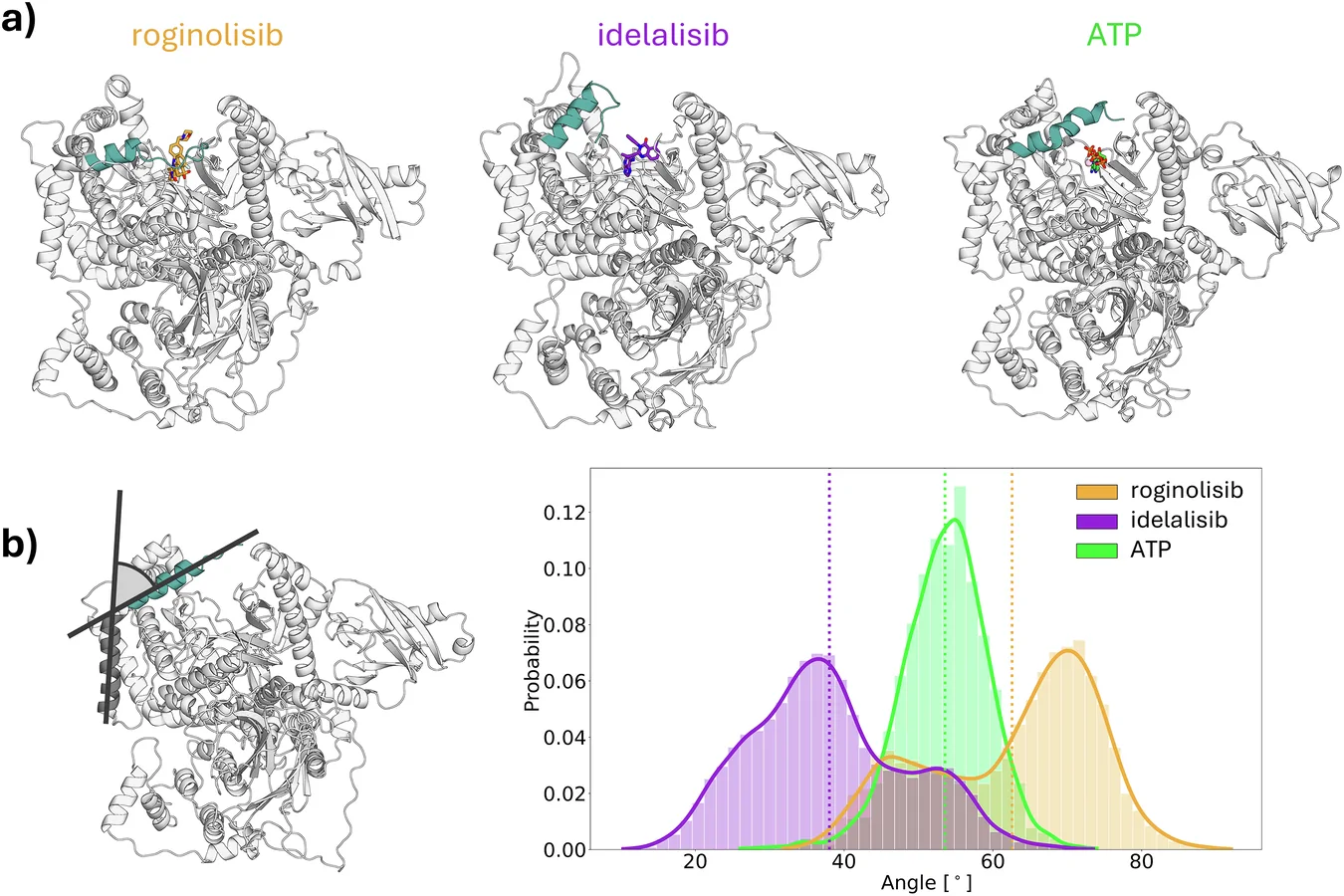
Difference in angle between PI3Kδ helix kα11 and the C-terminal helix kα12 in the presence of alternative bound ligands. **a** Representative simulation snapshots of roginolisib- (orange), idelalisib- (purple) and ATP- (green) bound structures with the C-terminal kα12 helix highlighted in teal. **b** Probability distribution of the angle formed between helix kα11 (grey) and kα12 (teal) across five replicates. The dotted lines represent the mean angle, corresponding to (62 ± 12) in the presence of roginolisib, (38 ± 11) with idelalisib and (54 ± 6) with ATP.

Surprisingly, consistent differences were observed in the conformational dynamics of the C-terminus between the different ligand-bound states, across five replicate simulations of each structure (Fig. 2a). In the ATP-bound form of the protein, the C-terminal helix kα12 remained stable in its initial orientation relative to kα11. However, in the presence of the two inhibitors the angle between the two helices exhibited distinct shifts (Fig. 2b). In multiple simulation replicates (2–5 μs in length) of the roginolisib-bound protein, kα12 further levered towards the kinase active site. The C-terminal half of kα12 showed unfolding, whereas the N-terminal half maintained its α-helical secondary structure. In clear contrast, kα12 levered away from the idelalisib-bound protein, ultimately resulting in a ~ 35° difference between the most probable kα11-kα12 angle of the roginolisib- and idelalisib-bound state (Fig. 2b). The simulated effect of roginolisib on helix kα12 is consistent with the reported auto-inhibitory role of the C-terminus in the absence of lipid substrate, whereby kα12 was proposed to impede ATP hydrolysis by folding over the active site^15,30,31^. With membrane association of kα12 and its concomitant repositioning proposed to be required for PI3K activation^31–33^, the inhibitory effect of roginolisib may stem from its ability to promote a pronounced bend of the kα11/kα12 configuration, sequestering kα12 away from substrate binding at the membrane.

**Fig. 3:**
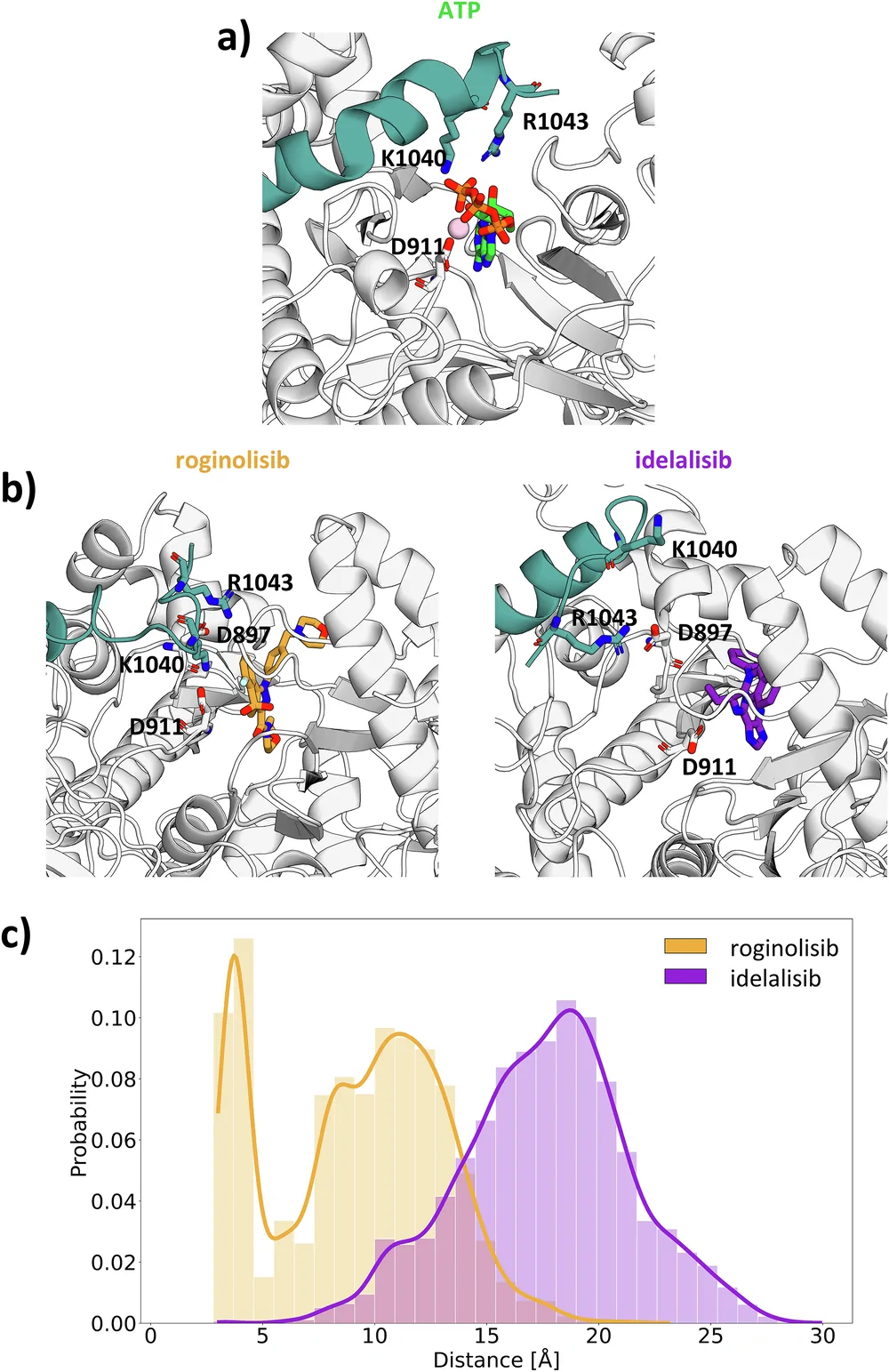
Specific interactions formed by basic residues of C-terminal kα12 helix in the presence of different ligands. **a** Close-up view of the ATP (green) binding site in a representative simulation snapshot, with the C-terminal helix kα12 highlighted in teal. Basic C-terminal helix residues Lys1040 and Arg1043, shown as sticks, interact with the γ-phosphate of ATP, with Asp911 of the DFG motif coordinating the magnesium ion (pink). **b** (left) Binding site of roginolisib (orange), where Lys1040 and Arg1043 form interactions with Asp897 and Asp911. The positioning and conformation of roginolisib leads to a configuration of Asp911 that is oriented towards the C-terminal helix. (right). The idelalsisib (purple) binding site. Arg1043 forms interaction with Asp897, with no interactions between Lys1040 and Asp911. **c** Probability distribution of the distance between the side-chain nitrogen atom of Lys1040 and carboxyl carbon of Asp911 across replicates, excluding the first 500 ns as equilibration time.

Examination of the simulated ligand-bound p110δ structures revealed that two basic residues within helix kα12, Lys1040 and Arg1043, formed distinct contacts in the presence of different bound ligands. In the ATP-bound protein, Lys1040 and Arg1043 interacted with the γ-phosphate of ATP, likely with a stabilising effect on ATP binding and a possible role in the process of PIP2 phosphorylation. The magnesium ion was coordinated by Asp911 of the DFG motif forming part of the kinase activation loop (Fig. 3a).

In the presence of roginolisib, Asp911 was pushed sterically into proximity with Asp897 (Fig. 3b) to form a negatively charged cluster. Both aspartate residues maintained strong electrostatic interactions with one or both basic kα12 residues Lys1040 and Arg1043 (Fig. 3c). This led to stabilisation of the protein conformation with helix kα12 close to the active site. By contrast, in the presence of idelalisib Asp911 pointed away from Asp897, and no aspartate cluster was formed. Arg1043 was in sporadic contact with Asp897 in the idelalisib-bound state, whilst neither Lys1040 nor Asp911 formed any considerable interactions. Lys1040 was oriented towards the solvent for most of each simulation replicate (Fig. 3b).

### Hydrogen/deuterium exchange mass spectrometry analysis of heterodimeric p110δ/p85α confirms stabilisation of the C-terminal kα12 helix upon roginolisib binding

**Fig. 4:**
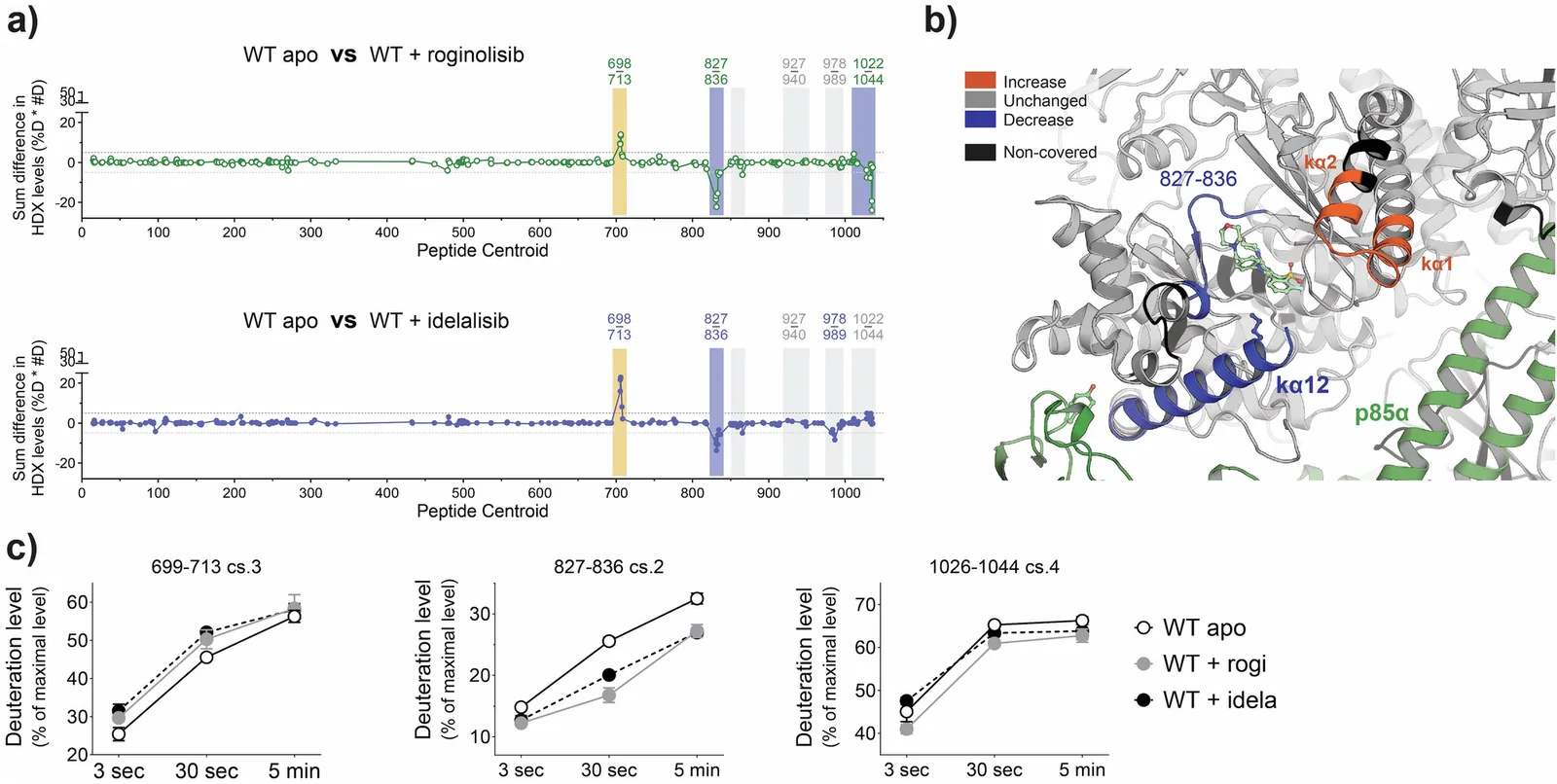
Identification of conformational changes on p110δ triggered by roginolisib and idelalisib binding to the p110δ/p85α complex using HDX-MS. **a** Comparison of the H/D exchange rate of p110δ peptide between the apo p110δ/p85α heterodimer (WT) and roginolisib-bound complex (WT + roginolisib) (top) and with idelalisib-bound complex (WT + idelalisib) (bottom). Each dot depicts a peptide, positioned based on the central amino acid (centroid). The sum of differences measured in deuterons (Δ#D) multiplied by the sum of percentage differences (Δ%D) across all deuteration times is calculated and plotted on the y-axis. Regions showing significant difference in deuterium exchange (defined as |Δ#D * Δ%D| > 5) are coloured on the graph in orange (deprotection) or blue (protection) **b** Model of p110δ/p85α heterodimer bound to roginolisib highlighting regions differentially deuterated upon roginolisib binding (coloured as shown in the legend). The p85α regulatory subunit is shown in green. **c** Uptake plots of a representative selection of p110δ peptides showing deuterium incorporation at three deuteration times for each of the studied conditions: WT p110α/p85α heterodimer in i) apo state (WT apo); ii) when bound to roginolisib (WT + rogi) and iii) when bound to idelalisib (WT + idela). Data shown as percentage deuteration (mean +/- SD, *n* = 3). cs = charge state.

To follow up on the prediction that roginolisib affects the kα12 helix conformation, we set out to study the dynamics of full-length p110δ/p85α upon inhibitor binding, in solution. We turned to HDX-MS which has previously shown its strength for studying PI3Ks structures^15,32,34^. Recombinant human PI3Kδ (p110δ/p85α) produced from insect cells was used and three different deuterium labelling conditions were compared to identify potential conformational changes upon inhibitor binding: i) PI3Kδ, ii) PI3Kδ + roginolisib, and iii) PI3Kδ + idelalisib. From a total of 283 peptides encompassing the p110δ subunit, four regions showed differential H/D exchange rates upon inhibitor binding, encompassing residues 698-713, 827-836, 978-989 and 1022–1044 (Fig. 4a, Supplementary Fig. 6, Supplementary Table 2, Supplementary Data 1, 2). Most of the protein remained unchanged, highlighting the minimal impact of inhibitor binding on overall PI3Kδ conformation. As expected, binding of roginolisib and idelalisib each protected the hinge region from H/D exchange (827–836), confirming their binding to the ATP site. In addition, both compounds similarly deprotected region 698–713, which contains the loop between helix kα1-kα2 in the kinase domain, a region involved in membrane interaction (Fig. 4b)^15^. This modification highlights a conformational change of a loop distant from the inhibitor binding site, which may contribute to the inhibitory mechanism of these compounds. Uniquely, binding of roginolisib protected the C-terminal helix kα12 from H/D exchange (region 1022–1044), indicating either direct interaction with this region, and/or stabilisation of the secondary structure (Fig. 4a,c). Identification of this protection is in agreement with the folding of kα12 over the active site and thus supports the results of the MD simulation and a different mode of inhibition between roginolisib and idelalisib.

### Roginolisib and idelalisib share a similar binding mode to the activated PI3Kδ heterodimer

**Fig. 5:**
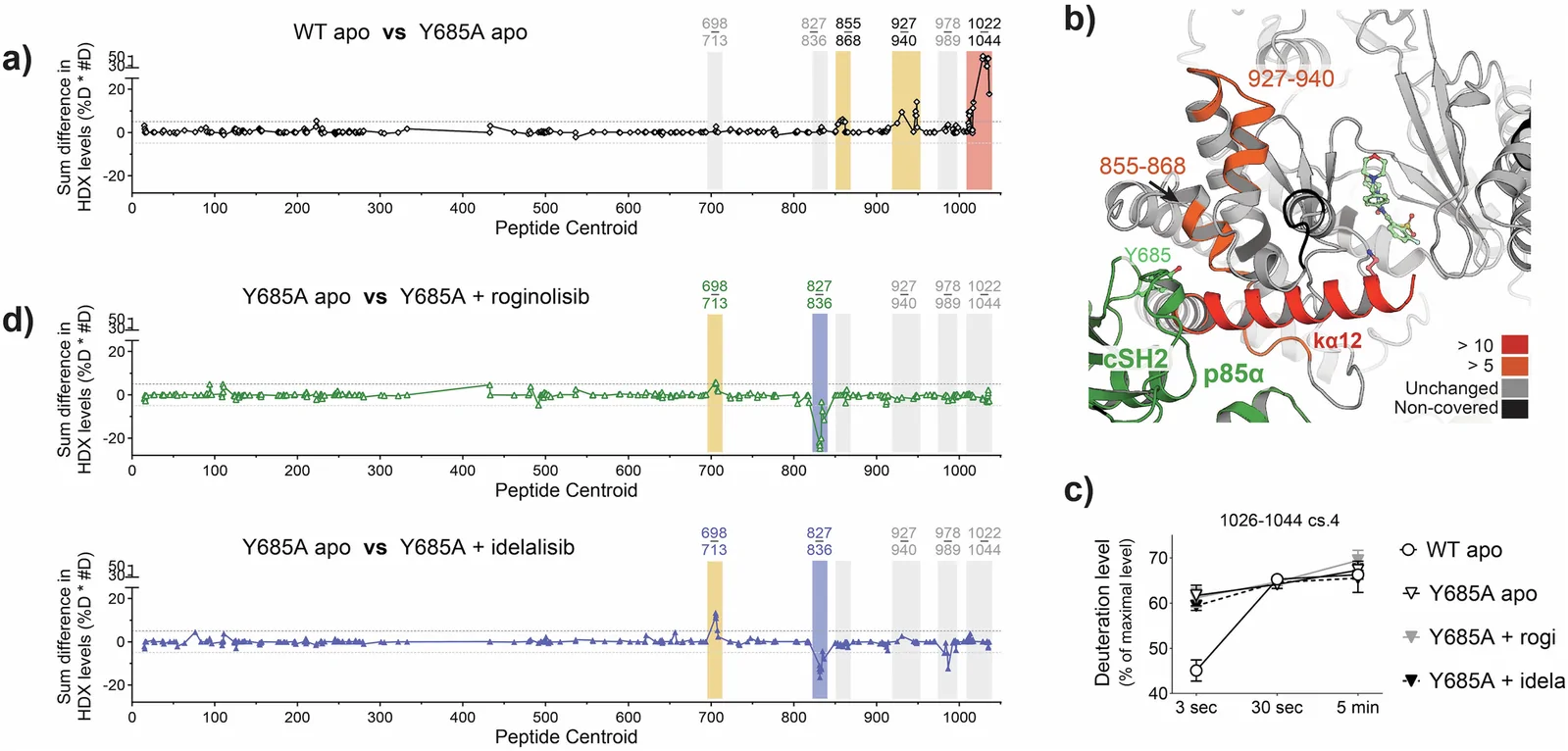
Roginolisib binding to activated p110δ/p85α-Y685A does not protect helix kα12 from exchange. **a** Comparison of H/D exchange rates in p110δ peptides between WT and Y685A PI3Kδ in absence of inhibitors (apo). Each dot depicts a peptide, positioned based on the central amino acid (centroid). The sum of deuteron differences (Δ#D) multiplied by the sum of percentage differences (Δ%D) across all deuteration times is plotted on the y-axis. Regions showing significant difference in deuterium exchange (defined as |Δ#D * Δ%D| > 5) are coloured on the graph in orange (deprotection >5), red (deprotection >10) or blue (protection) **b** Mapping of regions affected by the p85α-Y685A mutation within p110δ. Colouring according to the legend. Roginolisib is shown to indicate the inhibitor binding site. The p85α regulatory subunit is coloured in green **c** Uptake plots of the p110δ peptide 1026-1044 showing deuterium incorporation at three deuterium labelling times for each of the studied conditions (WT p110α/p85α heterodimer in i) apo state; ii) Y685A heterodimer in apo state, iii) Y685A heterodimer in complex with roginolisib and iv) Y685A heterodimer when bound to idelalisib). Data are shown as percentage deuteration (mean +/- SD, n = 3). cs = charge state. **d** Comparison of H/D exchange rates in the p110δ peptides between apo and roginolisib (top) and between apo and idelalisib bound (bottom) for the activated p110δ/p85α-Y685A heterodimer. Data shown as in (**a**).

The differential mode of binding between roginolisib and idelalisib observed by both MD simulations on p110δ and experimental HDX-MS on p110δ/p85α heterodimer suggests that roginolisib may target the inactive state. To test this hypothesis, we generated a partially activated PI3Kδ heterodimer by inserting a mutation in the regulatory subunit that releases the p85α-cSH2 inhibitory contact^15,29^. The residue Y685 on p85α has been shown to mediate the inhibitory contact of the cSH2 domain with the elbow region connecting helices kα11 and kα12 of p110δ^15^. By replacing the tyrosine for alanine, the p85α-Y685A mutation results in enhanced catalytic activity of p110δ via the loss of cSH2-mediated inhibition. HDX-MS analysis confirmed that the p85α-Y685A mutation exposes the C-terminal kα11-kα12 helices of p110δ, together with the end of the substrate binding loop 927-940 lying just beneath the elbow and the closely-located 855-868 loop (Fig. 5a-c)^15^.

Binding of idelalisib to the p85α-Y685 PI3Kδ affected the same regions as in the WT heterodimer, indicating a similar binding mode between the activated and inhibited states (Figs. 4a, 5d). The situation differs for roginolisib, where protection of the kα12 helix (1022–1044) was only seen for the WT enzyme (Figs. 4a, 5d). This suggests that roginolisib, in contrast to idelalisib, makes additional contacts with the C-terminal region of the inactive kinase, which are lost upon activation of PI3Kδ. As both membrane binding and the release of p85α-cSH2 contact led to conformational rearrangements of the catalytic domain, these experiments suggest that roginolisib preferentially targets the inactive enzyme and, by limiting kα12 conformational flexibility, prevents its activation.

### Roginolisib’s binding mode involving kα12 interactions is selective for the delta isoform

Despite their conserved structures, sequence variations between PI3Ks present opportunities for isoform-selective targeting by pharmacological agents. Within the C-terminal kα12 helix, the two key residues identified above, Lys1040 and Arg1043, are unique to the δ and β isoforms (Supplementary Fig. 7a). Given that these residues appear to be involved in roginolisib’s binding to the kinase, a mechanistic rationale that explains the selectivity of the inhibitor towards the delta isoform is provided. A change in the C-terminal residues would be expected to disrupt the interactions identified to stabilise the roginolisib-induced kα12 conformation. Furthermore, the p85α cSH2 domain does not inhibit p110α^15^, and also the p101 regulatory subunit has no interactions with the C-terminus of the γ isoform^32^. Given the absence of Lys1040 and Arg1043 in the α and γ isoforms and the lack of the cSH2 type inhibition, roginolisib is predicted to be selective for p110δ, and to a lesser extent for the β isoform. This is consistent with in vitro IC50 measurements^19^, demonstrating over 500- and 1,000-fold selectivity against the α and γ isoforms, respectively, with comparatively low selectivity against the β isoform (Supplementary Fig. 7b).

### Idelalisib and roginolisib have a limited effect on PI3Kδ affinity for membrane binding upon stimulation

HDX-MS analysis has shown that the kα1-kα2 loop conformation is altered upon binding of either roginolisib or idelalisib in solution, raising the question of whether inhibitor binding could modulate membrane interaction. To understand if the presence of inhibitors affects PI3Kδ membrane interaction, we examined the binding of PI3Kδ to lipid membranes in presence of idelalisib and roginolisib using protein-lipid Förster resonance energy transfer (FRET) assays^15^. To phosphorylate their substrate, PI3Ks must be at the membrane. Affinity with membranes is mostly controlled by the regulatory p85 subunit for class IA PI3Ks. The p110 catalytic subunit makes direct contacts with the membrane involving the C-terminal catalytic domain. In the inhibited state, access to membrane of the p110δ subunit is blocked by the interaction of the cSH2 domain of p85α with the hinge between helices kα11 and kα12. Engagement of the cSH2 with phosphorylated tyrosines present on the cytosolic side of RTKs both disrupts the inhibitory contact with the p110δ catalytic subunit and positions the enzyme in proximity to the plasma membrane. As non-stimulated PI3Kδ has only a low affinity for membranes, we measured binding affinities in presence of an excess of a PDGFR phosphopeptide (pY), mimicking receptor tyrosine kinase engagement at the membrane. When testing on vesicles mimicking plasma membrane composition, neither roginolisib nor idelalisib impacted membrane interaction (Supplementary Fig. S8). This shows that the change in conformations of membrane-interacting kα1-kα2 and kα12 helices has only a limited effect on lipid interactions, at least for the pY-stimulated PI3Kδ heterodimer.

### The insertion of kα12 in the membrane primes p110δ for catalysis

**Fig. 6:**
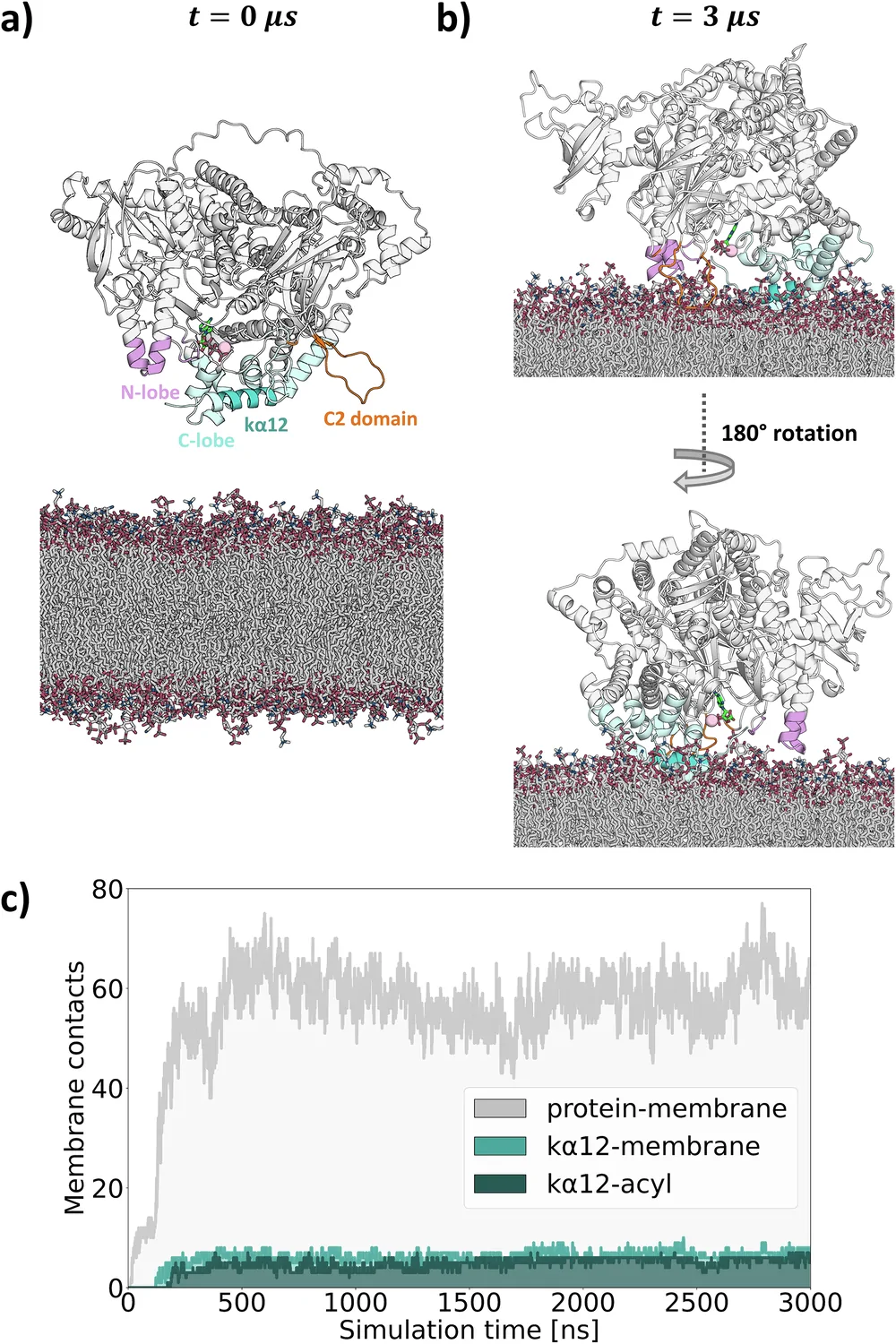
MD simulations of protein-membrane association reveal key domains involved in membrane-bound/active conformation of PI3Kδ. Regions forming contact with membrane are coloured (C2 domain: orange, N-lobe: purple, C-lobe: light blue). kα12 is highlighted in teal. Membrane lipids are displayed in licorice with light grey coloured tails and dark grey phosphorus atoms. **a** Molecular graphic of initial simulation frame. The distance between protein and membrane is 2 nm and the structure is oriented in a way in which activation is suggested^15,30,31,44^. **b** Molecular graphics of final simulation frame at 3 μs show active PI3Kδ conformation upon membrane binding. Upper graphic shows deep insertion of loop of C2 domain which includes 9 basic residues. Lower graphic shows deep insertion of kα12 helix of C-lobe. **c** Number of contacts formed by entire protein (grey) and kα12 (teal) over time depicts association process and membrane embedding of kα12 helix at 190 ns. The first contact is formed at ~20 ns by the C2 domain loop, followed by the insertion of ~13 residues into the membrane (grey) in the first ~120 ns.

**Fig. 7:**
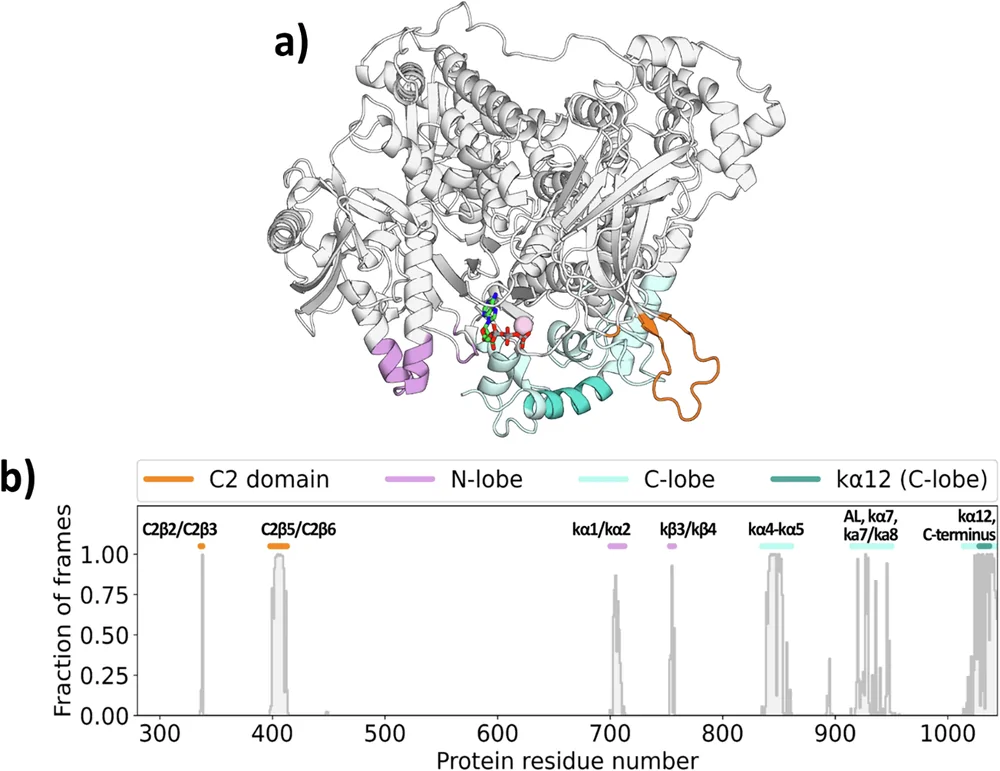
Regions forming contact to membrane during activation in MD simulations. **a** Molecular graphics of ATP⋅Mg^2+^-bound PI3Kδ with regions forming contact to membrane highlighted: C2 domain (orange), N-lobe (purple), C-lobe (light blue). kα12 is highlighted in dark teal. **b** Protein membrane contact map strongly agrees with regions to be in contact with membrane discovered through HDX experiments^15^. A “/“ separating two secondary structure elements stands for the hinge between those, whereas “-“ stands for both of them including the hinge between. “AL” is the Activation Loop. The map reveals three major regions forming large contacts with membrane: the large loop between C2β5 and C2β6, parts of the helices kα4 and kα5, and kα12 plus the C-terminus.

The kα12 helix of PI3Ks has previously been implicated in membrane binding (Supplementary Fig. 9)^15,31,32,35^. To further examine its role in the membrane targeting of PI3Kδ, we performed MD simulations of ATP-bound p110δ in the presence of PI(4,5)P2-containing membranes with a lipid composition approximating that of the leukocytic plasma membrane^36^. Initially with its active site oriented towards the membrane and at a distance of 2 nm from the nearest lipid (Fig. 6a), p110δ formed spontaneous membrane association, with over 60 protein residues brought into contact with membrane lipids in the first 500 ns of one simulation replicate (Fig. 6b and c). The first contacts were made by a loop region between β-sheet strands β5 and β6 of the C2 domain. The nine basic residues on the C2 β5/β6 loop established persistent interactions with negatively charged lipid headgroups (Fig. 6b) within ~50 ns of simulation time. Subsequent membrane contacts of the C-terminal kα12 helix, initiated by its Lys1028 interacting with PIP2, led to spontaneous insertion of kα12 into the membrane. The helix remained stably embedded in between lipid acyl chains through the duration of the 3 μs simulation (Fig. 6b and c). Its C-terminal end became more ordered in the membrane-bound state, which is consistent with the finding that in the class II PI3KC2α kα12 tends to be more structured in the active than in the inactive state^33^. Membrane insertion of kα12 was accompanied by formation of extensive protein-membrane interactions, and the interaction interface was maintained over the simulation timescale (Fig. 7). The stable interface of membrane contacts, formed by the N- and C-lobe, are in agreement with regions previously identified by HDX measurements^15^. The C2 domain membrane contacts were not observed by HDX, which may suggest that these interactions are less likely in presence of p85.

**Fig. 8:**
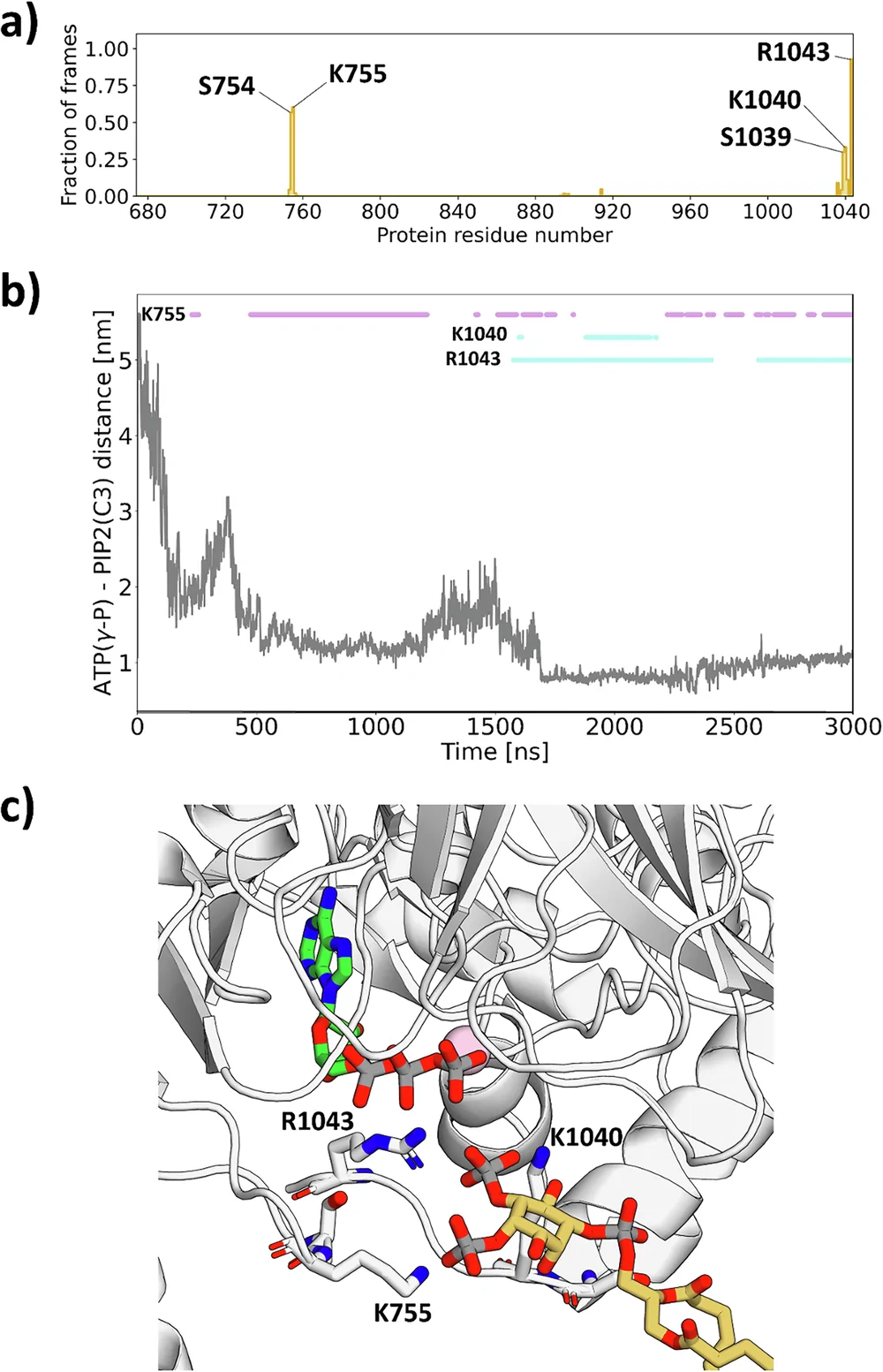
Basic residues of PI3Kδ interact with PIP2 in MD simulations, forming an active compound. **a** Protein-PIP2 contact map reveals residues directly stabilising the active compound. Key residues are the basic residues Lys1040 and Arg1043 of the C-terminus and Lys755 of the N-lobe. Residues within 4 Å of the PIP2 lipid were counted. In addition, the contact map reveals Ser1039 to be in close proximity to the lipid facilitating reports on autophosphorylation of the residue to impact kinase activity^45^ **b** Distance between the γ-phosphate of ATP and C3 of the inositol ring of the PIP2 lipid (grey) which is target for the phosphorylation depicts the insertion process of PIP2 into the active site. The first persistent contact is formed by Lys755 (purple) and both phosphate groups of PIP2 at ~500 ns. Stable interactions between PIP2 and the basic C-Terminus residues (light blue) follow at 1575 ns after guidance into the active site by Lys755 forming the phosphorylation competent active site. The top bars were created by adding a point at every frame in which the residues were within 4 Å of PIP2’s 5-phosphate. **c** Simulation snapshot of phosphorylation competent site at 3 us. ATP (green), PIP2 (yellow) and the active compound stabilizing residues are shown as sticks and the magnesium ion is shown as a bead (pink).

Notably, we observed spontaneous binding of a molecule of the lipid substrate, PIP2, into the kinase active site (Fig. 8). The PIP2 inositol ring interacted in close proximity with the γ-phosphate of the bound ATP, suggesting a configuration compatible with catalysis of the phosphate transfer reaction. Analyses of protein contacts stabilising the bound PIP2 revealed persistent interactions with Arg1043 and, to a lesser extent, Lys1040. To further examine the role of kα12 in lipid binding, we initiated additional simulations from the membrane-bound, PIP2-engaged p110δ configuration with kα12 deleted (Supplementary Fig. 10a). While p110δ remained bound to the membrane in all five replicates, likely mediated by interaction of the C2 domain (Supplementary Fig. 10b), the substrate PIP2 readily dissociated from the active site in three replicates (Supplementary Fig. 10c). In the two remaining replicates, the PIP2 molecule remained associated through coordination with the magnesium ion. No other membrane PIP2 lipids were observed to approach the p110δ active site during a total simulation time of 15 μs across the five replicates. The observed effect on substrate PIP2 binding may explain the earlier finding that truncation of kα12 drastically reduces lipid kinase activity^29^.

### Roginolisib leads to an efficient PIP3 decrease in double–refractory CLL patient cells

**Fig. 9:**
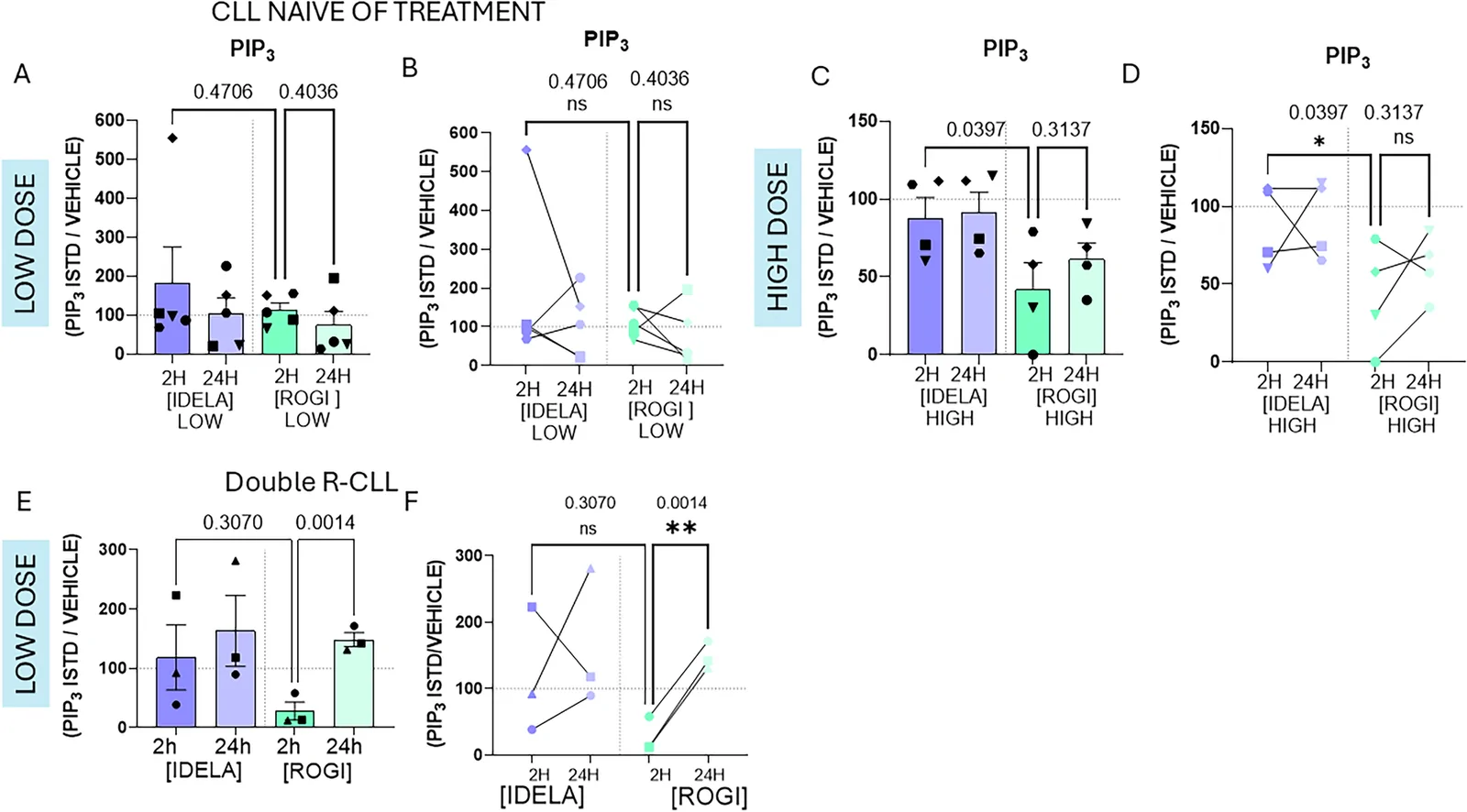
Lipidomic profiling of phosphoinositide molecular species in circulating tumor cells (CTCs) from CLL patients using LC-MS and TMS-derivatization for detection of PIP_3_. CTCs were isolated from CLL patient (naïve of treatment or double refractory) and treated with vehicle, idelalisib (IDELA—0.5µM-LOW or 2.5µM-HIGH) or roginolisib (ROGI-0.5 µM or 2.5 µM-HIGH) followed by lipid extraction using chloroform/methanol solvent with addition of internal standards (ISTD) for normalization and by derivatization with TMS-diazo-methane to enhance detection sensitivity. ISTD-normalized PIP3 levels in treated samples (idelalisib or roginolisib) were subsequently expressed as percentage relative to ISTD-normalized vehicle-treated controls. The dotted line represents values of 100% of the vehicle-treated control. Data are presented as % of VEHICLE at each time point. Each symbol represents a patient, to analyse time course evolution of each of them. **a**, **c**, **e** Mean ± SEM. **b**, **d**, **f** Evolution of PIP3 level in each patient. ANOVA one-way test; *, *p* < 0.05. *p*-values are shown. Data are shown as individual patient values, with each symbol representing a single patient (**a**, **b**: *n* = 5; **c**, **d**: *n* = 4; **e**, **f**: *n* = 3). Bars indicate the mean ± SEM. Statistical significance was assessed by using a one-way repeated-measures ANOVA with Geisser-Greenhouse correction, followed by Fisher’s LSD multiple-comparisons test (**a**, **b**); by using a one-way repeated-measures ANOVA mixed-effects model (REML), with Geisser-Greenhouse correction, followed by Fisher’s LSD multiple comparisons test (**c**, **d**); by using a one-way repeated-measures ANOVA, followed by Fisher’s LSD post hoc test without correction for multiple comparisons (**e**, **f**). Exact *p* values are listed in Supplementary Table 3.

The activity of roginolisib on CLL patient samples, as measured by the suppression of distal markers such as phosphorylation of AKT, ERK, FOXO1 and GSK3α/β, has been reported to be comparable to that seen with idelalisib or duvelisib^37^. To investigate whether the differences in binding modes translate into changes in actual kinase activity, we utilized a mass spectrometry–based approach to quantify PIP3 in cell lysates from CLL patient samples treated ex vivo with roginolisib and idelalisib at two fixed doses and time points (Fig. 9). Samples were obtained from both treatment naïve patients and patients refractory to BTK- and BCL-2 inhibitors. The analysis revealed that roginolisib was more effective than idelalisib in decreasing PIP3 levels at the 2 h time point and at high dose (Fig. 9c,d). We observed heterogeneity of response in tested patients, in particular at low doses in idelalisib-treated circulating tumour cells. Idelalisib led to a heterogeneous response between patient cells, with one naïve and two double refractory patient cells presenting a more than 2-fold increased peak of PIP3 production compared to vehicle, possibly indicative of treatment resistance (Fig. 9a,e). Interestingly, all tested refractory patient cells had a decreased PIP3 level upon 2 h treatment with roginolisib, which was significantly lower compared to 24 h treatment (Fig. 9e), suggesting a differential feedback response to the two inhibitors. These findings suggest that stabilization of the inhibited form of PI3Kδ may lead to more consistent suppression of the kinase activity compared to inhibition of the active kinase at the plasma membrane.

## Discussion

Designing selective ATP-competitive lipid kinase inhibitors is a challenge based on the conserved ATP-binding pocket. Improved inhibitor selectivity can be achieved by exploiting binding sites and regulatory features that are unique to individual kinases^38,39^. Kinases continuously cycle between productive (active) and non-productive (inactive) states, and since the inactive state potentially can adopt a distinct conformation, targeting the inactive state of a kinase is an attractive strategy to discover selective inhibitors. The latter may also not compete with ATP for binding^40^. In protein kinases, this approach is widely used as exemplified by the so-called type II and type III inhibitors, which induce a distinct DFG-out conformation. Inhibitors bind in an additional hydrophobic pocket created by the  rearrangement, or to an allosteric pocket adjacent or remote to the ATP- binding site^41,42^. Lipid kinase inhibitor design is less advanced, and the first allosteric isoform-mutant selective PI3K inhibitors have only recently entered clinical trials^4^. In parallel with efforts to identify inhibitors, a PI3Kα-selective allosteric activator was recently described, which provides cardioprotection from ischaemia-reperfusion injury^43^. This activator enhances multiple steps of the PI3Kα catalytic cycle, highlighting the benefit of studying enzyme regulation to design selective drugs with non-conventional modes of action.

Despite the availability of several crystal structures, little is known about which activation states are targeted by the different PI3K inhibitors. In class I PI3Ks, a key regulatory element is the regulatory arch composed of the C-terminal helices kα10-kα11-kα12, which encircles the catalytic- and activation loops essential for kinase activity^16^. Activation of all class I PI3Ks requires a reorientation of the C-terminus of the kinase domain to bind to lipid membranes, where they are further activated by Ras to exert their function. For full activation, the regulatory arch needs to open, allowing the catalytic site to be in close proximity to the membrane bound lipid substrate^15^.

The C-terminal helix kα12 has been reported to have a crucial role in PI3K catalysis. It was initially studied in the class III PI3K, Vps34, where the equivalent region was shown to be auto-inhibitory in the absence of lipid substrate but would bind to the cell membrane and facilitate kinase activation^31,44^. Based on HDX-MS experiments and kinase activity measurements, a similar mechanism of membrane binding and activation has since been suggested for the class I PI3Ks^15,29,32^. Truncation of kα12 completely removes all lipid kinase activity^29^. The importance of kα12 for catalysis is further highlighted by the gain or loss of PI3K function associated with mutations in the C-terminal region^45^. Autophosphorylation of p110δ Ser1039 was also shown to downregulate PI3Kδ lipid kinase activity^46^. In available co-crystal structures of the β and γ PI3K isoforms^29,35,47^, the C-terminus adopts a bent conformation with a hinge between the C-terminal helices kα11 and kα12. The conformation where the C-terminal helix kα12 covers the catalytic as well as the activation loop is considered the inactive state of the kinase^29,48^. A similar C-terminal helix configuration has been predicted by AlphaFold^28^ for PI3Kδ, in which the ∼18 C-terminal residues have been unresolved in experimental co-crystal structures of the protein.

We have used X-ray co-crystal structure data, molecular dynamics simulations, HDX-MS studies and PIP3 formation quantification in CLL patient samples to shed light on the mode of action of the next generation PI3Kδ-selective inhibitor roginolisib. Notably, major differences with the clinically approved first generation PI3Kδ inhibitor idelalisib were identified that may contribute to the improved tolerability of roginolisib in patients^49^. We found that roginolisib binding to PI3Kδ shows several differences compared to idelalisib. Roginolisib does not occupy the specificity pocket in PI3Kδ and the orientation of the side chains of Asp911 of the DFG motif and Asp897 are altered compared to idelalisib. Although these differences demonstrate a differentiated binding mode of roginolisib, they do not explain its selectivity nor its potency in the presence of high ATP concentrations. Because of the absence of electronic density for the catalytically important kα12 helix in the PI3Kδ crystal structures, we turned to MD simulations and HDX-MS experiments to investigate how these changes may affect its conformation.

MD simulations on the full-length p110δ AlphaFold model in complex with roginolisib showed that the two acidic residues Asp911 and Asp897 can interact with Lys1040 and Arg1043 of helix kα12, forming an electrostatic stabilisation contact. This results in a stabilization of the C-terminus in a bent conformation with the kα12 helix in a position that covers the active site, likely indicative of the PI3Kδ inactive state. The observed unfolding of the C-terminal half of kα12 provides the required flexibility for these interactions to occur in a similar manner to the reported stabilisation of the inactive conformation of PI3KC2α^33^. By contrast, such conformational stabilization of kα12 was not seen for the MD simulations done for the p110δ-idelalisib complex, which favours an opened regulatory arch that is more in accordance with a partially activated state^14^. The SPR data show fast on/off kinetics for roginolisib binding, suggesting that the ATP pocket in the inactive state remains accessible for ATP binding. However, the fact that ATP binding requires interaction of Lys1040 and Arg1043 with the gamma-phosphate of ATP likely attenuates the ATP affinity for roginolisib bound PI3Kδ. Occupation of the ATP-binding pocket combined with the electrostatic stabilisation of residues critical for nucleotide binding may thus explain why roginolisib maintains its potency at high ATP concentration^19^. The residues Lys1040 and Arg1043 are unique to the δ and β isoforms and therefore also explain the selectivity of roginolisib for p110δ, and to a lesser extent for the β isoform.

The stabilisation of helix kα12 by roginolisib was confirmed experimentally by HDX-MS. Comparison of the PI3Kδ H/D exchange profiles in absence and presence of either roginolisib or idelalisib showed that binding of each ligand has minimal impact on the global enzyme conformation. The hinge region of p110δ was protected by both molecules, as expected for inhibitors binding to the ATP-binding pocket. Both ligands also triggered conformational rearrangements in helices kα1-kα2 of the kinase domain, a region previously shown to mediate interactions with the p85α-cSH2 domain and membranes^15^. This change does not impact on membrane interaction of phosphopeptide-stimulated PI3Kδ, further supporting the inhibitor's mechanism of action affecting primarily the enzyme’s catalytic cycle. Protection from H/D exchange of helix kα12 was only seen when in complex with roginolisib. This protection indicates a stabilisation of the helix, owing to direct contacts with active site residues. It also supports a potential positioning of the helix in a bent inward-facing orientation, thus blocking the access to the active site. The observed protection of the helix kα12 is lost upon removal of the p85α-cSH2 inhibitory interaction in the p85α-Y685A mutant. These findings combined with the absence of exposure of the kα11-kα12 elbow region upon roginolisib binding support a mechanism where the binding of the cSH2 domain of p85α to p110δ is important for stabilizing the roginolisib bound complex. Since the PI3Kα and -γ lack the autoinhibitory cSH2 interaction, this mechanism further supports the high selectivity of roginolisib over these isoforms.

To further explore the conformational changes of kα12 upon membrane binding, MD simulations of ATP-bound p110δ in the presence of PIP2-containing membranes were performed. These simulations confirmed that kα12 readily and stably inserts into the membrane, likely facilitated by the observed increased ordering of the helix structure. Furthermore, spontaneous binding of PIP2 in the active site was observed. Interestingly PIP2 binding was stabilised through interactions with Arg1043 and Lys1040, the same residues involved in stabilising the roginolisb bound inactive kinase conformation. Removal of kα12 resulted in dissociation of PIP2 from the active site.

The combined MD simulations and HDX-MS experiments point toward roginolisib having a conformation-selective inhibitory mechanism, targeting specifically the inhibited enzyme and thereby hindering binding of both ATP and PIP2. Idelalisib on the other hand does not discriminate between active and inactive states since no bending of the C-terminal region was observed by MD simulations with idelalisib and H/D exchange profiles remained similar for WT and activated PI3Kδ. The more robust inhibition of PIP3 formation in ex vivo treated CLL patient samples observed for roginolisib compared to idelalisib may further suggest that selective targeting of the inhibited enzyme is more efficacious for attenuating pathological PI3Kδ signalling. Furthermore, roginolisib is highly efficient to decrease PIP3 levels in double-refractory patients that are resistant to the current treatment consisting of first-line ibrutinib and second-line ibrutinib plus venetoclax. Idelalisib does not work as well in double refractory patients compared to naïve patients. Increased accumulation of PIP3 has also been observed in post-idelalisib therapy CLL cells from patients and was explained by increased recruitment of PI3Kδ and/or PI3Kβ to the B cell receptor (BCR) complex^50^. Whether roginolisib binding affects this recruitment is a topic of current investigation.

In summary, high selectivity is a prerequisite for designing safe PI3K inhibitors. The first generation of PI3Kδ inhibitors aimed to achieve selectivity and potency towards p110δ by exploiting conformational flexibility of the enzyme in the ATP-binding pocket and inducing the formation of the specificity pocket^21^. Our work shows that roginolisib, binding in the ATP pocket but not interacting with the specificity pocket, inhibits the enzyme by stabilizing an inactive conformation through interactions with basic residues at the C-terminal end of kα12. This locks the p110δ-kα12 helix in an inactive conformation, an effect which was observed by investigating the protein dynamic effects of inhibitor binding on PI3Kδ. This work demonstrates that roginolisib is a conformation-selective inhibitor, and this inhibition mode for PI3Kδ not only results in high isoform selectivity and improved efficacy but also contributes to an improved safety profile in cancer patients. Ongoing studies aim to determine whether this stabilization leads to further distinct biological effects.

## Methods

### Ethics statement

Research using human sample follows the European Union and French national reglementary and ethical regulations. ANSM competent authority and national ethical committee CPP16 respectively approved the studies (ANSM, n° 1551668A-11; N° CPP16-004a). Those studies were registered with Clinical-Trials.gov (NCT02824159 and NCT02005471). Informed written consent from each patient was received and peripheral blood samples from untreated and double refractory CLL patients were obtained from the Hematology Department of the IUCT-Oncopole, Toulouse, France. The cells were stored and referenced in the Inserm cell bank in an anonymized manner. Inserm cell bank has been registered with the Ministry of Higher Education and Research (DC- 2013- 1903) after being approved by an ethics committee. Clinical and Biological annotations of the samples have been reported to the Comité National Informatique et Liberté.

### Chemicals and reagents

Liquid chromatography solvent Acetonitrile HPLC-grade (ACN) and Trimethylsilyl diazomethane 2 M in Hexane (TMSD) were purchased from Sigma Aldrich. Acetic acid (AA) was from Fluka. Formic acid (FA), Methanol HPLC-grade (MeOH) and Chloroform were purchased from Sigma Aldrich. Synthetic lipid standards. 1-heptadecanoyl-2-(5Z,8Z,11Z,14Z-eicosatetraenoyl)-sn-glycero-3-phospho-(1’-myo-inositol-3’,5’-bisphosphate) (PIP2 17:0-20:4) and 1-heptadecanoyl-2-(5Z,8Z,11Z,14Z-eicosatetraenoyl)-sn-glycero-3-phospho-(1’-myo-inositol-4’-phosphate) (PIP 17:0-20:4) were purchased from Avanti Polar Lipids.

### Protein crystallography

The complex of human p110δ (amino acids 1-1044 preceded by an N-terminal cleavable HIS-tag) and bovine p85 (amino acids 431-600, no tag) was co-expressed in Sf9 cells at 26 °C from two separate viruses. Cells were harvested 48 h after infection and the p110δ/p85 complex was purified by a three-step chromatographic procedure at 4 °C. Briefly, the pellet from a 5 L expression culture was lysed using an Ultra-Turrax and the lysate was centrifuged for 30 minutes at 75,000 × g. Protein was captured in batch mode by adding 12 mL of Ni Sepharose HP beads to the supernatant and incubating the suspension for 2 h. Beads were poured in an XK16/20 column and bound protein was recovered by imidazole elution. The HIS-tag was removed by overnight incubation with TEV-protease and protein was passed over the Ni-column used for the initial capture step. Protein complex in the flow-through was collected and purified to homogeneity on a Superdex200 26/60 column equilibrated in 50 mM Tris/HCl, 150 mM NaCl and 1.5 mM DTT. For crystallization, protein at 5 mg/mL concentration was incubated with either roginolisib or idelalisib dissolved at 10 mM in DMSO and crystallized by vapor diffusion in hanging drops. Equal volumes (1 µL:1 µL) of protein and reservoir solution (12-14% PEG 6000, 100 mM MES/NaOH at pH=5.75) were mixed and equilibrated against 1 mL of reservoir solution containing 5 mM DTT at 20 °C. Protein crystals that grew within 2–3 days were optimized for diffraction by using the Free Mounting System™^51^ and frozen for data collection. at a temperature of 100 K at the Swiss Light Source (SLS, Villingen, Switzerland). The crystals belong to space group P2_1_2_1_2_1_. Data were processed using the programs autoPROC^52,53^ and AIMLESS^54^. The structures were solved by molecular replacement using Phaser^55^ and a published human PI3Kdelta crystal structure (PDB ID: 6PYR). Manual model rebuilding was performed in COOT^56^ with alternated rounds of structure refinement in BUSTER (version 2.11.8: Global Phasing Ltd., RRID: SCR_015653).

### Surface plasmon resonance measurement

#### Protein and compounds

Biotinylated human PI3Kδ/PI3KR1 (11-403-20 N) was purchased from Carna Biosciences (Japan). Roginolisib was provided by iOnctura SA and idelalisib was obtained from the Oncolines reference inhibitor collection. Compounds were dissolved in DMSO at 10 mM stock solution.

#### Kinase immobilization

SPR experiments were performed on a Biacore 1S+ instrument using a Series S Sensor Chip SA (Cytiva). An aliquot of biotinylated kinase protein was thawed, diluted to 64.6 μg/mL in pre-cooled Biacore buffer (50 mM Tris pH 7.5, 0.05% Tween 20, 150 mM NaCl and 5 mM MgCl2) and immobilized with the biotin-tag on a SA chip. First, the streptavidin on the sensor chip was washed three times with a washing solution (1 M NaCl, 50 mM NaOH) for a period of 60 s with a flow rate of 10 μL/min. Then, biotinylated protein was captured on the chip with a targeted immobilization level of 8000 RU. Remaining streptavidin was blocked with 10 μg/mL biocytin injections to circumvent a-specific binding of compounds to the streptavidin-bound surface. To obtain a stable surface with a functional protein, the immobilization procedure was performed at 4 °C. Channel 1 was used as a reference channel to correct for buffer effects, other channels were immobilized as described above. The immobilization level for PIK3CD/PIK3R1 was 7526 RU. After immobilization, the system was buffered with Biacore buffer containing 1% DMSO. After three times priming, a pre-run was performed for a minimum period of 30 min at a flow rate of 30 μL/min to obtain a stable surface.

#### Single-cycle kinetics

Binding kinetics were assessed using a single-cycle kinetics (SCK) format at 22 °C. To ensure signal stability, five startup cycles consisting of consecutive buffer injections were performed prior to compound analysis. All compound dilutions were prepared in running buffer containing 1% DMSO to avoid bulk refractive index shifts arising from DMSO concentration differences. Roginolisib was tested at concentrations of 1, 3.16, 10, 31.6, and 100 nM, whereas idelalisib was tested at 0.316, 1, 3.16, 10, and 31.6 nM. Each concentration series was injected sequentially with a contact time of 100 s at a flow rate of 30 μL/min, followed by a 1,200 s dissociation phase. A blank SCK run consisting of five buffer injections under identical experimental conditions was performed before each compound series and used for double-referencing correction.

#### Data analysis

The compound curves were analysed with the Biacore Evaluation Software by using the method of double referencing. In the first step, the reference channel was subtracted from the channel containing immobilized protein, and then the reference curve obtained with buffer injections was subtracted. The resulting curve was fitted with the 1:1 binding model.

According to GE Healthcare the working range of the Biacore 1S+ is:

Association rate constant, k_a_: up to 5 ×10^7 ^M^−1^s^−1^

Dissociation rate constant, k_d_: 10^−6^ – 6 s^−1^

This means that for compounds with a very slow dissociation rate (k_d_ <10^−6 ^s^−1^), no exact dissociation constants can be determined. In these cases, the Biacore software indicates an inability to fit. For compounds with such extremely slow-off behaviour, we report values with operators ( > and <) for k_d_ and K_D_, respectively^27^ (Supplementary Fig. 3c). This experiment was performed by Oncolines BV.

### Molecular dynamics simulations

Initial structures of full-length p110δ in complex with bound idelalisib (PDB ID: 4XE0^57^) or roginolisib or ATP were modelled using MODELLER^58^ based on the best-ranked p110δ AlphaFold model^28^ and structures of the respective protein-ligand X-ray co-crystals. The final structural model for the roginolisib-bound protein consisted of the crystal structure with unresolved termini, loops, and their flanking regions taken from the AlphaFold prediction (namely residues 1–22, 174–193, 221–243, 277–319, 396–418, 494–508, 839–857, 915–935, and 1007–1044). The same structural model was used for the ATP-bound protein, with the ATP binding pose taken from a crystal structure of the ATP-bound porcine p110γ isoform (PDB ID: 1E8X^35^) and the relative position of the coordinated magnesium ion taken from PDB ID: 1CSN^59^ to generate the full complex. In the case of the idelalisib-bound complex, the crystallographically resolved murine structure was replaced by the full AlphaFold model of the human protein upon structural alignment. The co-crystallised p85 iSH2 domain in the roginolisib-bound structure was included in two simulations of the complex in solution and removed in all other simulations. MD simulations were performed in Gromacs 2020^60^, using the AMBER99SB*-ILDN-q force field^61–64^ and CHARMM36m^65^ force fields, respectively, for simulations of the protein-ligand complex in solution and in the presence of membranes. Roginolisib and idelalisib were parameterised using the general Amber Force Field (GAFF)^66^. ATP in the membrane simulations was parameterised via CHARMM-GUI (https://charmm-gui.org/)^65,67,68^. For simulations of the kinase domain in solution, the protein-ligand complexes were solvated with TIP3P water^69^ and 150 mM NaCl. The resulting dodecahedron simulation boxes were of size ∼2200 nm^3^ and contained ∼215,000 atoms. Particle-mesh Ewald electrostatics were used^70^. Upon energy minimization, five stages of equilibration were performed, in which the positional restraints on protein heavy atoms were successively decreased, first in an NVT ensemble (0.25 ns) and subsequently in an NPT ensemble (4 × 0.5 ns) using a Berendsen thermostat and barostat^71^. Production runs were performed in an NPT ensemble, with an integration time step of 2 fs. Five replicates were prepared per system. The duration of simulation replicates were 500 ns in the case of ATP-bound p110δ and between 2 µs (four out of five replicates) and 5 µs (one replicate) for the roginolisib- or idelalisib-bound protein. Temperature was maintained at 310 K using a Nosé-Hoover thermostat^72,73^, with the pressure maintained at 1 bar using an isotropic Parrinello-Rahman barostat^74^.

For protein-membrane simulations, the membrane lipid composition was based on a leucocytic plasma membrane lipid composition^36^ consisting of 15% dioleoylphosphatidylcholine (DOPC), 10% 1-palmitoyl-2-oleoyl-sn-glycero-3-phosphocholine (POPC), 15% dioleoylphosphatidyl-ethanolamine (DOPE), 15% 1-palmitoyl-2-oleoyl-sn-glycero-3-phosphoethanolamine (POPE), 35% cholesterol, 5% 1-palmitoyl-2-oleoyl-sn-glycero-3-phosphoserine (POPS), and 5% PIP2 for both leaflets. Membranes were constructed initially in a coarse-grained representation using the insane method^75^. Six independent replicates were prepared, each with a random distribution of lipid species and solvated with 150 mM of aqueous NaCl. Coarse-grained membrane systems were equilibrated for 200 ns and converted into atomistic representation using the CG2AT2 tool^76^. The ATP-bound p110δ structure was placed above the upper membrane leaflet with a minimum distance of 2 nm between protein and lipid atoms, and the height of the simulation box was increased to 20 nm. The final protein-membrane systems, containing ~350,000 atoms, were re-solvated with 150 mM of NaCl using TIP3P water^69^ and further equilibrated for 10 ns prior to 2 μs production simulations. The simulation replicate in which spontaneous membrane insertion of kα12 occurred was subsequently extended to a total duration of 3 μs. Based on the membrane-bound configuration at 2.4 μs, a separate set of five replicate simulations were initiated upon deletion of kα12 (residues 1025–1044) to generate an electrically neutral C-terminus, followed by re-solvation and equilibration before production simulations (Supplementary Fig. 10). Harmonic positional restraints were applied to non-hydrogen protein atoms during equilibration, with a force constant of 1000 kJ mol^−1^ nm^−2^. System temperature and pressure were maintained at 310 K and 1 bar, using the velocity-rescaling thermostat^77^ and a semi-isotropic Parrinello-Rahman barostat^74^ in the production phase. The integration time step was 2 fs. Long-range electrostatic interactions were treated using the smooth particle mesh Ewald method^70,78^. The LINCS algorithm was used to constrain covalent bonds involving hydrogen atoms^79^. Simulation trajectories were analysed through MDAnalysis 2.0^80^ in Python 3.6.

### Lipid vesicle preparation for membrane affinity assays

Dansyl-containing lipid vesicles mimicking plasma membrane were prepared with the following composition (all lipids were from Avanti lipids): 5 % Brain PI(4,5)P2 (#840046); 10 % Dansyl PS (#810225); 20 % Brain PS (#840032), 35 % DOPE (#850725); 15 % DOPC (#450375); 10 % Cholesterol (#700000); 5 % Sphingomyelin (#860061). Lipids were mixed in organic solvent before evaporation with nitrogen flow and desiccation under vacuum for 15 h at 22 °C. Lipids were resuspended in lipid buffer (20 mM Hepes pH 7.5, 100 mM KCl, 1 mM EGTA) at a concentration of 1 mg/ml. Vesicles were sonicated for 10 min, subjected to five freeze-thaw cycles and extruded 17 times through a 100 nm filter. Aliquots were flash-frozen in liquid nitrogen and stored at −80 °C.

### Protein-lipid FRET assays

Protein-lipid FRET assays were performed in 384-well plates keeping PI3Kδ, phosphopeptide and inhibitor concentrations constant while increasing the amounts of lipid vesicles. PI3Kδ (final 400 nM) were premixed with PDGFR phosphopeptide (pY, final concentration 5 µM) and inhibitors (final concentrations 10 µM) at a two-fold concentration in 9 µl in protein buffer (20 mM Tris pH 7.4, 150 mM NaCl, 2 mM DTT). Serial dilutions of liposomes were done in a final 9 μl volume in lipid buffer (20 mM Hepes pH 7.5, 100 mM KCl, 1 mM EGTA). Following 15 min incubation at 22 °C, 9 μl of lipid was added to 9 µ of protein and incubated for another 15 min at 22 °C. Plate was then read using a BMG Labtech plate reader using 280 nm excitation filter with 350 nm and 520 nm emission filters to measure tryptophan and Trp-Dansyl FRET emisissions respectively. The FRET signal shown in the figure has I-Io along the Y axis, where I is the intensity of the 520 nM signal and Io is the intensity of protein alone. The FRET signal at 520 nm shown in Supplementary Fig. 8 has I - I0 along the Y axis where I is the intensity at 520 nm in the presence of protein and I0 is the intensity of the sample in the absence of protein.

### Deuterium labelling experiments

HDX-MS experiments were performed at the UniGe Protein Biochemistry Platform (University of Geneva, Switzerland) following a well-established protocol with minimal modifications^76^. The Supplementary Table 2 and supplementary Data 1, 2 details all the reaction conditions (Supplementary Table 2) as well as all results (Supplementary Data 1, 2). For all conditions, full-length p110δ/p85α heterodimer was used, but only peptides of the p110δ catalytic subunit were studied and analysed. Mutation “K1040A” refers to mutation of p110δ-K1040A and “Y685A” refers to mutation of the regulatory subunit p85α-Y685A. A wild-type p85α and p110δ were used respectively for the K1040A and Y685A heterodimers. The following conditions were tested and compared: i) PI3Kδ-WT ii) PI3Kδ-K1040A, iii) PI3Kδ-Y685A, iv) PI3Kδ-WT + roginolisib, v) PI3Kδ-K1040A + roginolisib, vi) PI3Kδ-Y685A + roginolisib, vii) PI3Kδ-WT + idelalisib, viii) PI3Kδ-K1040A + idelalisib and ix) PI3Kδ-Y685A + idelalisib. Briefly, a first concentrated mix was prepared and preincubated for 5 min at the reaction temperature before initiating deuteration reaction.

The deuterium exchange reaction was initiated by adding deuterated buffer at the defined temperature to a final reaction volume. Reactions were carried-out for defined times and terminated by the sequential addition of of ice-cold quench buffer. Samples were immediately frozen in liquid nitrogen and stored at −80 °C for up to 2 weeks. Number of replicates for each conditions are described in Supplementary Table 2.

### Quantification of deuterium incorporation

To quantify deuterium uptake into the protein, samples were thawed and injected in a UPLC system immersed in ice with 0.1 % FA as liquid phase. Buffer delivery to all valves and columns was done with a dual pump (ThermoFisher Dual Pump F). The protein was digested via an immobilized Pepsin column (AffiPro AP-PC-006), and peptides were collected onto a Nucleodur 300-5 C18 pre-column filter (Macherey-Nagel). The trap was subsequently eluted, and peptides separated with a C18, 175 Å, 1.9 μm particle size Thermo Hypersil Gold Vanquish 100 ×2.1 mm column over a gradient of 8–30 % buffer C over 20 min at 150 µl/min (Buffer B: 0.1% formic acid; buffer C: 100% acetonitrile). Mass spectra were acquired on an Orbitrap Velos Pro (Thermo), for ions from 400 to 2200 m/z using an electrospray ionization source operated at 300 °C, 5 kV of ion spray voltage. Peptides were identified by data-dependent acquisition of a non-deuterated sample after MS/MS and data were analyzed by Mascot 2.6 using a database composed of purified proteins and known contaminants. Precursor mass tolerance was set to 10 ppm and fragment mass tolerance to 0.6 Da. Protein digestion was set as nonspecific. All peptides analysed are shown is Supplementary Data 1, 2. Deuterium incorporation levels were quantified using HD examiner version 3.4.2 software (Sierra Analytics), and quality of every peptide was checked manually. Results are presented as percentage of maximal deuteration compared to theoretical maximal deuteration. A change in deuteration level between two states is considered as significant if the sum of all changes across 3 timepoint (sum %D * sum #D) is > 5. All raw data and MS files are available in the PRIDE database (Project PXD071872).

### Sample preparation and PBMC isolation from CLL patients

All patients had chronic lymphocytic leukemia (CLL) and were of Caucasian ethnicity from France. Patients were recruited at the IUCT-Oncopole (Institut Universitaire du Cancer de Toulouse-Oncopole), Toulouse, France. A total of nine patients were included in the study. Five patients were treatment-naïve and received low-dose treatment (mean age 70.0 years, range 55–80; all male), four patients were treatment-naïve and received high-dose treatment (mean age 66.8 years, range 27–83; four males and one female), and three patients were double-refractory to venetoclax and ibrutinib (mean age 75.0 years, range 66–80; all male). Sex was self-reported by participants. No sex-based subgroup analysis was performed due to the limited sample size. Peripheral blood mononuclear cells (PBMCs) were isolated by density gradient centrifugation using Ficoll. PBMCs from CLL patients were seeded at 5 × 10^6^ cells/ml in culture medium (providing long-term viability). Circulating tumor cells from patients were treated with or without the vehicle (DMSO) or idelalisib or roginolisib (0.5 µM and 2.5 µM) for 2 or 24 h. The cells were collected and centrifuged at 800 g at 4 °C for 5 min; the culture medium was quickly aspirated and cells were resuspended in cold HCl 1 M (1 mL). The lysate was centrifuged for 5 min at 15,000 × *g* at 4 °C. The cell pellet was stored at −80 °C until required for PIP extraction. Lipids were extracted and derivatised using TMS-diazo-methane as described by Clark et al.^81^.

### Lipidomics extraction

Lipid extraction was performed using a modified MetaToul lipidomics workflow, with all samples kept on ice throughout the procedure. Cell pellets were resuspended in cold water and briefly sonicated (20% amplitude, 5 s). Internal standards consisting of phosphoinositide species (PIP, PIP₂, PIP₃) were added to all samples. In parallel, blank controls and brain-derived phosphoinositide standards were processed under identical conditions. A methanol/chloroform/HCl-based “kill mix” was added to initiate lipid extraction and quench biological activity. Samples were vortexed and subjected to biphasic partitioning by addition of chloroform and acid, followed by centrifugation (1500 × g, 5 min). The lower organic phase was carefully collected, avoiding the interphase. The organic fraction was subjected to sequential liquid–liquid wash steps using methanol/chloroform-based mixtures under acidified conditions to improve lipid recovery and remove contaminants. The purified lipid extract was then transferred to clean tubes for derivatization. Phosphoinositides were derivatized using TMS-diazomethane in hexane under controlled conditions. Reactions were performed at room temperature for 10 min and quenched with acetic acid. Extreme care should be taken when handling it. For this reason, methylation by TMSD has been carried out in a fume hood, with the use of adequate personal safety equipment. Following derivatization, samples underwent additional washing steps using methanol/chloroform/water mixtures to remove excess reagent and by-products. After each centrifugation step, the lower organic phase was recovered and pooled. The lower phase was collected, washed twice and dried under azote, then dissolved in 30 µL MeOH/H2O (2:1). The extract was then stored at −20 °C before LC-MS/MS analysis. Finally, lipid extracts were concentrated under a gentle stream of nitrogen, reconstituted in methanol/water, and sonicated prior to LC-MS analysis. Samples were injected at 10 µL and stored at −20 °C prior to analysis. Immediately before injection, samples were sonicated for 1 min to ensure homogeneity.

### PIP measurement by mass spectrometry

#### LC/GC-MS analysis of samples using Agilent 1290 Infinity instrument

High-performance liquid chromatography was performed using an Agilent 1290 Infinity (Agilent Technologies) equipped with an auto sampler, a binary pump and a column oven. The analytical column was an Acquity UPLC BEH300-C4 (100 ×1.0 mm, 1,7 μm) (Waters) maintained at 22 °C. The mobile phases consisted of Water, FA (99.9:0.1;v/v) (A) and ACN, FA (99.9:0.1, v/v) (B). The gradient was as follows: 45% B at 0 min, 100% B at 5 min, 100% B at 8 min, 45% B at 9 min, 45% B at 10 min. The flow rate was 0.1 mL/min. The auto sampler was set at 5 °C and the injection volume was 10 µL. The HPLC system was coupled on-line to an Agilent 6460 triple quadrupole MS (Agilent Technologies) equipped with electrospray ionization source. Electrospray ionization (ESI) was performed in positive ion mode. After optimization, the source parameters used were as follows: source temperature was set at 250 °C, nebulizer gas (nitrogen) flow rate was 8 L/min, sheath gas temperature was 150 °C, sheath gas (nitrogen) flow rate was 3 L/min and the spray voltage was adjusted to +4000 V. The collision energy optimums for derived PIP (36:2) and derived PIP2 (36:2) were 30 eV.

#### LC/GC-MS analysis of PIP lipids using Shimadzu instrument

PIPs were analyzed using high-performance liquid chromatography coupled to tandem mass spectrometry (HPLC-MS/MS). Lipid separation was performed on a ACQUITY UPLC Protein BEH C4 (300 A, 1.7 µm; 1X100mm, ref 186005590 Waters) using a Nexera Mikros HPLC system (Shimadzu, Kyoto, Japan) coupled to an LCMS-8060 triple quadrupole mass spectrometer (Shimadzu, Kyoto, Japan) equipped with an electrospray ionization (ESI) source operating in positive ion mode. The mobile phases consisted of 5 mM Formate Ammonium in water (mobile phase A) and MeOH Formate Ammonium in methanol (mobile phase B). The gradient was as follows: 75% B for 1 min, 100% B at 3 min, 100% B at 5 min, 75% B at 5.1 min, 75% B at 8 min. The flow rate was set to 0.1 mL/min, the column temperature was maintained at 40 °C, and the autosampler was kept at 15 °C. The injection volume was 1 μL. Mass spectral analysis was performed on LC-triple quadrupole mass spectrometer (LC-vcQQQ 6460 Agilent and LCMS-8060 Shimadzu) equipped with positive mode electrospray ionisation as described by Thibault et al.^82^. Analyses were performed in Selected Reaction Monitoring detection mode (SRM) using nitrogen as collision gas. Finally, peak detection, integration and quantitative analysis were performed using MassHunter QqQ Quantitative analysis software (Agilent Technologies VersionB.05.00) and Microsoft Excel software. Data were processed using QqQ Quantitative (vB.05.00) and Qualitative analysis software (vB.04.00). Internal standards (PI(5)P[17:0-20:4], #LM1902-1EA; PI(4,5)P₂[17:0-20:4], #LM1904-1EA; PI(3,4,5)P₃ [17:0-20:4], #LM1906-1EA) and running control (PI4P Brain; #840045; PI(4,5)P2 Brain, #840046; PI(3,4,5)P3[18:0-20:4], #850166) were from Avanti Research. The mixture of synthetic PIP/PIP2/PIP3 internal standards was added during the preparation of the samples. The stock solution of internal standards (ISTD) was prepared at 10 ng/µL and diluted to a working concentration of 1 ng/µL. A total of 20 µL of this mixture was added to each sample, corresponding to 20 ng of each internal standard. Running controls with ISTD were separately used as positive controls. All lipid species were normalized to internal standards (lipid area / ISTD area), providing relative quantification between samples. PIP3 levels in treated samples (idelalisib or roginolisib) were subsequently expressed as percentage relative to vehicle-treated controls.

#### Data annotation, validation and analysis procedure

All samples are anonymized then numbered separately in the separate sample preparation. All analyses were performed under standardized conditions, and instrument performance was routinely monitored using quality control samples to ensure analytical consistency across runs. Blank samples were analyzed to assess background signal and confirmed the absence of interfering peaks. Peak detection, integration, and quantitative analysis were performed within the corresponding vendor software environments. All peaks were manually inspected to ensure correct integration and consistent peak assignment across samples. Data were exported and further processed using Microsoft Excel.

### Reporting summary

Further information on research design is available in the Nature Portfolio Reporting Summary linked to this article.

## Supplementary information

Supplementary Information

Description of Additional Supplementary Files

Supplementary Movie 1

Supplementary Movie 2

Supplementary Movie 3

Supplementary Data 1

Supplementary Data 2

Reporting Summary

Transparent Peer Review file

## Source data

Source Data File

## Data Availability

Source data are provided with this paper in the supplementary material, including for the HDX-MS experiments (Supplementary Table 2, Supplementary Data 1, 2). Raw files related to HDX-MS experiments have been deposited to the PRIDE database (PXD071872). The X-ray structures have been deposited in the protein data bank (PDB) under IDs 9T07 https://doi.org/10.2210/pdb9T07/pdb (roginolisib) and 9T1P https://doi.org/10.2210/pdb9T1P/pdb (IOA-288). Full MD simulation input and coordinate files have been deposited at Zenodo:https://doi.org/10.5281/zenodo.17911609. Any other relevant data are available from the corresponding authors upon request. Source data are provided with this paper.
