# Supplementary material for: PI3Kδ is selectively inhibited by roginolisib by stabilizing the C-terminal helix kα12
Source: Nat Commun. 2026 Oct 9;17:10220. doi: 10.1038/s41467-026-77424-0 (PMC13652804; doi:10.1038/s41467-026-77424-0)
Supplement: Supplementary file 1 [file 41467_2026_77424_MOESM1_ESM.pdf]

Supplementary Information

PI3Kδ is selectively inhibited by roginolisib through stabilizing of the C-terminal helix kα12

Oscar Vadas[1,*], Simon Tiede[2,*], Maria Chaouki[3,*], Laura Tesmer[2], Shanlin Rao[2], Rémy Visentin[1], Ulrich Grädler[4], Martin Augustin[5], Rainer Kiefersauer[5], Carola Gößer[5], Anne Quillet-Mary[3], Loïc Ysebaert[6], Julie Guillermet-Guibert[3], Lars van der Veen[7], Giusy Di Conza[7] and Gerhard Hummer[2,#]

[1] Protein Biochemistry Platform, Faculty of Medicine, University of Geneva, Geneva, Switzerland

[2] Max Planck Institute for Biophysics, Frankfurt am Main, Germany

[3] Centre de Recherches en Cancérologie de Toulouse, Inserm, CNRS, Université de Toulouse, Labex Toucan, Toulouse, France

[4] Merck Healthcare KGaA, Darmstadt, Germany

[5] Proteros biostructures GmbH, Planegg-Martinsried, Germany

[6] Service d'Hématologie, IUCT-Oncopole, Toulouse, France

[7] iOnctura SA, Geneva, Switzerland

[*] equal contribution

[#] Correspondence : gerhard.hummer@biophys.mpg.de (G.H.)

SUPPLEMENTARY INFORMATION

Content of SI :

Supplementary Figures 1-10

Supplementary Tables 1, 2, 3

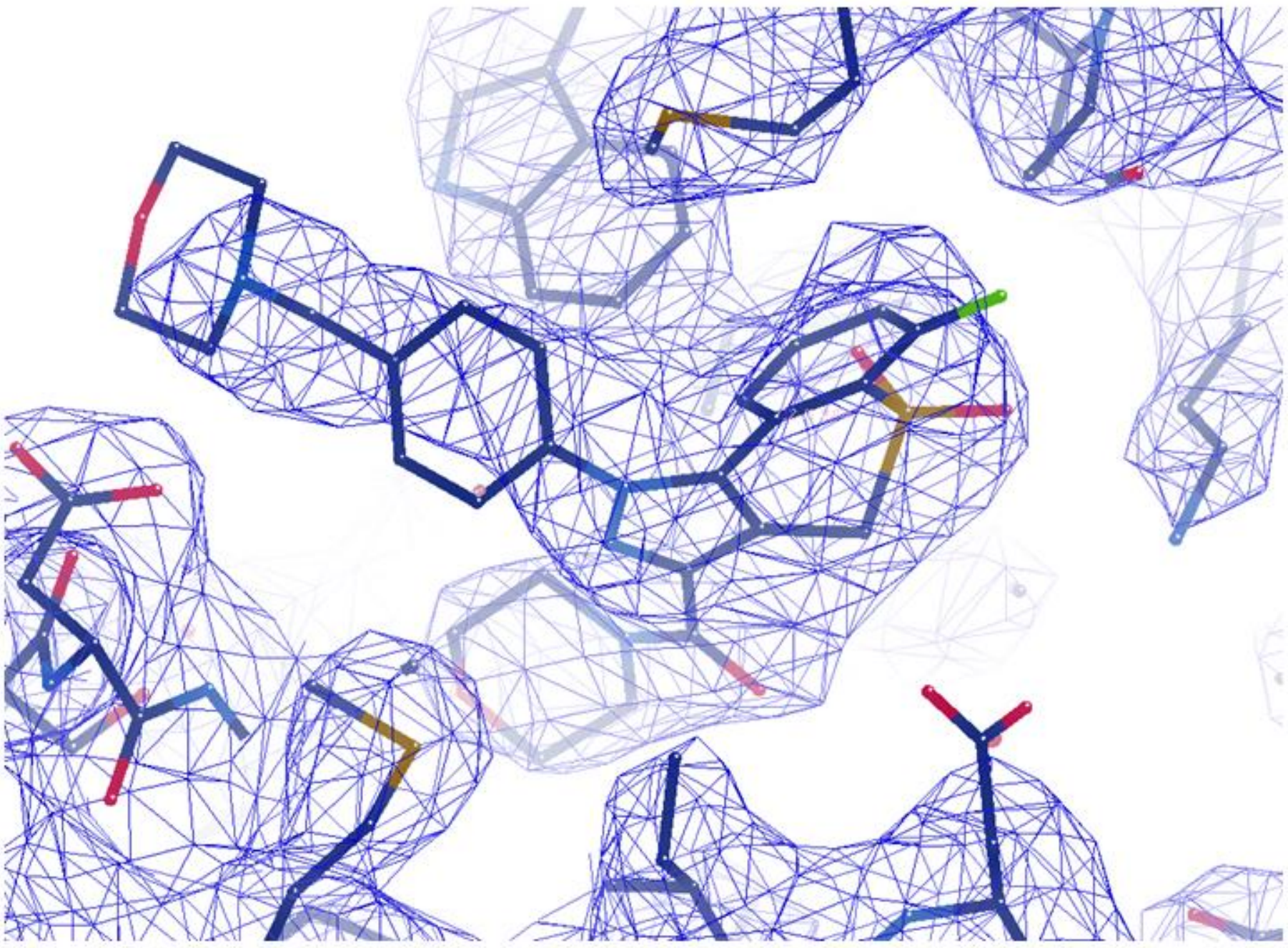

Supplementary Figure 1

Crystal structure of human PI3Kδ in complex with roginolisib (cyan) solved at 2.75 Å resolution (PDB-ID: 9T07). The 2mFo-Dfc electron density map (blue) contoured at 1.0 σ revealed an unambiguous binding mode identification of roginolisib and IOA-288, respectively.

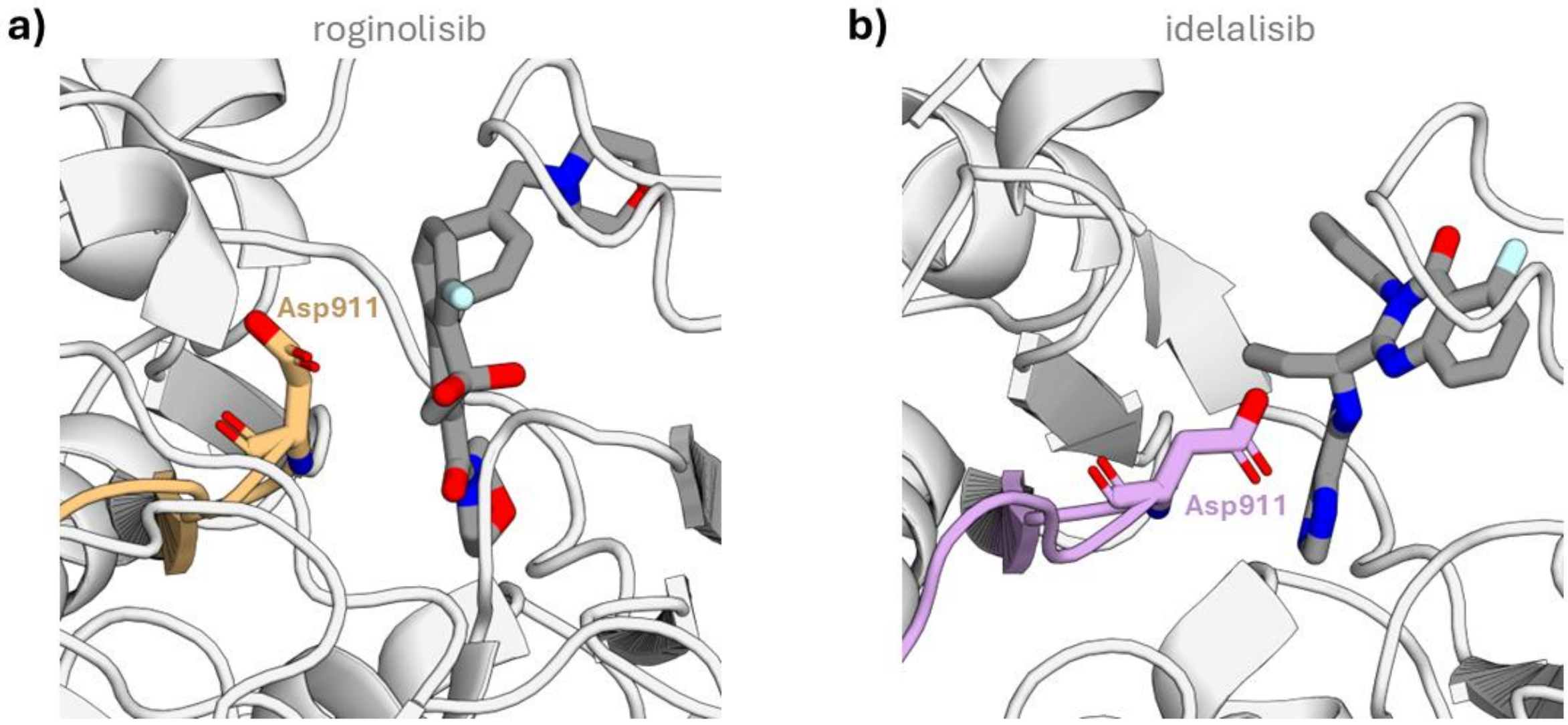

Supplementary Figure 2

D911 of the DFG kinase motif adopts alternative conformations in roginolisib- and idelalisib-bound PI3Kδ. **a** Representative snapshot depicting D911 (light orange sticks) being sterically pushed by roginolisib into a DFG-in like conformation. **b** D911 (light purple sticks) closer to a DFG-out conformation in the presence of idelalisib.

a)

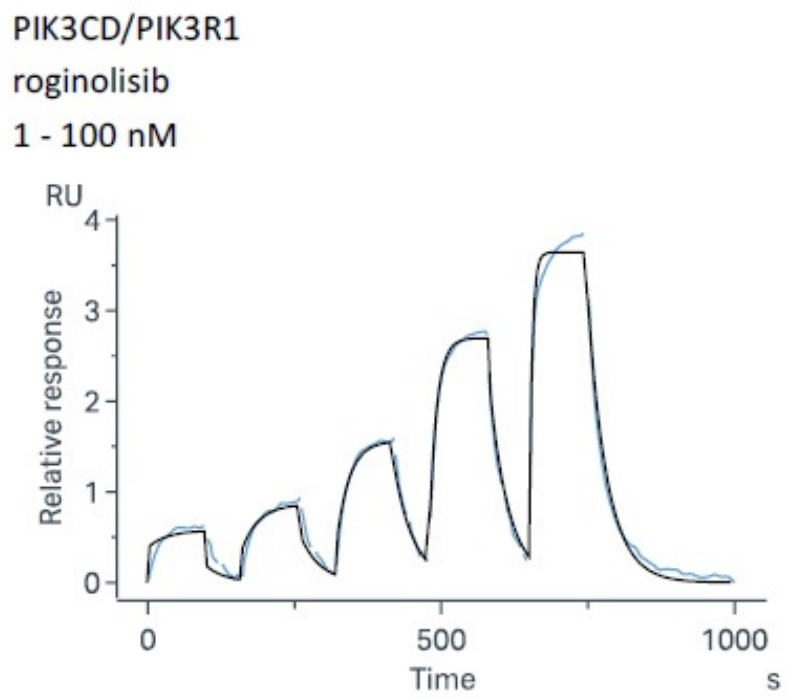

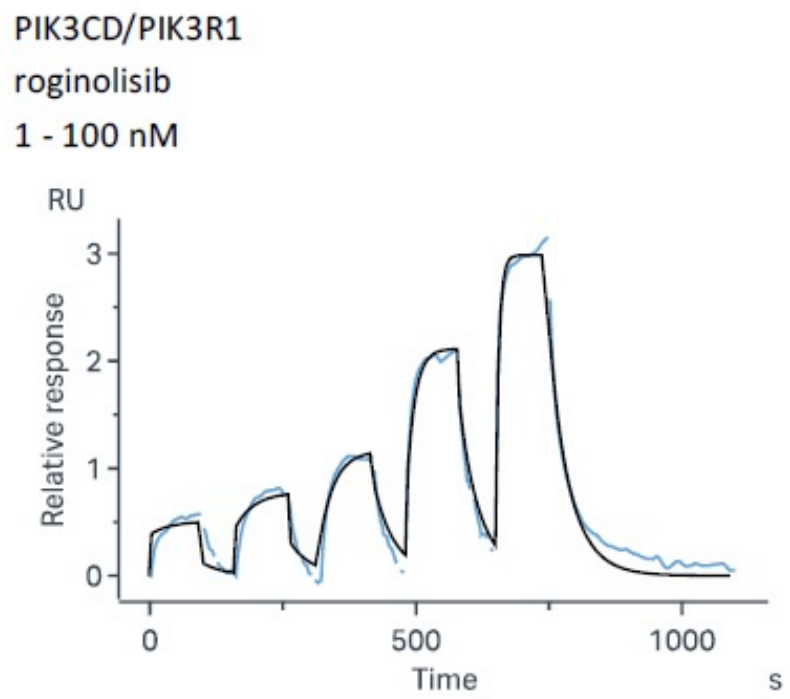

ka (1/Ms)	kd (1/s)	KD (M)	Rmax	SE(Rmax) (RU)	Chi^2 (RU^2)	SE(ka) (1/Ms)	SE(kd) (1/s)
1.63E+06	3.04E-02	1.87E-08	3.7	1.51E-02	9.23E-03	2.26E+04	4.58E-04

ka (1/Ms)	kd (1/s)	KD (M)	Rmax	SE(Rmax) (RU)	Chi^2 (RU^2)	SE(ka) (1/Ms)	SE(kd) (1/s)
1.36E+06	2.93E-02	2.15E-08	2.8	1.66E-02	1.13E-02	3.14E+04	7.37E-04

b)

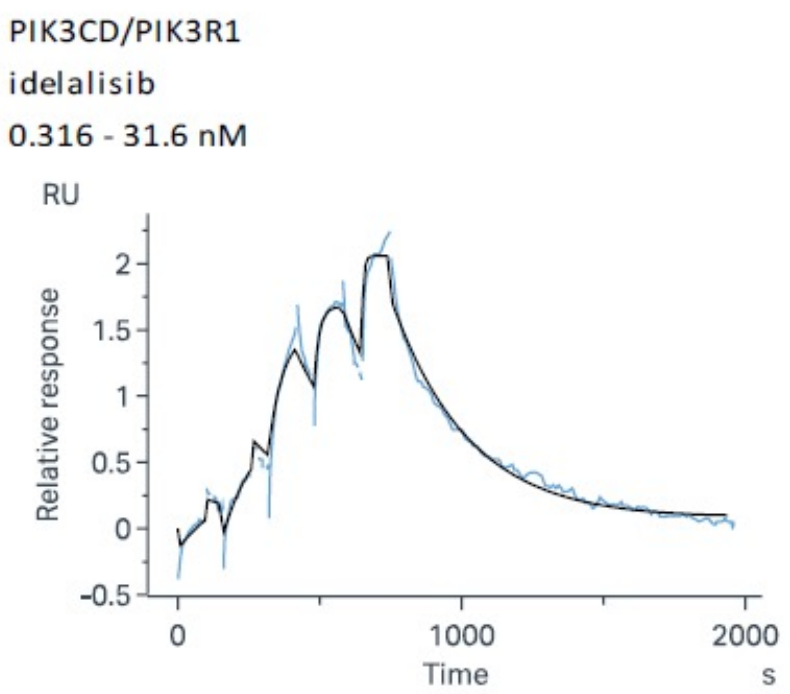

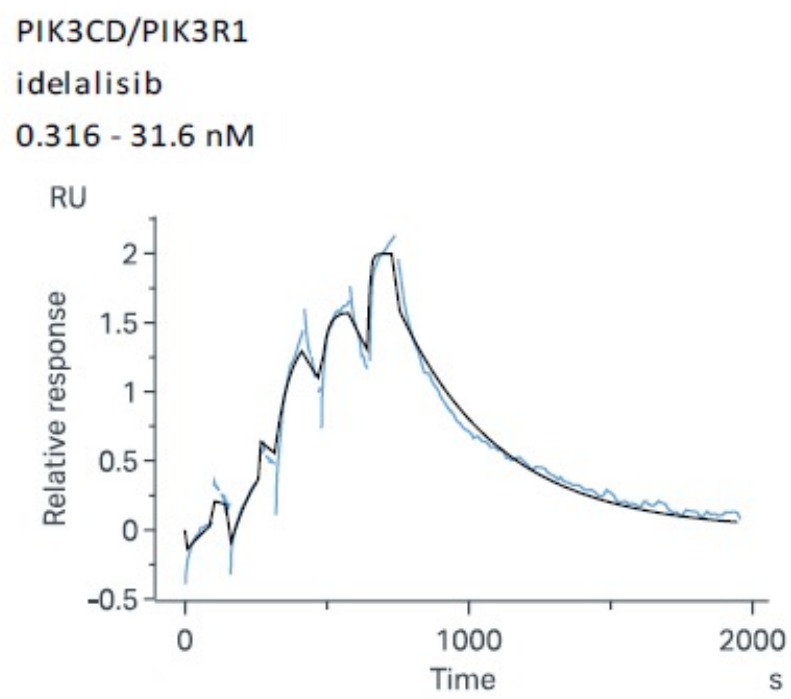

ka (1/Ms)	kd (1/s)	KD (M)	Rmax	SE(Rmax) (RU)	Chi^2 (RU^2)	SE(ka) (1/Ms)	SE(kd) (1/s)
5.09E+06	3.61E-03	7.08E-10	1.8	2.71E-03	4.62E-03	3.79E+04	2.04E-05

ka (1/Ms)	kd (1/s)	KD (M)	Rmax	SE(Rmax) (RU)	Chi^2 (RU^2)	SE(ka) (1/Ms)	SE(kd) (1/s)
5.01E+06	2.79E-03	5.58E-10	1.6	2.25E-03	5.46E-03	3.02E+04	7.50E-06

c)

Compound	k_a (1/Ms)	k_d (1/s)	K_D (M)	$t_{1/2}$ (s)	τ (s)
roginolisib	1.49E+06	2.98E-02	2.01E-08	23	34
idelalisib	5.05E+06	3.17E-03	6.28E-10	218	315

ka, association rate constant; kd, dissociation rate constant; KD, equilibrium dissociation constant; Rmax, maximum response; RU, response units; SE, standard error; Chi^2, goodness-of-fit statistic.

Supplementary Figure 3

a,**b** Binding kinetics of roginolisib (**a**) and idelalisib (**b**) against PIK3CD/PIK3R1, as measured by surface plasmon resonance and performed in duplicate. For every compound the curves were independently analysed and the resulting association constants (ka), dissociation constants (kd), binding constants (KD), maximum response (Rmax); Chi², goodness-of-fit statistic (Chi2), with respective standard error (SE) are reported in a-b. **c** Average values of the indicated parameters.

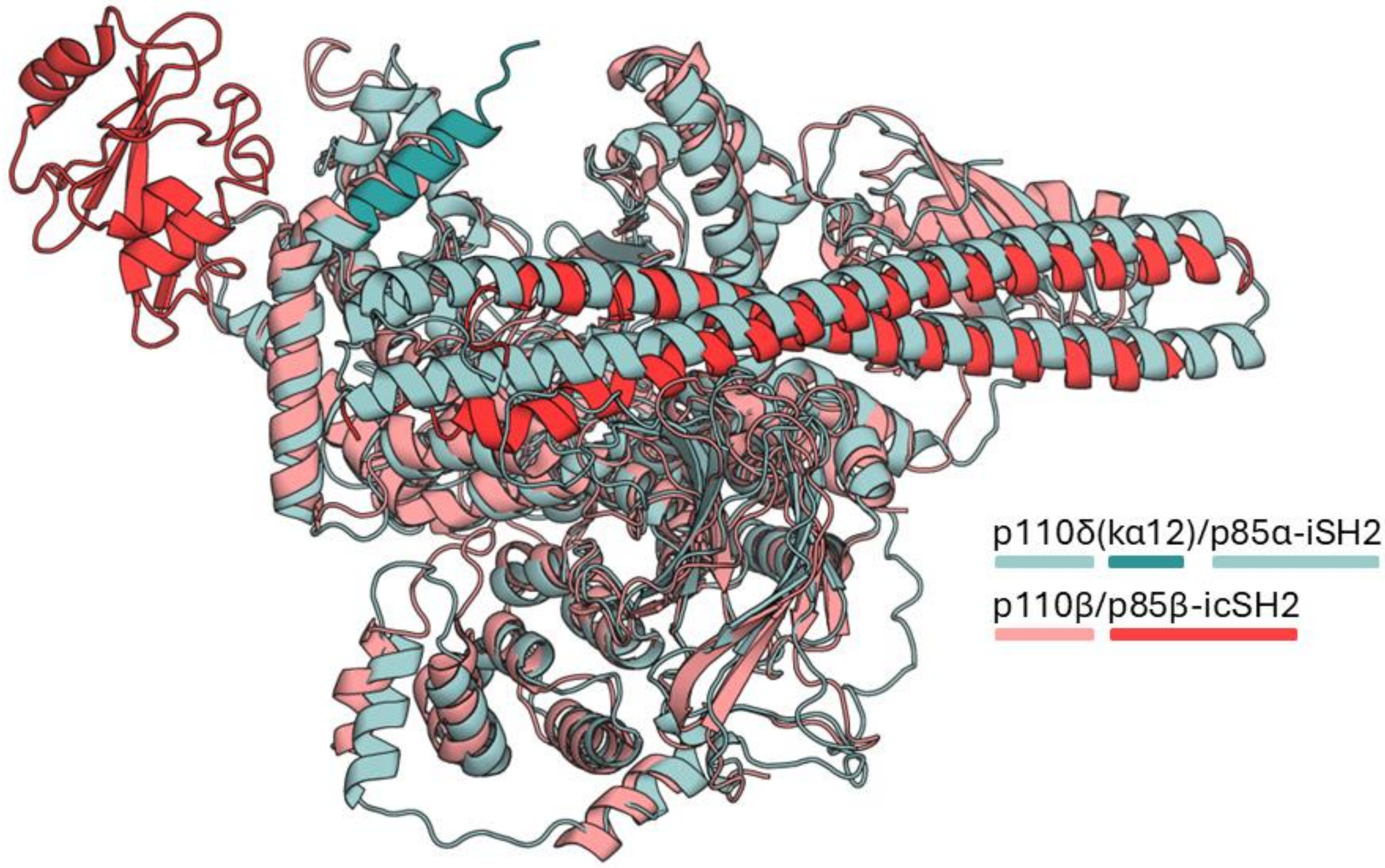

Supplementary Figure 4

Alignment of the modelled full-length p110δ structure (teal) and the p110β/p85β-icSH2 crystal structure (red). The p110δ C-terminal region unresolved in the crystal structure but modelled with Alphafold predictions and the p85β-icSH2 domains are highlighted in darker colours.

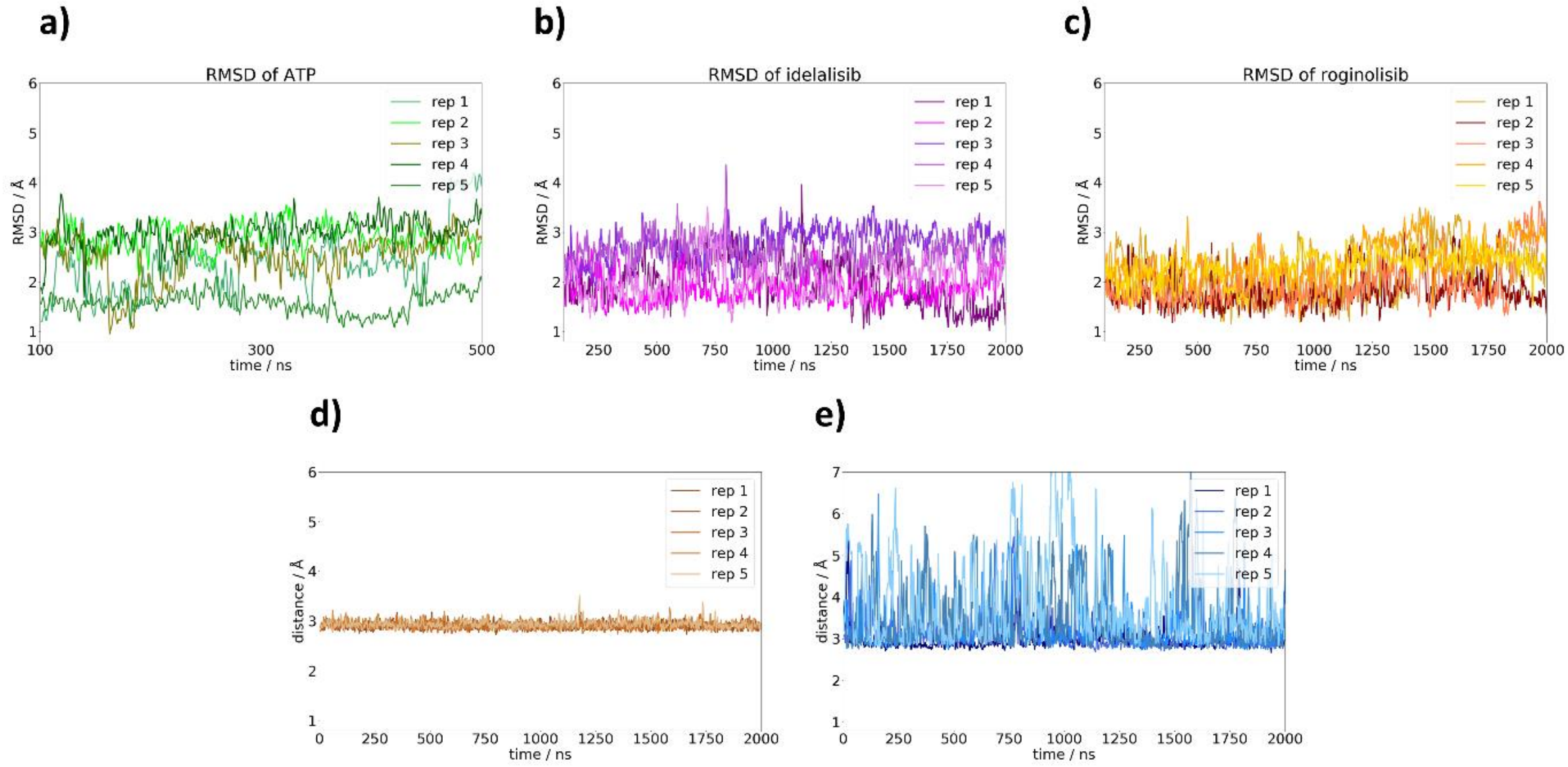

Supplementary Figure 5

Heavy-atom root mean square deviation (RMSD) and hydrogen bonding assessment of bound ligands as a function of simulation time. The raw data were processed using moving average smoothing with a window size of five frames (equivalent to 5 ns), except for the ATP RMSD plot, for which a window size of two frames (2 ns) was applied due to its shorter timescale. The RMSD was calculated for (a) ATP, (b) roginolisib, and (c) idelalisib upon superimposition of p110δ α-helix residues 866–889, 784–804, and 676–704 to their initial coordinates in each equilibrated simulation system. Distance measurements were taken between (d) the morpholine oxygen atom of roginolisib and the backbone nitrogen of Val828, and between (e) the sulphone oxygen of roginolisib interacting with the terminal nitrogen of Lys779.

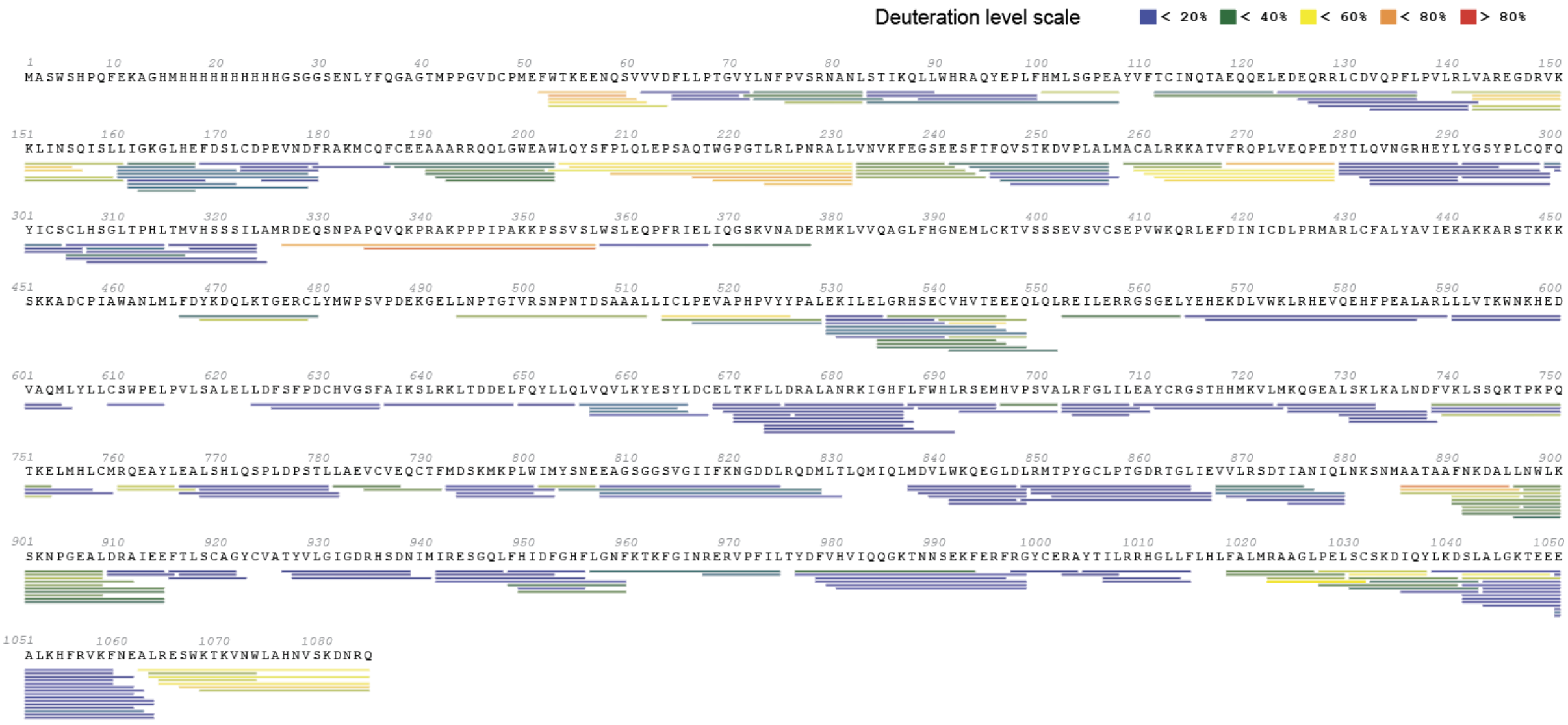

Supplementary Figure 6

Recombinant p110δ peptide map. Each line represents a high-quality peptide that was analysed to measure its H/D exchange rate. Peptides are colored according to their dynamics based on average deuteration level following the legend, with highly dynamic peptides shown in red and protected peptides colored in blue.

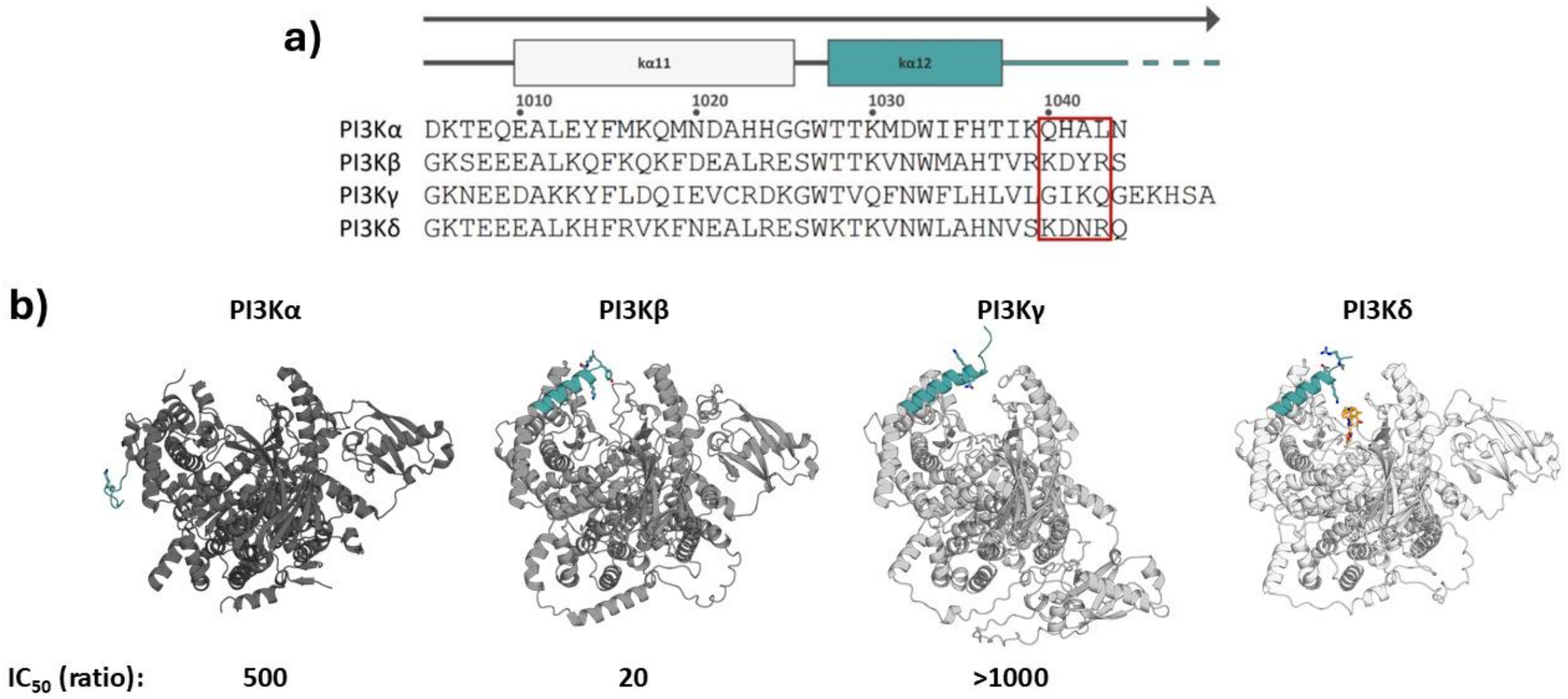

Supplementary Figure 7

C-terminal sequence variation among class I PI3K isoforms. **a** Sequence alignment towards the C-terminus of PI3K isoforms α–δ. Key residues of C-terminus (teal) are framed in red. **b** Structures of PI3K isoforms α–δ and their corresponding IC_{50} ratio relative to PI3Kδ. A crystal structure of PI3Kα (PDB ID: 7PG5[ref: PubMed 37225977]) is shown alongside AlphaFold models [27] of isoforms β, γ, and δ. Residues highlighted in **a** are shown as sticks.

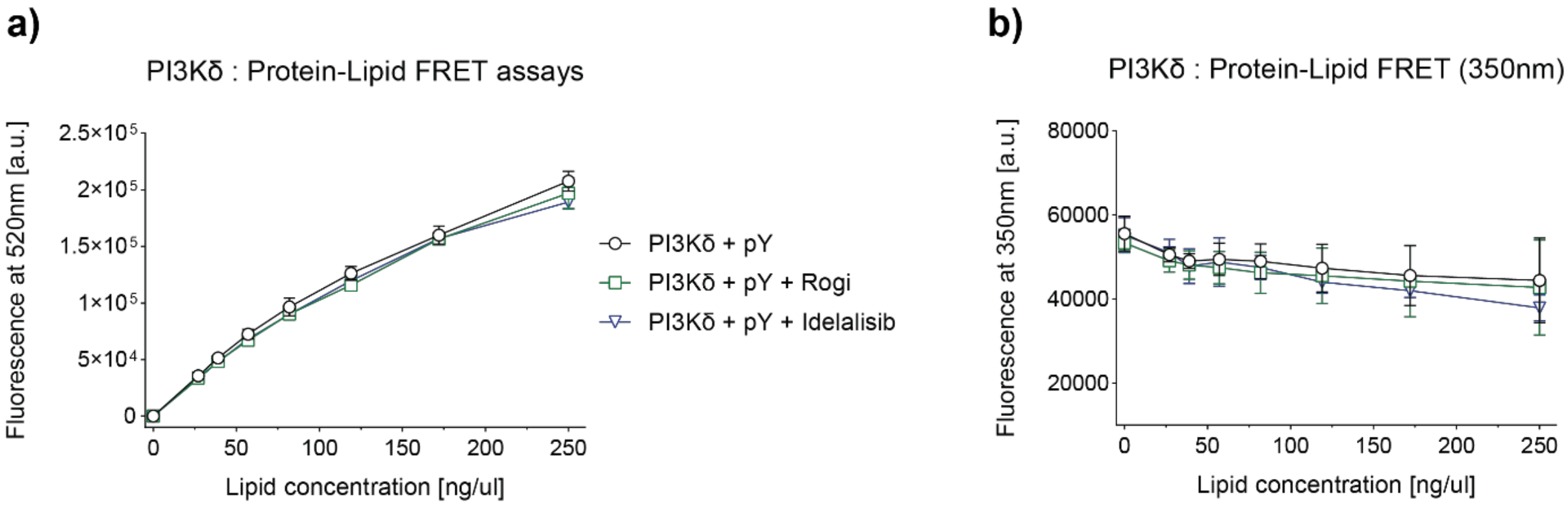

Supplementary Figure 8

Inhibitors do not influence interaction of phosphopeptide-activated PI3Kδ with membrane liposomes. Interaction was measured by protein-lipid FRET signal from intrinsic PI3Kδ tryptophan residues to DANSYL-containing liposomes. (a) Protein-lipid FRET signal measured with increasing lipid concentrations. All experiments done in presence of PDGFR phosphopeptide (pY) for the apo protein, in presence of roginolisib (Rogi) or in presence if idelalisib. (b) Fluorescence signal of Tryptophan to measure total PI3Kδ concentrations. Three independent experiments, results shown as mean +/- SD.

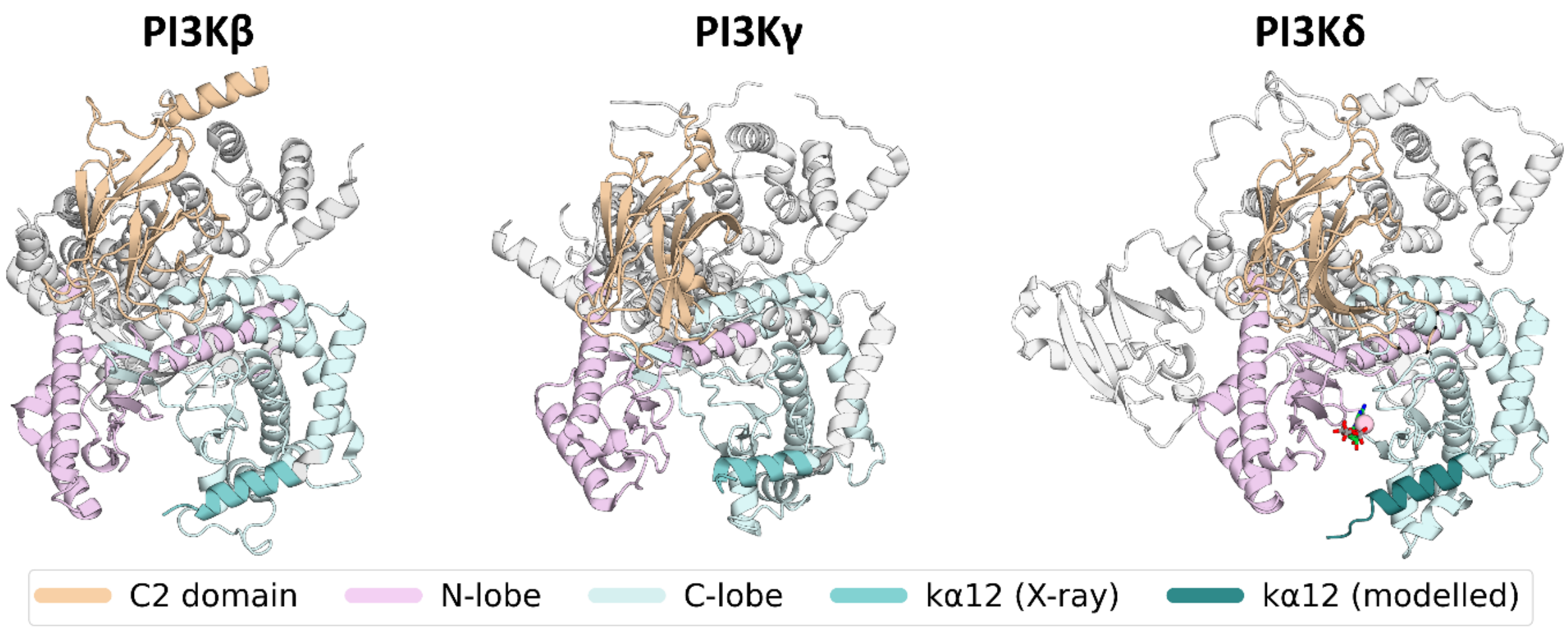

Supplementary Figure 9

PI3K C-terminus adopts slightly bent conformation in available structures of β and γ isoforms, with hinge between helix kα11 and kα12, and is predicted to be in similar conformation for δ isoform. Domains involved in membrane binding highlighted: C2 domain (light orange), kinase domain N-terminal lobe (light purple) and kinase domain C-terminal lobe (light blue). Crystal structures of β (left) and γ (middle) isoform with respective PDB ID 4BFR [46] and 1E7U [34] have resolved C-terminus (light teal). Crystal structure of ATP·Mg^{2+} (pink) bound δ isoform (right) with Alphafold2 [27] predicted and modelled kα12 helix (dark teal) shows C-terminus in similar orientation to β and γ isoform.

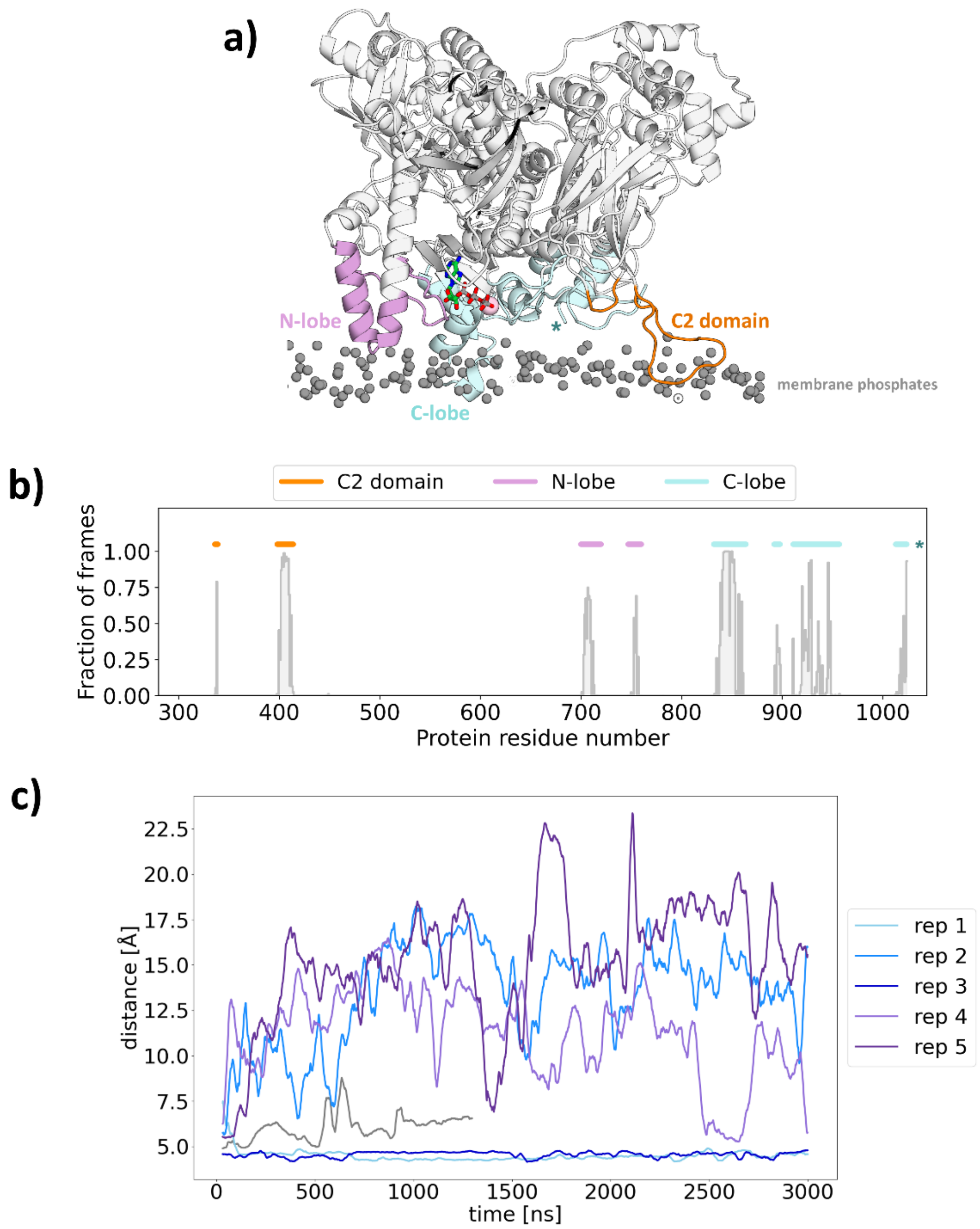

Supplementary Figure 10

Molecular dynamics simulations of membrane-bound p110δ upon deletion of C-terminal kα12 helix (residues 1025–1044). a Molecular graphic of a final simulation frame at 3 μs, showing ATP·Mg^{2+}-bound p110δ with regions of membrane contact highlighted. The position of the kα12 truncation is indicated by an asterisk. b Membrane contact map of protein residues across five simulation replicates each with a duration of 3 μs. All three major regions of membrane contact were maintained throughout simulations. c Distance between ATP γP and P5 of PIP2 molecule that entered the p110δ active site. Data are shown for five kα12-deleted simulation replicates (blue) following independent equilibration. A plot of the ATP–PIP2 distance in the original simulation of full-length p110δ after PIP2 entry into the active site is shown in grey for comparison.

Summary of crystallographic information for PI3Kδ · roginolisib.

PDB-ID	**9T07**
Data collection and processing	
X-ray source	PXI/X06SA (SLS[1])
Wavelength (Å)	0.9999
Detector	PILATUS 6M
Temperature [K]	100
Space group	$P2_12_12_1$
Unit cell parameters	
a, b, c (Å)	93.03, 113.39, 144.51
α, β, γ (°)	90.0, 90.0, 90.0
Resolution (Å)	2.75 (3.00-2.75)[2]
Unique reflections	39,134 (8,981)[2]
R_{sym} (%)[3]	3.5 (44.4)[2]
R_{meas} (%)[4]	4.0 (49.3)[2]
Completeness (%)	96.7 (97.9)[2]
Redundancy	4.8 (4.9)[2]
Mean I/σ(I)[5]	24.06 (3.23)[2]
Refinement	
Resolution range (Å)	46.52 – 2.75
Reflections used in refinement (work / free)	39,134 / 746
Final R values for all reflections (work[6] / free[7]) (%)	23.2 / 27.2
No. Atoms	
Proteins	8,749
Ligand	37
Water	28
RMSZ	
Bonds (Å)	0.66
Angles (°)	1.06
Ramachandran parameters (%)[8]	
Preferred regions	94.4
Allowed regions	4.9
Outliers	0.7

[1] SWISS LIGHT SOURCE (SLS, Villigen, Switzerland)

[2] values in parenthesis refer to the highest resolution bin.

[3] $$R_{sym} = \frac{\sum_h \sum_i^{n_h} |\widehat{I}_h - I_{h,i}|}{\sum_h \sum_i^{n_h} I_{h,i}} \text{ with } \widehat{I}_h = \frac{1}{n_h}\sum_i^{n_h} I_{h,i}$$

where $I_{h,i}$ is the intensity value of the ith measurement of h

[4] $$R_{meas} = \frac{\sum_h \sqrt{\frac{n_h}{n_h - 1}} \sum_i^{n_h} |\widehat{I}_h - I_{h,i}|}{\sum_h \sum_i^{n_h} I_{h,i}} \text{ with } \widehat{I}_h = \frac{1}{n_h}\sum_i^{n_h} I_{h,i}$$

where $I_{h,i}$ is the intensity value of the ith measurement of h

[5] calculated from independent reflections

[6] $R_{work} = (\sum |(F_{obs} - F_{calc})| / \sum |F_{obs}|)$

[7] $R_{free} = R_{work}$ based on ~1000 (at least 5%) of reflections excluded from refinement

[8] Calculated using COOT

Supplementary Table 1 : Summary of crystallographic information for PI3Kδ · roginolisib.

HDX experimental details

Dataset :	State 1	State 2	State 3	State 4	State 5	State 6	State 7	State 8	State 9
Description :	PI3Kd_WT_apo	PI3Kd_K1040A apo	PI3Kd_Y685A apo	PI3Kd_WT rogi	PI3Kd_K1040A rogi	PI3Kd_Y685A rogi	PI3Kd_WT idelalisib	PI3Kd_K1040A idelalisib	PI3Kd_Y685A idelalisib
**Control sample :**	Non-deuterated (ND) PI3Kd_WT_apo	Non-deuterated (ND) PI3Kd_WT_apo	Non-deuterated (ND) PI3Kd_WT_apo	Non-deuterated (ND) PI3Kd_WT_apo	Non-deuterated (ND) PI3Kd_WT_apo	Non-deuterated (ND) PI3Kd_WT_apo	Non-deuterated (ND) PI3Kd_WT_apo	Non-deuterated (ND) PI3Kd_WT_apo	Non-deuterated (ND) PI3Kd_WT_apo
**D2O incubation times (sec) :**	3s 30s 300s	3s 30s 300s	3s 30s 300s	3s 30s 300s	3s 30s 300s	3s 30s 300s	3s 30s 300s	3s 30s 300s	3s 30s 300s
**Replicates :**	2 2 3	3 2 3	3 3 3	2 3 3	3 3 3	3 3 3	3 2 3	3 3 3	3 3 3
**Sequence coverage :**	86.2%	86.2%	86.2%	86.2%	86.2%	86.2%	86.2%	86.2%	86.2%
**Number of peptides anayzed :**	283	283	283	283	283	283	283	283	283
**Reaction volume :**	50 ul	50 ul	50 ul	50 ul	50 ul	50 ul	50 ul	50 ul	50 ul
**% D2O in the reaction :**	86.3%	86.3%	86.3%	86.3%	86.3%	86.3%	86.3%	86.3%	86.3%
**Temperature :**	22 °C	22 °C	22 °C	22 °C	22 °C	22 °C	22 °C	22 °C	22 °C
**Protein amount (pmol) :**	**109**	**109**	**109**	**109**	**109**	**109**	**109**	**109**	**109**
**Protein concentration in reaction (µM) :**	**3.0**	**3.0**	**3.0**	**3.0**	**3.0**	**3.0**	**3.0**	**3.0**	**3.0**
**Protein concentrated mix volume (µl) :**	5.0	5.0	5.0	5.0	5.0	5.0	5.0	5.0	5.0
**Quench volume (µl) :**	20	20	20	20	20	20	20	20	20
**Deuterated buffer :**	10 mM Tris pH 7.5, 100 mM NaCl 100 uM Inhibitor (or DMSO)								
**Quench buffer :**	7 M UREA, 1 M NaCl, 100 mM NaH2PO4 (pH 2.4), 1 % Formic Acid (FA)								
**Criteria for HDX rate difference :**	Sum of difference for all timepoints between two states (sum-%D * sum-#D) is >5								

Supplementary Table 2 : HDX summary table describing experimental conditions

Figure Panel	Comparison	p-value
9ab	idela 2h vs rogi 2h	0.4706
9ab	idela 2h vs idela 24h	0.4474
9ab	idela 2h vs rogi 24h	0.2978
9ab	idela 24h vs rogi 2h	0.8235
9ab	idela 24h vs rogi 24h	0.6465
9ab	rogi 2h vs rogi 24h	0.4036
9cd	idela 2h vs idela 24h (2.5uM)	0.8662
9cd	idela 2h vs rogi 2h (2.5uM)	0.0397
9cd	idela 2h vs rogi 24h	0.4299
9cd	idela 24h vs rogi 2h	0.2892
9cd	idela 24h vs rogi 24h	0.1184
9cd	rogi 2h vs rogi 24h	0.3137
9ef	idela 2h vs idela 24h	0.6494
9ef	idela 2h vs rogi 2h	0.307
9ef	idela 2h vs rogi 24h	0.674
9ef	idela 24h vs rogi 2h	0.1941
9ef	idela 24h vs rogi 24h	0.851
9ef	rogi 2h vs rogi 24h	0.0014

Supplementary Table 3 : p values for phosphoinositide lipid profiling comparisons
