# Supplementary material for: PI3Kδ is selectively inhibited by roginolisib by stabilizing the C-terminal helix kα12
Source: Nat Commun. 2026 Oct 9;17:10220. doi: 10.1038/s41467-026-77424-0 (PMC13652804; doi:10.1038/s41467-026-77424-0)
Supplement: Supplementary file 2 [file 41467_2026_77424_MOESM2_ESM.pdf]

Description of Additional supplementary files

Supplementary Data 1. Raw values for all the HDX-MS experiments

Supplementary Data 2. Average deuteration values for all HDX-MS conditions

Supplementary Movie 1. Molecular dynamics simulation of ATP-bound PI3Kδ.
All-atom molecular dynamics simulation of PI3Kδ with ATP bound in the active site over 500 ns, visualized in VMD. ATP is shown in licorice representation and colored green, and the associated Mg^{2+} ion is depicted as a pink sphere. The protein residues Trp760, Lys779, and Val828 are highlighted in licorice representation. The trajectory was smoothed using a VMD trajectory smoothing window size of 3 and rendered at 30 frames per second. The movie illustrates the stable binding mode of ATP within the catalytic pocket throughout the simulation.

Supplementary Movie 2. Molecular dynamics simulation of Idelalisib-bound PI3Kδ.
All-atom molecular dynamics simulation of PI3Kδ with Idelalisib bound in the active site over 2 μs, visualized in VMD. Idelalisib is shown in licorice representation and colored purple. The protein residues Trp760, Lys779, and Val828 are highlighted in licorice representation. The trajectory was smoothed using a VMD trajectory smoothing window size of 3 and rendered at 60 frames per second. The movie demonstrates the stable binding of Idelalisib within the PI3Kδ binding site over the course of the simulation.

Supplementary Movie 3. Molecular dynamics simulation of Roginolisib-bound PI3Kδ.
All-atom molecular dynamics simulation of PI3Kδ with Roginolisib bound in the active site over 2 μs, visualized in VMD. Roginolisib is shown in licorice representation and colored orange. The protein residues Trp760, Lys779, and Val828 are highlighted in licorice representation. The trajectory was smoothed using a VMD trajectory smoothing window size of 3 and rendered at 60 frames per second. The movie shows the stable binding of Roginolisib within the PI3Kδ binding site throughout the simulation.
