# Supplementary material for: PI3Kδ is selectively inhibited by roginolisib by stabilizing the C-terminal helix kα12
Source: Nat Commun. 2026 Oct 9;17:10220. doi: 10.1038/s41467-026-77424-0 (PMC13652804; doi:10.1038/s41467-026-77424-0)
Supplement: Supplementary file 4 [file 41467_2026_77424_MOESM9_ESM.pdf]

nature portfolio

Peer Review File

PI3Kδ is selectively inhibited by roginolisib through stabilizing of the C-terminal helix kα12

Corresponding Author: Professor Gerhard Hummer

This file contains all reviewer reports in order by version, followed by all author rebuttals in order by version.

Attachments originally included by the reviewers as part of their assessment can be found at the end of this file.

Version 0:

Reviewer comments:

Reviewer #1

(Remarks to the Author)
Vadas et al. present a detailed biophysical investigation of PI3Kδ inhibition by roginolisib, and compare it to the first generation inhibitor, idelalisib. Their research highlights some important differences between the two inhibitors, and yields new insight into PI3 kinase mechanisms. This is a really interesting study, with the conclusions well supported by multiple experimental techniques. I only have some minor recommendations.

Regarding the presentation of the data in Figure 9:
• I would prefer to have the “100” marked on the y-axis in the naïve CLL plot to make it a bit easier to see where there has been an increase rather than a decrease of PIP3 levels compared to vehicle.
• I think that maybe rather than the column graph format, tracing the lines between individual samples at the 2 and 24 hr time points would make it easier to see the changes in the individual patient samples, which is perhaps more relevant than whether the overall average goes up or down.

Reviewer #2

(Remarks to the Author)
In this manuscript, the authors convincingly carried out an in depth and thorough analysis of the mode of binding and inhibition of roginolisib against PI3Kdelta, uncovering the mechanism through which the inhibitor shows selectivity against that specific enzyme isoform. The study employs a well-structured and systematic methodology based on a wide range of complementary techniques. The overall findings appear empirically sound and are consistent with the final conclusions.

Regarding the FRET data, the assay strategy choosen is somewhat nonideal. By holding the acceptor-labeled membrane fixed and titrating the donor-labeled protein, a variable background is introduced that complicates the isolation of the sensitized emission signal from donor bleedthrough. Ideally, a donor-labeled protein concentration should be kept fixed while the acceptor labeled membrane concentration is modified. The result donor quenching generates a signal that is directly proportional to interaction. In the strategy followed by the authors, sensitized emission will increase simply due to the presence of added acceptor protein. Results can only then be analyzed in a relative basis, through comparison with control experiments. Nevertheless, while the strategy choosen for the assay is non ideal, the conclusions presented by the authors seem adequate. It is not clear to me how the parameter (I - I0) represented in Fig S8B is calculated. The difference in intensities I- I0 are expected to be much larger than the values presented, as "I" at 500nM of protein is shown in Fig S8A to be around 2x10^4, while I0 values seem to be close to 0. The exact nature of the parameter shown in Fig S8 should be stated, clarifying the observed discrepancy.

Minor comments:
(page 21): This sentence should be revised for accuracy:
"The FRET signal shown in Fig S8 has I-Io along the Y axis where I is the intensity of 520 with protein and Io is the intensity of lipid alone."
should be changed to:

"The FRET signal at 487 nm shown in Fig S8 has I - I0 along the Y axis where I is the intensity at 520 nm in the presence of protein and I0 is the intensity of the sample in the absence of protein."

Reviewer #3

(Remarks to the Author)
I have been asked to specifically review the mass spectrometry–based approach to quantify PIP3 in cell lysates from CLL patient samples. I have the following queries:
1. For analysis of PIP3 the authors have not stated the PIP3 species they detected and what fragments they have used. What is the internal standard?
2. The data analysis and presentation as percentage of vehicle is unclear. The authors state they use internal standards so why are they not comparing total PIP3 in the vehicle to other conditions/timepoints? The main observation in the results text is that ROGI depletes PIP3 at 2h in patient samples, but this is not captured in the statistical analysis.
3. The data is very variable, as expected for PIP3 measurements. Why have the authors only included 3 biological samples per group? If patient samples are the limitation here could a cell line be used to support these observations?
4. PIP3 turnover is very fast (minutes instead of hours). Would it make more sense to analyse PIP3 after 15 minutes ROGI treatment?

Reviewer #4

(Remarks to the Author)
The manuscript describes how roginolisib, a selective PI3Kδ inhibitor currently in clinical evaluation, achieves both potency and isoform selectivity, and how its mechanism differs from the first-generation PI3Kδ inhibitor idelalisib. Using an approach that combines X-ray crystallography, all-atom molecular dynamics (MD) simulations, and hydrogen–deuterium exchange mass spectrometry, the authors propose that roginolisib uniquely stabilizes the C-terminal kα12 helix in an inward, autoinhibitory conformation, thereby locking PI3Kδ in an inactive state. They argue that this conformation-selective inhibition provides a mechanistic basis for roginolisib's high PI3Kδ selectivity and may help explain its more sustained pathway suppression relative to ATP-competitive inhibition.

The review below is limited to the evaluation of the MD simulations presented in this manuscript.

From a methodological standpoint, the MD setup is sound and broadly consistent with current best practices in the field. The simulations employ state-of-the-art force fields, include realistic and complex membrane compositions, and use reasonable equilibration and burn-in procedures, with multiple replicates and simulations reaching the microsecond timescale.

That said, several aspects of the MD analysis and reporting would benefit from clarification or additional quantification in order to strengthen the conclusions drawn from the simulations:

1. Modeling of unresolved regions. The manuscript would benefit from a clearer and more explicit description of which amino acid sequences were unresolved crystallographically and subsequently added by modeling. These regions appear central to the mechanistic interpretation, particularly the C-terminal helices, and greater transparency is important for both reproducibility and interpretation.

2. Use of AlphaFold versus homology modeling. The authors state that AlphaFold was used to model unresolved regions, including the kα12 helix of p110δ. However, they subsequently compare this model to an available experimental template. The rationale for using AlphaFold rather than conventional homology modeling should therefore be clarified, especially given the availability of a structurally related template. A brief discussion of whether AlphaFold was expected to add value beyond template-based modeling in this specific case would strengthen the methodological justification.

3. Quantification of ligand pose stability. While the authors state that ligand binding is stable during the simulations, the reported ligand RMSDs appear quite high for small molecules. Although RMSD is a coarse descriptor, values of this magnitude raise questions about pose stability versus sampling of alternative binding modes. A more quantitative assessment would be useful. For example, comparing ligand–receptor interaction fingerprints (hydrogen bonds, hydrophobic contacts, π–π interactions) derived from the MD ensembles with those observed in the crystal structures would help establish how closely the simulations reproduce the experimentally observed binding modes and identify any meaningful deviations.

4. Ligand parameterization details. Unless I missed them, details regarding ligand parameterization are currently missing. Given the central role of ligand stability in the conclusions, this information is essential. The authors should specify how partial charges and bonded/non-bonded parameters were derived, and whether validation checks were performed.

5. Data availability and reproducibility. To enhance transparency and reproducibility, I strongly encourage the authors to deposit the ligand parameter files, along with the initial coordinates, simulation input files, and at least one representative final coordinate file, either as supplementary material or via a public repository. This would substantially increase the long-term value of the work to the community.

Minor comment:
In Figure 1c, the ligand poses would be easier to interpret if shown in the same orientation as Figure 1b, facilitating direct

visual comparison.

In summary, the MD simulations are competently executed using accepted methodologies and provide an interesting framework to interpret the experimental data. Addressing the points above, particularly regarding ligand-protein interaction fingerprints, ligand parameters, and modeling choices, would significantly strengthen the rigor, clarity, and reproducibility of the computational component of the study.

Reviewer #5

(Remarks to the Author)
The manuscript by Vadas and colleagues represents an outstanding contribution to understanding how selectivity can be achieved in class I PI3K inhibitors. This is especially important for PI3Kdelta where idealisib was exquisitely selective for the delta isotype, but led to adverse, toxic consequences in clinical trials. There are several important firsts in the present manuscript. First, they have characterized in detail how Roginolisib, which has had very promising clinical trials works, and they have suggested plausible mechanisms for PI3Kdelta selectivity. Second, they have suggested a credible importance of the C-terminal helix for the PI3Kbeta and PI3Kdelta isotypes. This arose from the insight gained by their MD simulations, starting with a model suggested by Alphafold. Third, they have shown a clear difference between how idealisib and roginolisib interact with the enzyme, including the unique property of Roginolisib to stabilize the inhibitory interaction of the C-terminal helix with the active site. This effect is clearly absent in idealisib. Fourth, they have measured a decrease in membrane association of the enzyme brought on by the inhibitors. This was measured in a FRET assay and was facilitated by the MD analysis. Fifth, the MD analysis also suggested a rational explanation for how kalpha 12 is important for catalysis since the same basic residues that bind to the catalytic site in the absence of PIP2 substrate are employed in binding with PIP2 after association with the membrane. A strength of the paper is the highly effective and informative combination of X-ray crystal structures, alphafold, and HDX-MS. The following minor points should be addressed by the authors.

1. Why are the authors calling Roginolisib a non-ATP competitive inhibitor, when it occupies the ATP-binding site? There is clearly no way that Roginolisib and ATP could be present at the same time, given the structures. This is ATP-competitive. ATP might be a very poor binder compared with Roginolisib, but that does not mean that they are not competitive.
2. The legend for Fig S2 is unclear. Apparently, the authors have taken the structures for each step in the MD, aligned the protein atoms to some reference, then calculated the RMSD for the ligand with respect to a reference structure. What are the PDB ids for each of the reference structures? They are presumably the crystal structures for the two inhibitor complexes. What is the reference for the ATP structure? As far as I know, there is no experimental structure. Is this the alphafold model of PI3Kdelta with ATP? What were the protein atoms that were aligned to the reference? It is not clear that all atoms of the model should be aligned since the focus is the ligand atoms, presumably, an alignment of atoms near the ligand would be most appropriate. The authors should state which atoms were used. One of the replicas of the idealisib seems to have lost ligand binding. The text says there was no change throughout the MD.
3. Figure 2 is a compelling illustration of the dynamics of the C-terminal helix.
4. Lines 192-193. The text refers to four regions. From the figure, it is not clear what these regions are. It looks like three regions. It might be simplest to list them in the text.
5. Fig 4a. It looks like the differences portrayed are Roginolisib - apo and Idelalisib - apo. Why does the legend above each plot say WT +/- Roginolisib and WT +/- Idealisib. Everything here is WT. It is important to be unambiguous what is being subtracted (the subtrahend).
6. Fig 5a. The text says that HDX is greater for the Y685A mutant than the WT, and this is consistent with previous results. However, above the first plot in 5a, the legend says “Apo – Y685A vs WT”. Given that the numbers on the Y-access are positive for the ka12 region, I am guessing that what is being displayed is “Y685A – WT, for the Apo state”. This might be a clearer legend for the plot.
7. Line 217, Y685 should be Y685A.
8. As in #6 above, the legends for 5b should be “Roginolisib – Apo for the Y685A” and “Idealisib – Apo for the Y685A”
9. Lines 301-302. Should be “more robust suppression of the kinase activity compared to simple ATP competitive inhibition of the active kinase” See #1 above.
10. Supplemental figure S1 describes the map as 2Fo-Fc. I am guessing this is actually 2mFo-Dfc, the sigma-weighted map. It should be stated in the legend as such.
11. Fig S2 residues should be labeled. It is not clear what it means to say that the structure with idealisib is closer to DFG out, since according to the text, the phe is in the same conformation in both structures. If the authors really want to compare, they should state what the PI3K structure it is that they are using as DFG out.
12. Lines 358-360 are an outstanding summary of the inability of even high concentrations of ATP to compete with Roginolisib. However, it is completely misleading to call it a non-ATP competitive inhibitor.
13. Lines 470-472, please att supplier and catalogue numbers for the lipids used.

Reviewer #6

(Remarks to the Author)
Please see attached review

Version 1:

Reviewer comments:

Reviewer #1

(Remarks to the Author)
All my concerns have been addressed.

Reviewer #2

(Remarks to the Author)
The authors addressed my comments is a satisfactory manner, having modified the manuscript accordingly.

Reviewer #3

(Remarks to the Author)
Thank you for the clarification on your LC-MS/MS methodology. An important correction would be to make clear whether you are using 'internal standards', which are heavy-isotope labelled standards added at a known concentration at the start of your lipid extraction, or running 'standards' to confirm the m/z and retention time of your PIP3 species.

I am also unsure of how the data is presented with 'PIP3 ISTD/vehicle' on the y-axis- is this clarified in the figure legend which I cannot find in the 'Merged File containing manuscript text and 9 Figure files'? It is not in the methods.

Reviewer #4

(Remarks to the Author)
The authors have appropriately addressed all of my comments.

Reviewer #5

(Remarks to the Author)
The authors have revised the manuscript, and the revision addresses all of my concerns.

Reviewer #6

(Remarks to the Author)
The authors have addressed all concerns.

Version 2:

Reviewer comments:

Reviewer #3

(Remarks to the Author)
Thank you for the clarification of your methodology which is now much clearer. While I agree that odd acyl chains (17:0) are normally present at lower abundances than even chains, I am concerned about the abundance of naturally occurring 17:0 acyl chains in your samples. Can you confirm that phosphoinositides containing 17:0 acyl chains are absent in your biological samples, facilitating the use of 17:0 as an internal standard?

Open Access This Peer Review File is licensed under a Creative Commons Attribution 4.0 International License, which permits use, sharing, adaptation, distribution and reproduction in any medium or format, as long as you give appropriate credit to the original author(s) and the source, provide a link to the Creative Commons license, and indicate if changes were made.
In cases where reviewers are anonymous, credit should be given to 'Anonymous Referee' and the source.
The images or other third party material in this Peer Review File are included in the article's Creative Commons license, unless indicated otherwise in a credit line to the material. If material is not included in the article's Creative Commons license and your intended use is not permitted by statutory regulation or exceeds the permitted use, you will need to obtain permission directly from the copyright holder.
To view a copy of this license, visit https://creativecommons.org/licenses/by/4.0/

Reviewer's Comments:

Reviewer #1 (Remarks to the Author)

Vadas et al. present a detailed biophysical investigation of PI3Kδ inhibition by roginolisib, and compare it to the first generation inhibitor, idelalisib. Their research highlights some important differences between the two inhibitors, and yields new insight into PI3 kinase mechanisms. This is a really interesting study, with the conclusions well supported by multiple experimental techniques. I only have some minor recommendations.

We thank the reviewer for the positive and constructive comments and we hope that our answers address the concerns raised.

Regarding the presentation of the data in Figure 9:

• I would prefer to have the "100" marked on the y-axis in the naïve CLL plot to make it a bit easier to see where there has been an increase rather than a decrease of PIP3 levels compared to vehicle.

• I think that maybe rather than the column graph format, tracing the lines between individual samples at the 2 and 24 hr time points would make it easier to see the changes in the individual patient samples, which is perhaps more relevant than whether the overall average goes up or down.

RESPONSE: The y-axis of the naïve CLL plot has been revised to clearly indicate the 100-reference line and facilitate comparison with vehicle-treated samples in the following figure (Ex-figure 9) and in the **new figure 9B,D,F**, complemented with additional data. Moreover, the changes in the individual patient samples are now presented to improve clarity.

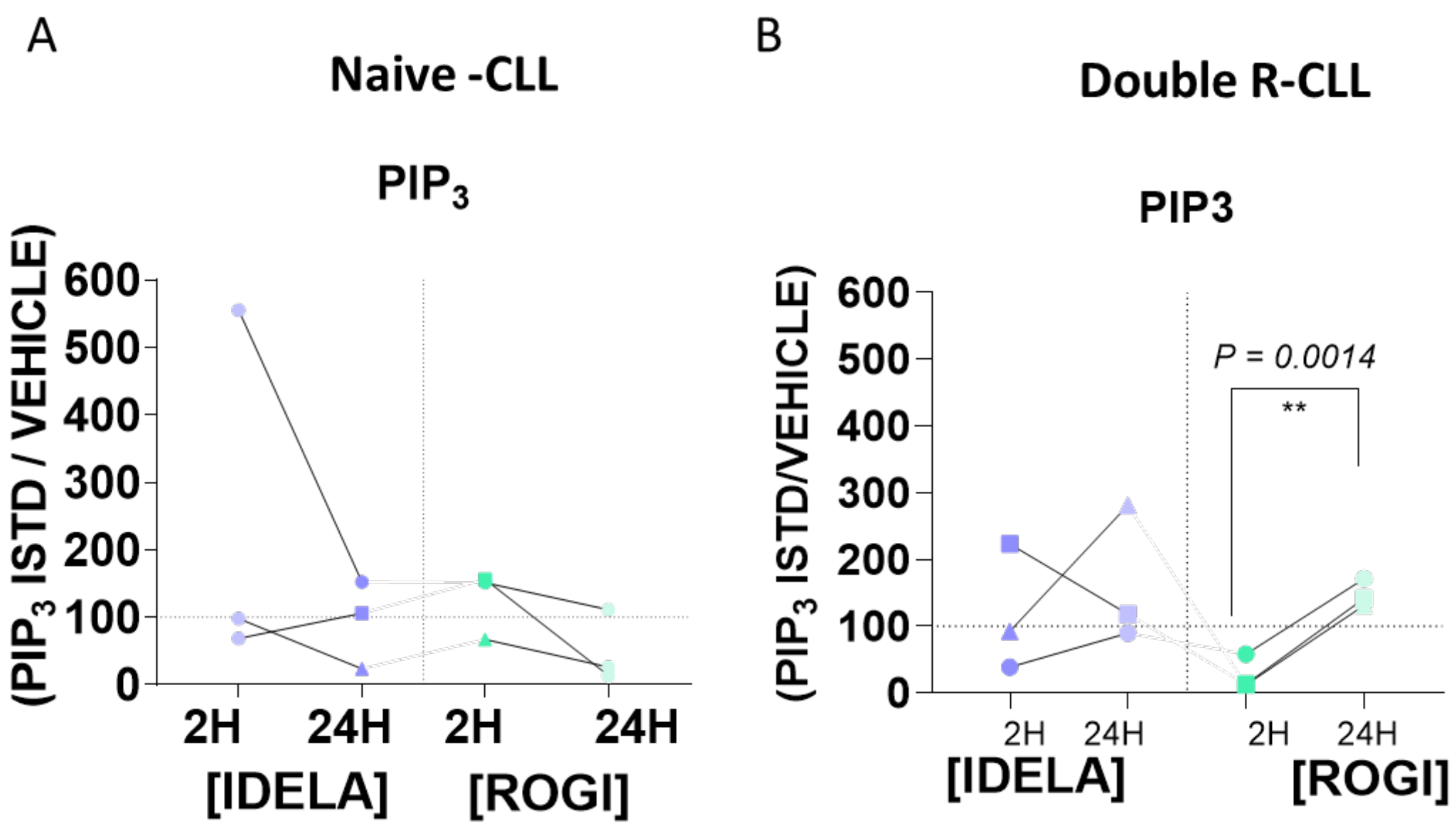

Ex - Figure 9 **Lipidomic profiling of phosphoinositide molecular species in circulating tumor cells (CTCs) from naïve CLL patients and resistant CLL patients (Double R-CLL) using LC-MS and TMS-Derivatization for enhanced detection of PIP_3 (n=3).**

Reviewer #2 (Remarks to the Author)

In this manuscript, the authors convincingly carried out an in depth and thorough analysis of the mode of binding and inhibition of roginolisib against PI3Kdelta, uncovering the mechanism through which the inhibitor shows selectivity against that specific enzyme isoform. The study employs a well-structured and systematic methodology based on a wide range of complementary techniques. The overall findings appear empirically sound and are consistent with the final conclusions.

We thank the reviewer for the positive and constructive comments and we hope that our answers address the concerns raised.

Regarding the FRET data, the assay strategy choosen is somewhat nonideal. By holding the acceptor-labeled membrane fixed and titrating the donor-labeled protein, a variable background is introduced that complicates the isolation of the sensitized emission signal from donor bleedthrough. Ideally, a donor-labeled protein concentration should be kept fixed while the acceptor labeled membrane concentration is modified. The result donor quenching generates a signal that is directly proportional to interaction. In the strategy followed by the authors, sensitized emission will increase simply due to the presence of added acceptor protein. Results can only then be analyzed in a relative basis, through comparison with control experiments. Nevertheless, while the strategy choosen for the assay is non ideal, the conclusions presented by the authors seem adequate. It is not clear to me how the parameter (I - I0) represented in Fig S8B is calculated. The difference in intensities I- I0 are expected to be much larger than the values presented, as "I" at 500nM of protein is shown in Fig S8A to be around 2x10^4, while I0 values seem to be close to 0. The exact nature of the parameter shown in Fig S8 should be stated, clarifying the observed discrepancy.

RESPONSE: The comment on experimental approach is absolutely valid. To address the reviewer's comment and validate the initial observation that inhibitors had no effect on PI3Kδ-membrane interaction, we performed the suggested experiment where enzyme concentration was kept constant and dansyl-containing liposomes were added in increasing amounts. The new data replace the previous data and are shown in the Figure S8. The conclusion is identical, thus the manuscript was minimally modified to describe the updated results:

"When testing on vesicles mimicking plasma membrane composition, neither roginolisib nor idelalisib impacted membrane interaction (Supplementary Fig. S8). stimulation with pY showed a 4-fold increase in membrane affinity, with an approximately 20% decrease upon inhibitor binding (NEW supplementary Fig. S88). This shows that the change in conformations of membrane-interacting kα1-kα2 and kα12 helices have only a limited effect on lipid interactions, at least for the pY-stimulated PI3Kδ heterodimer."

The methodology section was modified to describe the novel approach adopted.

Minor comments:
(page 21): This sentence should be revised for accuracy:
"The FRET signal shown in Fig S8 has I-Io along the Y axis where I is the intensity of 520 with protein and Io is the intensity of lipid alone."
should be changed to:
"The FRET signal at 487 nm shown in Fig S8 has I - I0 along the Y axis where I is the intensity at 520 nm in the presence of protein and I0 is the intensity of the sample in the absence of protein."

RESPONSE: We thank the reviewer for this suggestion and have modified according to the suggestion.

Reviewer #3 (Remarks to the Author)

I have been asked to specifically review the mass spectrometry–based approach to quantify PIP3 in cell lysates from CLL patient samples.

I have the following queries:
1. For analysis of PIP3 the authors have not stated the PIP3 species they detected and what fragments they have used. What is the internal standard?

RESPONSE: The detected PIP3 species were C32, C34, C36, C38; C38 were the most abundant in patient samples. The monitored fragments were the following:

**Name**	**Transition**
L_PIP_17.20_4	1012.5 -> 613.5
L_PIP_32_0	950.5 -> 551.5
L_PIP_32_1	948.5 -> 549.5
L_PIP_32_2	946.5 -> 547.5
L_PIP_32_3	836.5 -> 545.5
L_PIP_34_0	978.6 -> 579.5
L_PIP_34_1	976.5 -> 577.5
L_PIP_34_2	974.5 -> 575.5
L_PIP_36_0	1009.5 -> 607.5
L_PIP_36_1	1004.6 -> 605.5
L_PIP_36_2	1002.5 -> 603.5
L_PIP_36_3	1000.5 -> 601.5
L_PIP_36_4	998.6 -> 599.5
L_PIP_38_0	1034.5 -> 635.5
L_PIP_38_1	1032.5 -> 633.5
L_PIP_38_2	1030.5 -> 631.5
L_PIP_38_3	1028.5 -> 629.5
L_PIP_38_4	1026.5 -> 627.5
L_PIP_38_5	1024.5 -> 625.5

L_PIP2_17.20_4	1120.6 -> 613.4
L_PIP2_32_0	1058.6 -> 551.5
L_PIP2_32_1	1056.6 -> 549.5
L_PIP2_32_2	1054.5 -> 547.5
L_PIP2_32_3	1052.5 -> 545.5
L_PIP2_34_0	1086.6 -> 579.5
L_PIP2_34_1	1084.6 -> 577.5
L_PIP2_34_2	1082.6 -> 575.5
L_PIP2_36_0	1114.6 -> 607.6
L_PIP2_36_1	1112.6 -> 605.6
L_PIP2_36_2	1110.6 -> 603.6
L_PIP2_36_3	1108.6 -> 601.6
L_PIP2_36_4	1106.6 -> 599.6
L_PIP2_38_0	1142.7 -> 635.6
L_PIP2_38_1	1140.6 -> 633.6
L_PIP2_38_2	1138.6 -> 631.6
L_PIP2_38_3	1136.6 -> 629.6
L_PIP2_38_4	1134.6 -> 627.6
L_PIP2_38_5	1132.6 -> 625.6
L_PIP3_16_17	1163.0 -> 565.0
L_PIP3_17.20_4	1228.5 -> 613.5
L_PIP3_32_0	1166.5 -> 551.5
L_PIP3_32_1	1164.5 -> 549.5
L_PIP3_32_2	1162.5 -> 547.5
L_PIP3_34_0	1194.5 -> 579.5
L_PIP3_34_1	1192.5 -> 577.5
L_PIP3_34_2	1190.5 -> 575.5
L_PIP3_36_0	1222.5 -> 607.5
L_PIP3_36_1	1220.5 -> 605.5
L_PIP3_36_2	1218.5 -> 603.5
L_PIP3_36_3	1216.5 -> 601.5
L_PIP3_36_4	1214.5 -> 599.5
L_PIP3_38_0	1250.6 -> 635.6
L_PIP3_38_1	1248.6 -> 633.6
L_PIP3_38_2	1246.6 -> 631.6
L_PIP3_38_3	1244.6 -> 629.6
L_PIP3_38_4	1242.5 -> 627.5
L_PIP3_38_5	1240.5 -> 625.5
L_PIP3_40_0	1278.6 -> 663.6
L_PIP3_40_1	1276.6 -> 661.6
L_PIP3_40_2	1274.6 -> 659.6
L_PIP3_40_3	1272.6 -> 657.6
L_PIP3_40_4	1270.6 -> 655.6
L_PIP3_40_5	1268.6 -> 653.6

L_PIP3_40_6	1266.6 -> 651.6

The internal standards were the following:

Lipid Standard	Species / Source	Reference Number	Supplier
PI4P Brain	—	840045	Avanti Research
PI(4,5)P2 Brain	—	840046	Avanti Research
PI(3,4,5)P3	18:0-20:4	850166	Avanti Research
PIP	17:0 20:4	—	Avanti Research
PI(5)P	17:0-20:4	LM1902-1EA	Avanti Research
PI(4,5)P_2	17:0-20:4	LM1904-1EA	Avanti Research
PI(3,4,5)P_3	17:0-20:4	LM1906-1EA	Avanti Research

The manuscript has been revised to include details on the PIP3 species detected and the internal standard used for the analysis.

"Detected PIP3 species were C32, C34, C36, C38; C38 were the most abundant in patient samples. Internal standards were obtained from Avanti Research: : PI4P Brain; #840045; PI(4,5)P2 Brain, #840046; PI(3,4,5)P3[18:0-20:4], #850166; PIP[17:0 20:4]; PI(5)P[17:0-20:4], #LM1902-1EA; PI(4,5)P_2[17:0-20:4], LM1904-1EA; PI(3,4,5)P_3 [17:0-20:4], LM1906-1EA".

2. The data analysis and presentation as percentage of vehicle is unclear. The authors state they use internal standards so why are they not comparing total PIP3 in the vehicle to other conditions/timepoints? The main observation in the results text is that ROGI depletes PIP3 at 2h in patient samples, but this is not captured in the statistical analysis.

RESPONSE: We have used the presentation as percentage to allow comparison of PIP3 evolution upon PI3Kδ inhibitor treatment, as the data in vehicle-treated cells are variable in each patient (see Figure 1 for Reviewer 3). We have now added more samples, which allow us to conclude more firmly on the observed effect with roginolisib. See also below response to question 3.

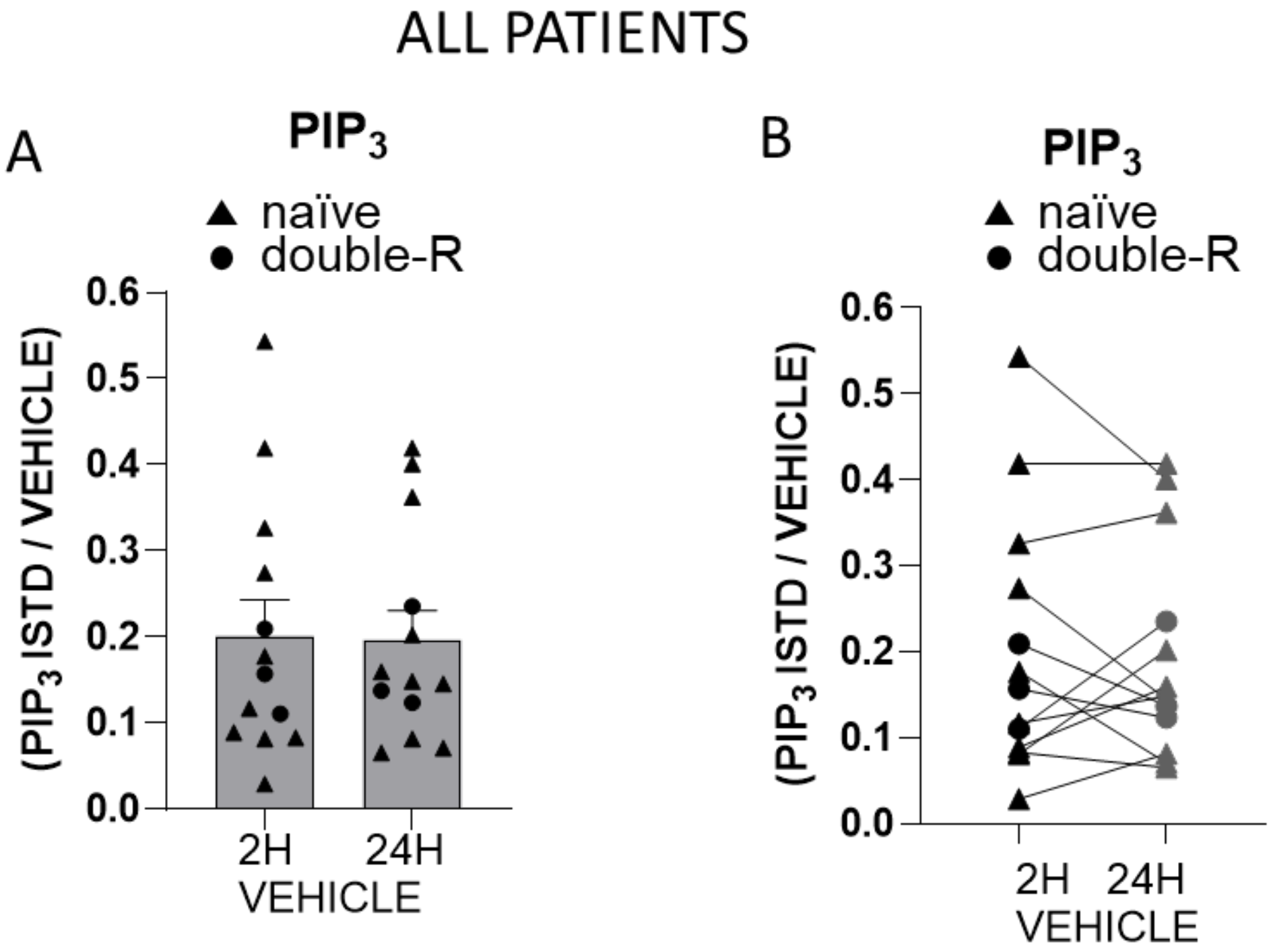

Figure 1 to reviewer 3 Basal PIP3 level in circulating tumor cells (CTCs) from naïve-of-treatment CLL patients (naïve, triangle) and resistant to treatment CLL patients (Double R, Circle) using LC-MS and TMS-Derivatization for enhanced detection of PIP3 (n=13). A, Mean ± SEM. B, Same results shown as evolution of PIP3 level in each patient.

3. The data is very variable, as expected for PIP3 measurements. Why have the authors only included 3 biological samples per group? If patient samples are the limitation here could a cell line be used to support these observations?

RESPONSE: We have added more samples in the low dose groups, which did not change our conclusion. At high dose, we now highlight a significant effect of roginolisib compared to idelalisib on PIP3 levels after 2 hours of treatment (New Figure 9). It was not possible for us to add more data on the double-refractory patient cells. The biobank only contains three double-refractory patient samples, which reflects the rarity and limited availability of this specific pathological condition, and there is no equivalent in cell lines for this pathological situation.

With those new results, the description has been revised as following: "The analysis revealed that roginolisib was more effective than idelalisib in decreasing PIP3 levels, at the 2-hour time point and at high dose (Fig. 9C,D). We observed heterogeneity of response in tested patients, in particular at low doses in idelalisib-treated circulating tumour cells. Idelalisib led to a heterogeneous response between patient cells, with one naïve and two double refractory patient cells presenting a more than 2-fold increase of PIP3 production compared to vehicle, possibly indicative of treatment resistance (Fig. 9A,E). Interestingly, all tested refractory patient cells had decreased PIP3 levels upon 2h treatment with roginolisib, which was significantly lower compared to 24h treatment (Fig. 9E), suggesting a differential feedback response."

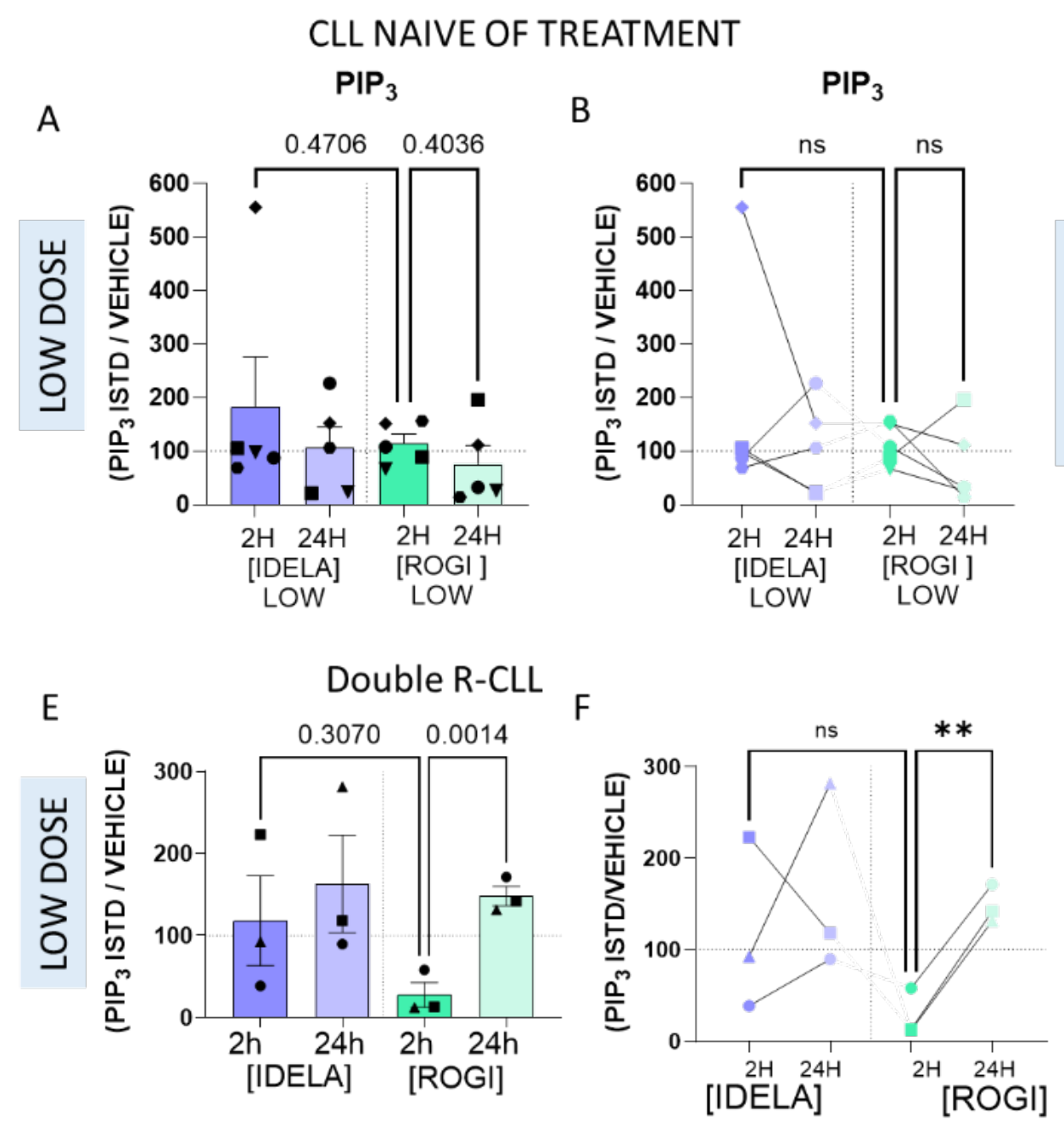

New Figure 9 **Basal PIP3 level in circulating tumor cells (CTCs) from treatment naïve and treatment resistant CLL patients treated with idelalisib or roginolosib for 2 or 24h hours, using LC-MS and TMS-Derivatization for enhanced detection of PIP3.**

4. PIP3 turnover is very fast (minutes instead of hours). Would it make more sense to analyse PIP3 after 15 minutes ROGI treatment?

RESPONSE: In CLL, tonic activation of the B-cell receptor (BCR) leads to constitutive, ligand-independent activation of PI3Kδ. Therefore, we were interested in evaluating the effect of the inhibitor using both short-term (2 h) and long-term (24 h) treatment conditions rather than a short time point (e.g. 15 minutes), which would capture immediate signaling fluctuations but not sustained pathway modulation.

Reviewer #4 (Remarks to the Author)

The manuscript describes how roginolisib, a selective PI3Kδ inhibitor currently in clinical evaluation, achieves both potency and isoform selectivity, and how its mechanism differs from the first-generation PI3Kδ inhibitor idelalisib. Using an approach that combines X-ray crystallography, all-atom molecular dynamics (MD) simulations, and hydrogen–deuterium exchange mass spectrometry, the authors propose that roginolisib uniquely stabilizes the C-terminal kα12 helix in an inward, autoinhibitory conformation, thereby locking PI3Kδ in an inactive state. They argue that this conformation-selective inhibition provides a mechanistic basis for roginolisib's high PI3Kδ selectivity and may help explain its more sustained pathway suppression relative to ATP-competitive inhibition.

We thank the reviewer for the positive and constructive comments, and we hope that our answers address the concerns raised.

The review below is limited to the evaluation of the MD simulations presented in this manuscript.

From a methodological standpoint, the MD setup is sound and broadly consistent with current best practices in the field. The simulations employ state-of-the-art force fields, include realistic and complex membrane compositions, and use reasonable equilibration and burn-in procedures, with multiple replicates and simulations reaching the microsecond timescale.

That said, several aspects of the MD analysis and reporting would benefit from clarification or additional quantification in order to strengthen the conclusions drawn from the simulations:

1. Modeling of unresolved regions. The manuscript would benefit from a clearer and more explicit description of which amino acid sequences were unresolved crystallographically and subsequently added by modeling. These regions appear central to the mechanistic interpretation, particularly the C-terminal helices, and greater transparency is important for both reproducibility and interpretation.

RESPONSE: We thank the reviewer for this comment. In response, we now clarify these important aspects of the modelling method in the revised manuscript (Methods, lines 458–468). In particular, we now list the amino acid sequences that were not resolved in the crystal structure and how they were modelled.

"Initial structures of full-length p110δ in complex with bound roginolisib, idelalisib (PDB ID: 4XE0 [50]), or ATP were modelled using MODELLER [51] based on the best-ranked p110δ Alphafold model and structures of the respective protein-ligand X-ray co-crystals. The final structural model for the roginolisib-bound protein consisted of the crystal structure with unresolved termini, loops, and their flanking regions taken from the Alphafold prediction (namely residues 1–22, 174–193, 221–243, 277–319, 396–418, 494–508, 839–857, 915–935, and 1007–1044). The same structural model was used for the ATP-bound protein, with the ATP binding pose taken from a crystal structure of ATP-bound porcine p110γ (PDB ID: 1E8X [34]) and the relative position of the coordinated magnesium ion taken from PDB ID: 1CSN [52] to generate the full complex. In the case of the idelalisib-bound complex, the crystallographically resolved murine structure was replaced by the full Alphafold model of the human protein upon structural alignment".

2. Use of AlphaFold versus homology modeling. The authors state that AlphaFold was used to model unresolved regions, including the kα12 helix of p110δ. However, they subsequently compare this model to an available experimental template. The rationale for using AlphaFold rather than conventional homology modeling should therefore be clarified, especially given the availability of a structurally related template. A brief discussion of whether AlphaFold was expected to add value beyond template-based modeling in this specific case would strengthen the methodological justification.

RESPONSE: We thank the reviewer for raising this point. In regions lacking experimental density, indeed both conventional homology modelling and deep learning-based prediction approaches such as AlphaFold (which integrates evolutionary information from multiple sequence alignments) provide established methods for generating plausible conformations. However, the suitability of homology modelling is directly

determined by the availability and quality of an appropriate structural template. In the case of p110δ, the most closely related protein with a structurally resolved kα12 helix is a murine orthologue of p110β, differing both in the kinase isoform and source species, with a sequence identity of 57.60% relative to human p110δ. Given this moderate template similarity, we based our structural models instead on the AlphaFold prediction to obtain a sequence-specific conformation and minimise bias from relying on a single template. Subsequent comparison with the murine p110β structure confirmed that the modelled kα12 helix adopts a conformation in agreement with the experimentally resolved homologue.

We now clarify in the manuscript that the homologous structure used for comparison is the murine p110β isoform (lines 152–153):

"To validate the modelled full-length p110δ structure, we compared it to the murine p110β/p85β-icSH2 crystal structure..."

3. Quantification of ligand pose stability. While the authors state that ligand binding is stable during the simulations, the reported ligand RMSDs appear quite high for small molecules. Although RMSD is a coarse descriptor, values of this magnitude raise questions about pose stability versus sampling of alternative binding modes. A more quantitative assessment would be useful. For example, comparing ligand–receptor interaction fingerprints (hydrogen bonds, hydrophobic contacts, π–π interactions) derived from the MD ensembles with those observed in the crystal structures would help establish how closely the simulations reproduce the experimentally observed binding modes and identify any meaningful deviations.

RESPONSE: We thank the reviewer for the helpful observation and comment. Our ligand RMSD analysis in the original submission had been calculated upon structural alignment of the full p110δ backbone, which led to values artificially inflated by global protein motions. We have revised the RMSD analysis (Figure S5) to more accurately reflect ligand stability, based on alignment on structurally stable α-helices surrounding the binding site (residues 866–889, 784–804, and 676–704). This resolves the apparently elevated RMSD values observed for idelalisib in replicate 3, which originated from protein flexibility rather than from a drift or rearrangement of the ligand binding pose. We added the movies rendered from the simulations showing the stable binding poses of idelalisib and roginolisib in the binding site as supporting information (Movie S1, S2).

In line with the reviewer's suggestion, we further assessed key ligand–receptor interaction fingerprints, namely two hydrogen bonds formed by roginolisib in the crystal structure (Figure S5d, e). The hydrogen bond between the morpholine oxygen and Val828 remained highly stable throughout the simulations, supporting conservation of the crystallographic binding mode. Hydrogen bonding between a sulphone oxygen and Lys779, whilst weaker and more dynamic, was consistently observed in all replicates. Its fluctuations arose from rotations of the Lys779 side chain, which occasionally swung away before returning to reform the interaction. This behaviour reflects flexibility in the lysine side chain whilst the ligand binding pose remains stable.

4. Ligand parameterization details. Unless I missed them, details regarding ligand parameterization are currently missing. Given the central role of ligand stability in the conclusions, this information is essential. The authors should specify how partial charges and bonded/non-bonded parameters were derived, and whether validation checks were performed.

RESPONSE: We apologise for the inadvertent omission in the original submission. The missing ligand parameterisation details have now been added (Methods, lines 472–474):

"Roginolisib and idelalisib were parameterised using the general Amber Force Field (GAFF) [PMID: 15116359]. ATP in the membrane simulations was parameterised via CHARMM-GUI (https://charmm-gui.org/) [PMIDs: 18351591; 26631602; 28497616]."

5. Data availability and reproducibility. To enhance transparency and reproducibility, I strongly encourage the authors to deposit the ligand parameter files, along with the initial coordinates, simulation input files, and at least one representative final coordinate file, either as supplementary material or via a public repository. This would substantially increase the long-term value of the work to the community.

RESPONSE: These have now been deposited at Zenodo: 10.5281/zenodo.17911609, as stated under the updated Data Availability:

"Full MD simulation input and coordinate files have been deposited at Zenodo: 10.5281/zenodo.17911609."

Minor comment:
In Figure 1c, the ligand poses would be easier to interpret if shown in the same orientation as Figure 1b, facilitating direct visual comparison.

RESPONSE: We thank the reviewer for this suggestion. We now show ligand poses in the same orientation in Figure 1c to enable direct visual comparison.

In summary, the MD simulations are competently executed using accepted methodologies and provide an interesting framework to interpret the experimental data. Addressing the points above, particularly regarding ligand-protein interaction fingerprints, ligand parameters, and modeling choices, would significantly strengthen the rigor, clarity, and reproducibility of the computational component of the study.

We thank the reviewer for the positive and constructive comments.

Reviewer #5 (Remarks to the Author)

The manuscript by Vadas and colleagues represents an outstanding contribution to understanding how selectivity can be achieved in class I PI3K inhibitors. This is especially important for PI3Kdelta where idealisib was exquisitely selective for the delta isotype, but led to adverse, toxic consequences in clinical trials. There are several important firsts in the present manuscript. First, they have characterized in detail how Roginolisib, which has had very promising clinical trials works, and they have suggested

plausible mechanisms for PI3Kdelta selectivity. Second, they have suggested a credible importance of the C-terminal helix for the PI3Kbeta and PI3Kdelta isotypes. This arose from the insight gained by their MD simulations, starting with a model suggested by Alphafold. Third, they have shown a clear difference between how idealisib and roginolisib interact with the enzyme, including the unique property of Roginolisib to stabilize the inhibitory interaction of the C-terminal helix with the active site. This effect is clearly absent in idealisib. Fourth, they have measured a decrease in membrane association of the enzyme brought on by the inhibitors. This was measured in a FRET assay and was facilitated by the MD analysis. Fifth, the MD analysis also suggested a rational explanation for how kalpha 12 is important for catalysis since the same basic residues that bind to the catalytic site in the absence of PIP2 substrate are employed in binding with PIP2 after association with the membrane. A strength of the paper is the highly effective and informative combination of X-ray crystal structures, alphafold, and HDX-MS.

We thank the reviewer for the positive and constructive comments, and we hope that our answers address the concerns raised.

The following minor points should be addressed by the authors.

1. Why are the authors calling Roginolisib a non-ATP competitive inhibitor, when it occupies the ATP-binding site? There is clearly no way that Roginolisib and ATP could be present at the same time, given the structures. This is ATP-competitive. ATP might be a very poor binder compared with Roginolisib, but that does not mean that they are not competitive.

RESPONSE: The reviewer is absolutely correct that roginolisib and ATP cannot bind at the same time given the structures. Nevertheless, as reported earlier by Johnson et al 2023 competition studies had found that inhibition of PI3Kδ by roginolisib is not affected by increasing ATP concentration, indicating that ATP may not dislodge bound roginolisib. This unexpected result motivated us in part to perform the work presented in this manuscript. Our data suggest that the stabilisation of the inhibitory interaction of the C-terminal helix with the active site likely reduces the binding affinity of ATP to PI3Kδ making roginolisib binding *appear* as non-ATP competitive.

Meanwhile, we have also determined the kinetic constants of the binding of roginolisib and idelalisib to PI3Kδ by surface plasmon resonance measurement using surface plasmon resonance (SPR). These data confirm the binding affinity of roginolisib and show fast on/off kinetics demonstrating that roginolisib binding is not irreversible and that the ATP pocket remains accessible for ATP binding. The fact that ATP appears not to compete with roginolisib for binding to the inactive conformation is therefore likely the result of a lower affinity of ATP for this enzyme state (new figure S3). The SPR data has been added to the manuscript (Results: lines 140-144 ; Discussion : lines 375-376).

Binding kinetics of roginolisib and idelalisib against PIK3CD/PIK3R1

Compound	k_a (1/Ms)	k_d (1/s)	K_D (M)	$t_{1/2}$ (s)	τ (s)
roginolisib	1.49E+06	2.98E-02	2.01E-08	23	34
idelalisib	5.05E+06	3.17E-03	6.28E-10	218	315

k_a, association rate constant; k_d, dissociation rate constant; K_D, equilibrium dissociation constant; $t_{½}$, half-life; τ, residence time

2. The legend for Fig S2 is unclear. Apparently, the authors have taken the structures for each step in the MD, aligned the protein atoms to some reference, then calculated the RMSD for the ligand with respect to a reference structure. What are the PDB ids for each of the reference structures? They are presumably the crystal structures for the two inhibitor complexes. What is the reference for the ATP structure? As far as I know, there is no experimental structure. Is this the alphafold model of PI3Kdelta with ATP? What were the protein atoms that were aligned to the reference? It is not clear that all atoms of the model should be aligned since the focus is the ligand atoms, presumably, an alignment of atoms near the ligand would be most appropriate. The authors should state which atoms were used. One of the replicas of the idealisib seems to have lost ligand binding. The text says there was no change throughout the MD.

RESPONSE: We thank the reviewer for highlighting the need to clarify the RMSD reference structures. All ligand RMSD values were calculated relative to their initial coordinates in each equilibrated simulation system. This information has now been added to the figure legend.

In the original submission, ligand RMSD had been calculated upon structural alignment of the full protein backbone of p110δ, which led to values that were artificially inflated by global protein motions. We have revised the RMSD analysis (Figure S5) to more accurately reflect ligand stability, based on alignment on structurally stable α-helices surrounding the binding site (residues 866–889, 784–804, and 676–704). This resolves the apparently elevated RMSD values observed for idelalisib in replicate 3, which originated from protein flexibility rather than ligand dissociation. The ligand remained stably bound across all simulations, as confirmed by the updated RMSD profiles and persistent hydrogen bonding interactions.

We believe these clarifications and methodological refinements address the reviewer's concerns and enhance the robustness of the analysis.

3. Figure 2 is a compelling illustration of the dynamics of the C-terminal helix.

RESPONSE: We thank the reviewer for this comment.

4. Lines 192-193. The text refers to four regions. From the figure, it is not clear what these regions are. It looks like three regions. It might be simplest to list them in the text.

RESPONSE: We thank the reviewer for this comment. In response, we have listed the regions in the text.

5. Fig 4a. It looks like the differences portrayed are Roginolisib - apo and Idelalisib - apo. Why does the legend above each plot say WT +/- Roginolisib and WT +/- Idealisib. Everything here is WT. It is important to be unambiguous what is being subtracted (the subtrahend).

RESPONSE: The text above the graphs for figure 4a have been modified thanks to the reviewer's suggestion to accurately describe the state compared: "WT apo vs WT + roginolisib" and "WT apo vs WT + idelalisib"

6. Fig 5a. The text says that HDX is greater for the Y685A mutant than the WT, and this is consistent with previous results. However, above the first plot in 5a, the legend says "Apo – Y685A vs WT". Given that the numbers on the Y-access are positive for the ka12 region, I am guessing that what is being displayed is "Y685A – WT, for the Apo state". This might be a clearer legend for the plot.
7. Line 217, Y685 should be Y685A.
8. As in #6 above, the legends for 5b should be "Roginolisib – Apo for the Y685A" and "Idealisib – Apo for the Y685A"

RESPONSE: The text above the graphs of figure 5a and 5d have been modified following the suggestions to accurately describe the state compared: "WT apo vs Y685A apo" ; "Y685A apo vs Y685A + robinolisib" and "Y685A apo vs Y685A + idelalisib" for figures 5a and 5d respectively.

9. Lines 301-302. Should be "more robust suppression of the kinase activity compared to simple ATP competitive inhibition of the active kinase" See #1 above.

RESPONSE: See response to point 1- In addition, we also replaced this sentence with the following: 'more consistent suppression of the kinase activity compared to classic inhibition of the active kinase at the plasma membrane'.

10. Supplemental figure S1 describes the map as 2Fo-Fc. I am guessing this is actually 2mFo-Dfc, the sigma-weighted map. It should be stated in the legend as such.

RESPONSE: We have corrected this in the legend of figure S1.

11. Fig S2 residues should be labeled. It is not clear what it means to say that the structure with idealisib is closer to DFG out, since according to the text, the phe is in the same conformation in both structures. If the authors really want to compare, they should state what the PI3K structure it is that they are using as DFG out.

RESPONSE: Labels have now been added for the Asp911 residue forming the DFG motif in both structures, adopting a DFG-in-like configuration in the presence of roginolisib and resembling a DFG-out-like conformation in the idelalisib-bound structure. In the text it is clarified that the phenylalanine orientation is in the DFG-in conformation for both structures.

12. Lines 358-360 are an outstanding summary of the inability of even high concentrations of ATP to compete with Roginolisib. However, it is completely misleading to call it a non-ATP competitive inhibitor.

RESPONSE: We apologize for the confusion as we have tried to avoid describing roginolisib as a non-ATP competitive inhibitor. The data suggest that roginolisib stabilizes an enzyme conformation with reduced affinity for ATP. This is now further supported by the measured roginolisib's binding kinetics using SPR showing a relatively short residence time, suggesting that roginolisib binding is highly reversible, and thus would be amendable for competition, see the data below. The table below was added to the new Fig. S3 and the text was modified to comment on the new SPR results

Binding kinetics of roginolisib and idelalisib against PIK3CD/PIK3R1

Compound	k_a (1/Ms)	k_d (1/s)	K_D (M)	$t_{1/2}$ (s)	τ (s)
roginolisib	1.49E+06	2.98E-02	2.01E-08	23	34
idelalisib	5.05E+06	3.17E-03	6.28E-10	218	315

k_a, association rate constant; k_d, dissociation rate constant; K_D, equilibrium dissociation constant; $t_{½}$, half-life; τ, residence time

13. Lines 470-472, please att supplier and catalogue numbers for the lipids used.

RESPONSE: Reference numbers and supplier were added for all the lipids.

Reviewer #6 (Remarks to the Author)

The authors propose the structural characterization of the interaction between PI3Kδ (composed of p110δ and p85α) and Roginolisib, a novel small molecule inhibitor that targets the PI3Kδ isoform (currently under phase II trials). This inhibitor shows high target specificity and a safety profile compared to the first generation of PI3Kδ inhibitors such as Idelasilib, used for the treatment of patients with relapsed chronic lymphocytic leukaemia (CLL) and indolent non-Hodgkin lymphoma (NHL). These first-generation inhibitors showed variable toxicity, potentially explained by insufficient selectivity among PI3K isoforms. PI3Kδ undergoes several steps from its autoinhibited form to a fully activated state, including notably a membrane localization through the binding to Ras proteins (to p110δ) and the binding of the phosphorylated tyrosine motif of RTK to p85's SH2 domain. Here, the authors used a combination of orthogonal approaches including crystallography, Molecular Dynamics simulations, HDX-MS, protein-lipid FRET assays and MS-based quantification of PIP3 to decipher the inhibition mechanism of PI3Kδ by Roginolisib and explain the high selectivity for the p110δ isoform.

Overall, the paper meets the requirements for a publication in this journal after addressing the comments described below.

The first part of the paper shows the structure elucidation involving the p110δ subunit bound to Roginolisib and its comparison with the structure of p110δ bound to Idelasilib, highlighting two different binding modes. Unlike Idelasilib, Roginolisib does not occupy the specificity pocket but binds some residues of it and engages the Asp911 of the DFG motif (involved in binding selectivity) in a DFG in like conformation.

COMMENTS:

- The authors compared both structures of human PI3Kδ/Roginolisib and murine PI3Kδ/Idelalisib and highlighted some differences in the binding mode. Could these differences be due to PI3Kδ's nature difference? Can the authors comment on that?

RESPONSE: Mouse and human PI3Kδ share a very high sequence homology in the ATP pocket, and the structures overlay very well. The idelalisib binding mode that addresses the specificity pocket in murine PI3Kδ, has been confirmed in human PI3Kδ with idelalisib-like inhibitors as well as structurally unrelated inhibitors (DOI: 10.1021/acsmedchemlett.6b00438; DOI: 10.62347/jcgq1004).

We modelled the human PI3Kδ/idelalisib complex based on the ligand binding configuration in the murine structure and performed molecular dynamics simulations of both roginolisib- and idelalisib-bound complexes with the human protein. The stability of both ligands within the binding pocket during simulations supports conservation of the crystal structure-derived idelalisib binding mode, which remains distinct from that of roginolisib.

It is therefore highly unlikely that the differences observed between roginolisib and idelalisib binding can be driven by species differences. Furthermore, the whole blood potency of roginolisib on rodent and human B cells is highly similar when corrected for plasma protein binding differences suggesting comparable binding affinity across species. (van der Veen et al. 2023)

We have updated the main text (line 156) to clarify the nature of the simulated structures:

"All-atom MD simulations were conducted on full-length human p110δ bound to either roginolisib, idelalisib, or ATP."

- In Figures S1 and S2, labelling the specific residues mentioned in the text would allow a better appreciation of the described interaction network (in Fig S1: inhibitors' names and residues number could be added and in Fig S2: Asp911 could be labelled).

RESPONSE: We thank the reviewer for the comment. Labels have now been added in Figures 1 and S2.

To further investigate the inhibition mechanism on the conformation dynamics of the p110δ C-ter region (especially kα12 for which they have no density), the authors performed MD simulations using AlphaFold prediction models of the p110δ subunit bound to either ATP, Roginolisib or Idelasilib: 1) The results showed a difference of the kα11-kα12 angle between the three bound states. 2) kα12 is closer to the active site in Roginolisib bound state, *via* Asp911 forming an aspartate cluster with Asp897 and maintaining electrostatic interactions with K1040 and R1043 (consistent with the autoinhibitory role of PI3Kδ C-ter region).

COMMENTS:

- The authors only modelled p110δ subunit for most of the simulations. They did not use p110 and p85 (although, they used both subunits in the following HDX-MS experiments). Can the authors comment on that?

RESPONSE: We thank the reviewer for raising this point. With the autoinhibitory p85δ cSH2 domain crystallographically unresolved, we did not model its binding onto p110δ to avoid introducing speculative interactions. Our MD simulations thus focused on the direct effects of ligand binding within the p110δ catalytic subunit, which contains the binding pocket. To account for any regulatory effects mediated by p85δ, both p110δ and p85δ were included in our HDX-MS experiments.

- In Figure S4, it would be appreciated to label the specific regions mentioned in the text (especially kα12) and have a clear legend to differentiate between the AlphaFold model and 2Y3A structure.

RESPONSE: We thank the reviewer for the suggestion. Figure S4 has been revised to include a legend that clearly labels the specific regions described in the text and distinguishes between the AlphaFold model and the 2Y3A structure.

- In Figures 3a and b, it would be appreciated to precise the name of the molecules on top of the structures like the authors did in Figure 2a.

RESPONSE: The molecule names have now been labelled in Figure 3.

Then the authors performed HDX-MS experiments to characterize the conformational dynamics of p110δ/p85 heterodimer upon Roginolisib and Idelasilib binding. The authors show that kα12 is protected upon Roginolisib binding only, consistent with the folding of the helix over the active site.

COMMENTS:

- The description of the HDX-MS experiments is missing in the Methods section. Please provide it for all experiments.

RESPONSE: We apologize for not including those in the initial submission, this was a mistake. A full description of the HDX-MS methodology is now available in the Methods section.

- Do the authors plan to submit the HDX-MS data to the Proteomics Identifications Database?

RESPONSE: All data related to the HDX-MS analysis are available in the PRIDE database (Project PXD071872). This is mentioned in the material and method section.

- In Fig S6, the deuteration level is shown for all identified peptides of p110δ. However, it is not mentioned for which timepoint in the description.

RESPONSE: The colouring is done by the software (HDExaminer) and represents the average deuteration level. The legend of Fig S6 has been modified to include that colouring of peptides is based on average deuteration level.

- In Figure 4b: Have the HDX-MS results been exported on the AlphaFold model?

RESPONSE: HDX-MS results are analyzed in specialized software, comparing the mass of deuterated peptides across all labelling times and across different states (Mostly the software HDExaminer). The results are then visualized by mapping the identified regions on a 3D model. For this experiment, the optimized model initiated by AlphaFold was used for plotting, as it represents the highest quality structure.

Next, the authors hypothesized that Roginolisib may target the inactive state of p110δ. To test it, they generated a Y685A mutation in p85α, part of the regulatory subunit that mediates the inhibitory contact between p110δ-cSH2 and the region connecting kα11 and kα12 (being catalytically more active). Then, they compared H/D exchange between WT and Y685A – where kα12 is deprotected in the mutant - and then characterized the exchange upon the binding of inhibitors. No changes are observed upon Idelasilib binding between the WT and Y685A while upon Roginolisib binding, no protection is observed for Y685A (unlike the WT), suggesting that Roginolisib makes additional contacts with the C-ter region in the inactive state, limiting kα12 flexibility upon activation.

COMMENTS:

- Have the authors observed some changes in the region of p85α spanning Y685 in the previous HDX-MS experiment?

RESPONSE: The effect of p85α-Y685A mutation on p85α dynamics was previously investigated and thus was not explored further in the current project. A published study showed that [p85α-Y685A] retained capacity to bind to phosphopeptides mimicking PDGFR receptor "*The peptide 681–687 in p85α that showed an increase in exchange in the presence of PDGFR pY in wild-type p85α, showed a decrease in exchange for the Y685A construct due to rigidification of the entire cSH2 domain in the presence of PDGFR pY*". For more details, please see Burke JE et al (2011) Structure, DOI: 10.1016/j.str.2011.06.003 .

- Have the authors compared the binding between the inhibitors and WT/Y685A?

RESPONSE: Such experiments are included in the manuscript. The labelling of the graphs in Fig 4 and Fig 5 has been modified to more accurately describe the compared states. HDX-MS analysis comparing apo-p110δ/p85α versus inhibitor-bound p110δ/p85α (results shown in Fig 4a), as well as apo-p110α/p85α-Y685A versus inhibitor-bound p110δ/p85α-Y685A (Fig 5d) are presented.

Next, the authors compared the C-ter region between the different p110δ isoforms highlighting the presence of K1040 and R1043 only in p110δ and β isoforms - consistent with IC50 measurements - explaining the high selectivity of Roginolisib towards the δ isoform and in a lesser extent the β isoform.

COMMENT:

- Do the authors have an idea explaining the IC50 difference (20 fold) between the δ and β isoform given that both have K1040 and R1043 residues?

RESPONSE: Although roginolisib binding to the β isoform may also induce a similar conformational stabilization of Kα12 due to the presence of the K1040 and R1043 residues, there are amino acid differences between the δ and β isoform in the ATP pocket, especially in the hinge region binding area (828-832). These differences may explain the different selectivity. A similar explanation has been suggested for other PI3Kδ selective inhibitors that do not occupy the specificity pocket. (DOI: 10.18632/oncotarget.8702)

Then the authors investigated if the presence of inhibitors affects PI3Kδ membrane interaction. They performed protein-lipid FRET and observed a decrease of 20% of membrane affinity in presence of inhibitors, showing that conformational changes in kα1-kα2 and kα12 upon inhibitor binding have a limited impact on lipid interactions. They next performed MD simulations of p110δ + ATP binding PIP2 containing leukocytic plasma membrane, leading to the insertion of kα12 into the membrane (being more ordered). The simulations in presence of a Δkα12-p110δ construct showed that p110δ remained bound to membrane (*via* the C2 domain).

RESPONSE: The new protein-lipid FRET experimental design suggested by reviewer #2 confirmed that both roginolisib and idelalisib have identical impact on membrane affinity. However, the overall effect of inhibitors on activated PI3Kδ affinity with membranes is no longer significant. The text has been modified to reflect this observation, which does not modify the model of inhibition:

"When testing on vesicles mimicking plasma membrane composition, neither roginolisib nor idelalisib impacted membrane interaction (Supplementary Fig. S8). This shows that the change in conformations of membrane-interacting kα1-kα2 and kα12 helixes have only a limited effect on lipid interactions, at least for the pY-stimulated PI3Kδ heterodimer."

COMMENTS:

- In Figures 6a, 6b and 7a, it would be appreciated if the authors could label the mentioned regions and kα12.

RESPONSE: Labels of protein regions have now been added in Figures 6 and 7.

- It is not clear where the authors are showing the results obtained for the simulations made in presence of the Δkα12 p110δ isoform: is it Figure 8?

RESPONSE: In the original submission, results for the Δkα12 p110δ simulations were described only in the text. To clarify our observation, we have added the new supplementary Figure S10 and expanded the description of these simulations (lines 294-300 and Methods, lines 498–501):

"To further examine the role of kα12 in lipid binding, we initiated additional simulations from the membrane-bound, PIP2-engaged p110δ configuration with kα12 deleted (Fig. S10a). While p110δ remained bound to the membrane in all five replicates, likely mediated by interaction of the C2 domain (Fig. S10b), the substrate PIP2 readily dissociated from the active site in three replicates (Fig. S10c). In the two remaining replicates, the PIP2 molecule remained associated through coordination with the magnesium ion. No other membrane PIP2 lipids were observed to approach the p110δ active site during a total simulation time of 15 μs across the five replicates."

"Based on the membrane-bound configuration at 2.4 μs, a separate set of five replicate simulations were initiated upon deletion of kα12 (residues 1025–1044) to generate an electrically neutral C-terminus, followed by re-solvation and equilibration before production simulations."

Finally, the authors investigated how the binding modes' differences between the inhibitors affect the kinase activity. To do so, the authors quantified by MS the PIP3 generated in cell lysates from CLL patients treated with inhibitors and showed that Roginolisib was more effective in decreasing PIP3 levels, suggesting a more efficient inhibition of the kinase activity for Roginolisib.

COMMENTS:

- Could the authors give more information about the methods used for the quantification and the statistical validation regarding the SRM approach?

RESPONSE: This question is not clear to us. Does the reviewer refer to the MS method for measuring PIP3? More information is given in response to comments from Reviewer #3 on this. Once the question is clarified, we would be happy to give more information.

- Do the authors plan to submit the MS data to the Proteomics Identifications Database?

RESPONSE: All data related to the HDX-MS analysis are available in the PRIDE database (Project PXD071872).

Minor comments:

- Homogenization of the legend description (especially the sub-figures labelling like a and b in Figure S1).

- Homogenization of inhibitors names (with or without a first capital letter)

- Homogenization of the word naïve/naïve

- When necessary (described in the comments), labelling secondary structures elements and regions in the figures where there are structures would be helpful for the reader.

- A bracket is missing l.229 in the main text.

RESPONSE: We adjusted in the text all the above minor comments. We thank the reviewer for highlighting these mistakes/incongruences.

Reviewer #3

Thank you for the clarification on your LC-MS/MS methodology. An important correction would be to make clear whether you are using 'internal standards', which are heavy-isotope labelled standards added at a known concentration at the start of your lipid extraction, or running 'standards' to confirm the m/z and retention time of your PIP3 species.

We thank the reviewer for this important comment. We confirm that we are using synthetic internal standards in each sample. The standards used are not heavy-isotope labelled, but are non-natural occurring standard (e.g. C17:0). A mixture of PIP/PIP2/PIP3 standards was added during final chloroform extraction and prior derivatization. All lipid species were normalized to internal standards (lipid area / ISTD area), providing relative quantification between samples. PIP3 levels in treated samples (idelalisib or roginolisib) were subsequently expressed as percentage relative to vehicle-treated controls.

To clarify this, the method was changed accordingly:

“Internal standards (PI(5)P[17:0-20:4], #LM1902-1EA; PI(4,5)P_2[17:0-20:4], #LM1904-1EA; PI(3,4,5)P_3 [17:0-20:4], #LM1906-1EA) and running control (PI4P Brain; #840045; PI(4,5)P2 Brain, #840046; PI(3,4,5)P3[18:0-20:4], #850166) were from Avanti Research. The mixture of synthetic PIP/PIP2/PIP3 internal standards was added during the preparation of the samples. The stock solution of internal standards (ISTD) was prepared at 10 ng/µL and diluted to a working concentration of 1 ng/µL. A total of 20 µL of this mixture was added to each sample, corresponding to 20 ng of each internal standard. Running controls with ISTD were separately used as positive controls. All lipid species were normalized to internal standards (lipid area / ISTD area), providing relative quantification between samples. PIP3 levels in treated samples (idelalisib or roginolisib) were subsequently expressed as percentage relative to vehicle-treated controls.”

I am also unsure of how the data is presented with 'PIP3 ISTD/vehicle' on the y-axis- is this clarified in the figure legend which I cannot find in the 'Merged File containing manuscript text and 9 Figure files'? It is not in the methods.

Regarding the y-axis labeling (“PIP3 ISTD/vehicle”), we agree it is confusing. It is now labelled PIP_3 (% of VEHICLE). Besides, this sentence was added in the legend; “ISTD-normalized PIP3 levels in treated samples (idelalisib or roginolisib) were subsequently expressed as percentage relative to ISTD-normalized vehicle-treated controls. The dotted line represents values of 100% of the vehicle-treated control.“

Point-by-point letter addressing reviewer's concerns

Reviewer #3: Thank you for the clarification of your methodology which is now much clearer. While I agree that odd acyl chains (17:0) are normally present at lower abundances than even chains, I am concerned about the abundance of naturally occurring 17:0 acyl chains in your samples. Can you confirm that phosphoinositides containing 17:0 acyl chains are absent in your biological samples, facilitating the use of 17:0 as an internal standard?

Answer: We thank the reviewer for this comment. We confirm that endogenous phosphoinositides containing 17:0 acyl chains were not detected in any of the samples analyzed

The authors propose the structural characterization of the interaction between PI3Kδ (composed of p110δ and p85α) and Roginolisib, a novel small molecule inhibitor that targets the PI3Kδ isoform (currently under phase II trials). This inhibitor shows high target specificity and a safety profile compared to the first generation of PI3Kδ inhibitors such as Idelasilib, used for the treatment of patients with relapsed chronic lymphocytic leukaemia (CLL) and indolent non-Hodgkin lymphoma (NHL). These first-generation inhibitors showed variable toxicity, potentially explained by insufficient selectivity among PI3K isoforms. PI3Kδ undergoes several steps from its autoinhibited form to a fully activated state, including notably a membrane localization through the binding to Ras proteins (to p110δ) and the binding of the phosphorylated tyrosine motif of RTK to p85's SH2 domain. Here, the authors used a combination of orthogonal approaches including crystallography, Molecular Dynamics simulations, HDX-MS, protein-lipid FRET assays and MS-based quantification of PIP3 to decipher the inhibition mechanism of PI3Kδ by Roginolisib and explain the high selectivity for the p110δ isoform. Overall, the paper meets the requirements for a publication in this journal after addressing the comments described below.

The first part of the paper shows the structure elucidation involving the p110δ subunit bound to Roginolisib and its comparison with the structure of p110δ bound to Idelasilib, highlighting two different binding modes. Unlike Idelasilib, Roginolisib does not occupy the specificity pocket but binds some residues of it and engages the Asp911 of the DFG motif (involved in binding selectivity) in a DFG-in like conformation.

COMMENTS:

- **The authors compared both structures of human PI3Kδ/Roginolisib and murine PI3Kδ/Idelalisib and highlighted some differences in the binding mode. Could these differences be due to PI3Kδ's nature difference? Can the authors comment on that?**
- **In Figures S1 and S2, labelling the specific residues mentioned in the text would allow a better appreciation of the described interaction network (in Fig S1: inhibitors' names and residues number could be added and in Fig S2: Asp911 could be labelled).**

To further investigate the inhibition mechanism on the conformation dynamics of the p110δ C-ter region (especially kα12 for which they have no density), the authors performed MD simulations using AlphaFold prediction models of the p110δ subunit bound to either ATP, Roginolisib or Idelasilib: 1) The results showed a difference of the kα11-kα12 angle between the three bound states. 2) kα12 is closer to the active site in Roginolisib bound state, *via* Asp911 forming an aspartate cluster with Asp897 and maintaining electrostatic interactions with K1040 and R1043 (consistent with the auto-inhibitory role of PI3Kδ C-ter region).

COMMENTS:

- **The authors only modelled p110δ subunit for most of the simulations. They did not use p110 and p85 (although, they used both subunits in the following HDX-MS experiments). Can the authors comment on that?**
- **In Figure S4, it would be appreciated to label the specific regions mentioned in the text (especially kα12) and have a clear legend to differentiate between the AlphaFold model and 2Y3A structure.**

- **In Figures 3a and b, it would be appreciated to precise the name of the molecules on top of the structures like the authors did in Figure 2a.**

Then the authors performed HDX-MS experiments to characterize the conformational dynamics of p110δ/p85 heterodimer upon Roginolisib and Idelasilib binding. The authors show that kα12 is protected upon Roginolisib binding only, consistent with the folding of the helix over the active site.

COMMENTS:

- **The description of the HDX-MS experiments is missing in the Methods section. Please provide it for all experiments.**
- **Do the authors plan to submit the HDX-MS data to the Proteomics Identifications Database?**
- **In Fig S6, the deuteration level is shown for all identified peptides of p110δ. However, it is not mentioned for which timepoint in the description.**
- **In Figure 4b: Have the HDX-MS results been exported on the AlphaFold model?**

Next, the authors hypothesized that Roginolisib may target the inactive state of p110δ. To test it, they generated a Y685A mutation in p85α, part of the regulatory subunit that mediates the inhibitory contact between p110δ-cSH2 and the region connecting kα11 and kα12 (being catalytically more active). Then, they compared H/D exchange between WT and Y685A – where kα12 is deprotected in the mutant - and then characterized the exchange upon the binding of inhibitors. No changes are observed upon Idelasilib binding between the WT and Y685A while upon Roginolisib binding, no protection is observed for Y685A (unlike the WT), suggesting that Roginolisib makes additional contacts with the C-ter region in the inactive state, limiting kα12 flexibility upon activation.

COMMENTS:

- **Have the authors observed some changes in the region of p85α spanning Y685 in the previous HDX-MS experiment?**
- **Have the authors compared the binding between the inhibitors and WT/Y685A?**

Next, the authors compared the C-ter region between the different p110δ isoforms highlighting the presence of K1040 and R1043 only in p110δ and β isoforms - consistent with IC50 measurements - explaining the high selectivity of Roginolisib towards the δ isoform and in a lesser extent the β isoform.

COMMENT:

- **Do the authors have an idea explaining the IC50 difference (20 fold) between the δ and β isoform given that both have K1040 and R1043 residues?**

Then the authors investigated if the presence of inhibitors affects PI3Kδ membrane interaction. They performed protein-lipid FRET and observed a decrease of 20% of membrane affinity in presence of inhibitors, showing that conformational changes in kα1-kα2 and kα12 upon inhibitor binding have a limited impact on lipid interactions. They next performed MD simulations of p110δ + ATP binding PIP2 containing leukocytic plasma membrane, leading to the insertion of kα12 into the membrane (being more ordered). The simulations in presence of a Δkα12-p110δ construct showed that p110δ remained bound to membrane (*via* the C2 domain).

COMMENTS:

- **In Figures 6a, 6b and 7a, it would be appreciated if the authors could label the mentioned regions and kα12.**
- **It is not clear where the authors are showing the results obtained for the simulations made in presence of the Δkα12 p110δ isoform: is it Figure 8?**

Finally, the authors investigated how the binding modes' differences between the inhibitors affect the kinase activity. To do so, the authors quantified by MS the PIP3 generated in cell lysates from CLL patients treated with inhibitors and showed that Roginolisib was more effective in decreasing PIP3 levels, suggesting a more efficient inhibition of the kinase activity for Roginolisib.

COMMENTS:

- **Could the authors give more information about the methods used for the quantification and the statistical validation regarding the SRM approach?**
- **Do the authors plan to submit the MS data to the Proteomics Identifications Database?**

Minor comments:

- **Homogenization of the legend description (especially the sub-figures labelling like a and b in Figure S1).**
- **Homogenization of inhibitors names (with or without a first capital letter)**
- **Homogenization of the word naïve/naive**
- **When necessary (described in the comments), labelling secondary structures elements and regions in the figures where there are structures would be helpful for the reader.**
- **A bracket is missing l.229 in the main text.**
